# Plasma lipidome is dysregulated in Alzheimer’s disease and is associated with disease risk genes

**DOI:** 10.1038/s41398-021-01362-2

**Published:** 2021-06-07

**Authors:** Yue Liu, Anbupalam Thalamuthu, Karen A. Mather, John Crawford, Marina Ulanova, Matthew Wai Kin Wong, Russell Pickford, Perminder S. Sachdev, Nady Braidy

**Affiliations:** 1 Centre for Healthy Brain Ageing (CHeBA), School of Psychiatry, University of New South Wales, Sydney, Australia; 2 Guangdong Mental Health Center, Guangdong Provincial People’s Hospital, Guangdong Academy of Medical Sciences, Guangzhou, Guangdong China; 3 Neuroscience Research Australia, Randwick, Australia; 4 Mark Wainwright Analytical Centre, University of New South Wales, Sydney, Australia; 5 Neuropsychiatric Institute, Euroa Centre, Prince of Wales Hospital, Sydney, Australia; 6 School of Medicine, Huzhou University, Huzhou Central Hospital Huzhou, Huzhou, China

**Keywords:** Molecular neuroscience, Neuroscience

## Abstract

Lipidomics research could provide insights of pathobiological mechanisms in Alzheimer’s disease. This study explores a battery of plasma lipids that can differentiate Alzheimer’s disease (AD) patients from healthy controls and determines whether lipid profiles correlate with genetic risk for AD. AD plasma samples were collected from the Sydney Memory and Ageing Study (MAS) Sydney, Australia (aged range 75–97 years; 51.2% male). Untargeted lipidomics analysis was performed by liquid chromatography coupled–mass spectrometry (LC–MS/MS). We found that several lipid species from nine lipid classes, particularly sphingomyelins (SMs), cholesterol esters (ChEs), phosphatidylcholines (PCs), phosphatidylethanolamines (PIs), phosphatidylinositols (PIs), and triglycerides (TGs) are dysregulated in AD patients and may help discriminate them from healthy controls. However, when the lipid species were grouped together into lipid subgroups, only the DG group was significantly higher in AD. ChEs, SMs, and TGs resulted in good classification accuracy using the Glmnet algorithm (elastic net penalization for the generalized linear model [glm]) with more than 80% AUC. In general, group lipids and the lipid subclasses LPC and PE had less classification accuracy compared to the other subclasses. We also found significant increases in SMs, PIs, and the LPE/PE ratio in human U251 astroglioma cell lines exposed to pathophysiological concentrations of oligomeric Aβ_42_. This suggests that oligomeric Aβ_42_ plays a contributory, if not causal role, in mediating changes in lipid profiles in AD that can be detected in the periphery. In addition, we evaluated the association of plasma lipid profiles with AD-related single nucleotide polymorphisms (SNPs) and polygenic risk scores (PRS) of AD. We found that *FERMT2* and *MS4A6A* showed a significantly differential association with lipids in all lipid classes across disease and control groups. *ABCA7* had a differential association with more than half of the DG lipids (52.63%) and PI lipids (57.14%), respectively. Additionally, 43.4% of lipids in the SM class were differentially associated with *CLU*. More than 30% of lipids in ChE, PE, and TG classes had differential associations with separate genes (ChE-*PICALM*, *SLC24A4*, and *SORL1*; PE-*CLU* and *CR1*; TG-*BINI*) between AD and control group. These data may provide renewed insights into the pathobiology of AD and the feasibility of identifying individuals with greater AD risk.

## Introduction

Alzheimer’s disease (AD) is the most common cause of dementia, accounting for about 70% of total cases^1^. This progressively neurodegenerative disease is characterized by an insidious onset and is clinically defined by a progressive loss of memory and other cognitive deficits. AD has become one of the major challenges for the public health and economic system of the 21st century. There is still no international consensus on the etiology of this multifactorial disease, in which in addition to proteinopathies, oxidative stress, inflammation, metabolic disorder, and other factors play a part^2–4^.

The lack of effective treatments and the potential for prevention highlight the importance of identifying early biomarkers for diagnosing AD. In addition, there is evidence that pathological processes associated with AD can also manifest in the peripheral system^5^, indicating the possibility of identifying non-invasive blood biomarkers. Lipids participate in important functions such as cell membrane formation, cellular transport, and energy storage, and act as essential signaling molecules. Beyond their structural roles, lipids have also been shown to act as modulators of transmembrane proteins, such as ion channels, whereby alteration of the composition or conformation of lipids surrounding ion channels can affect their function^6,7^. Given the essential role of lipids in major biological processes, blood lipids have emerged as promising biomarkers for AD^8–10^.

Although there have been many studies on the association between lipids and the pathobiology of AD, there are few studies on the plasma lipidome in AD. In contrast to classical biochemical approaches that focus on single metabolites or reactions, lipidomics approaches simultaneously identify and quantify hundreds of lipids. Measurement of large numbers of lipids enables network analysis approaches and provides means to identify critical metabolic drivers in disease pathophysiology. Lipidomics provides powerful tools for mapping global biochemical changes in disease and treatment.

In our current study, we examined differences in the plasma lipidome between AD and ‘healthy’ age-matched controls and compared the ability of different lipid profiles to discriminate between the two groups. We also showed that human astroglioma cultures exposed to pathological levels of amyloid-beta (Aβ)_42_ oligomers shared similar cellular lipidomic profiles to those observed in human plasma AD. We explored the effect of AD polygenic risk scores and AD-related SNPs on plasma lipid levels between AD and controls.

## Method

### Participants

Participants were a subsample from the population-based longitudinal Sydney Memory and Ageing Study (Sydney MAS)^11^, an ongoing study that began in 2005 and focuses on cognitive decline in community-dwelling elderly. Participants were aged 70–90 years, initially without dementia, living in the community, and able to complete their assessments in English. There have been four Waves of data collection, two years apart. At each Wave, participants underwent an MRI scan, comprehensive neuropsychological assessment, medical examination, and blood collection for biochemistry analyses and DNA extraction. Written informed consent was obtained from all participants. In this study, AD (diagnosed by NINCDS-ADRDA criteria) and cognitively normal control samples were collected at Waves 2 and 4. Ethics approval for this study was obtained from UNSW Sydney Australia and the South-Eastern Illawarra Area Health Service—Eastern sector^11^. The investigators were blinded to sample allocation during the study and outcome assessment.

### Plasma lipid extraction

Plasma lipids were extracted as previously described^12^. Briefly, 10 µL internal lipid standards (ISTDs) (Avanti lipids, https://avantilipids.com/product/330707) were added to 10 µL aliquot of each plasma sample. 100 µL of 1-butanol-Methanol (1:1 v/v) containing 5 mM ammonium formate were used to dissolve the mixture. Samples were vortexed for 10 s then sonicated for one hour. Afterward, samples were centrifuged at 13,000 × *g* for 10 min. The supernatant was transferred into a fresh Eppendorf tube. A further 100 µL of 1-butanol/methanol (1:1 v/v) with 5 mM ammonium formate was added to the white pellet to re-extract any remaining lipids. The supernatant was dried in a speed vacuum centrifuge for 40–60 min. The lipids were reconstituted by adding 100 µL of 1-butanol/methanol (1:1 v/v) containing 5 mM ammonium formate to each tube. The contents were transferred into a 300 µL glass Chromacol vial with a glass insert prior to liquid chromatography/mass spectrometry (LC–MS).

### Liquid chromatography/mass spectrometry

Lipid analysis was performed by LC ESI-MS/MS using a Thermo QExactive Plus Orbitrap mass spectrometer as previously described^12^. Briefly, a Waters ACQUITY UPLC CSH^TM^ C18 1.7 μm, 2.1 × 100 mm column was used for liquid chromatography at a flow rate of 260 gl/min, using the following gradient condition: 32% solvent B to 100% over 25 min, a return to 32% B and finally 32% B for 5 min prior to the next injection. Solvents A and B consisted of acetonitrile: MilliQ water (6:4 v/v) and isopropanol:acetonitrile (9:I v/v) respectively, both containing 10 mM ammonium formate and 0.1% formic acid. The first 3 min of eluent, containing the eluted salts, was diverted to waste. A product ion scan in positive and negative ion modes was performed to analyze the individual lipid species. The order of sampling was randomized prior to analysis. Ceramide (Cer), sphingomyelin (SM), phosphatidylcholines (PC), phosphatidylethanolamines (PE), phosphatidylinositol (PI), lyso-phosphatidylcholines (LPC), cholesterol esters (ChE), diacylglycerol (DG) and triacylglycerols (TG) were detected. The abundance of lipids was acquired using Lipidsearch software version 4.2 (Thermo Fisher Scientific, Sydney, NSW AU) according to accurate lipid mass and fragment matching^12^. The LC–MS data were exported into Microsoft Excel and normalized by dividing the abundance of internal standards to be used for further statistical analyses.

### Cell lipidomics

#### Cell culture

U251 human astroglioma cell lines were purchased from the ATCC. These cells were cultivated in Roswell Park Memorial Institute (RPMI) 1640 Medium supplemented with 10% fetal bovine serum, 1% 1-glutamax, and 1% antibacterial/antifungal. The cells were recently tested and found to be mycoplasma-free using the MycoAlert Mycoplasma Detection Kit (Lonza). The cells were grown at 37 °C in 95% humidified air and 5% CO_2_. The culture medium was replaced every 2 days. U251 cells were seeded in a 12-well microtitre plate and given treatment when cells were nearly at confluency (0.5 × 106 cells per well). Groups for evaluation are as follows: 6 wells containing naive U251 cells with no treatment (control) for 24 h, and 6 wells containing U251 cells treated for 24 h with 5 µM oligomeric Aβ_42_ (see below for further details).

#### Preparation of recombinant Aβ_42_ peptide

Aβ_42_ peptide was purchased from Recombinant Peptide Technologies (Athens, GA, USA). The peptide was immediately stored in sealed glass vials at −80 °C in a lyophilized form. Consistent homogenous preparations of recombinant Aβ_42_ oligomers or fibrils for use in cell culture stimulation experiments were obtained following a previously published protocol^13^. To avoid condensation upon opening, each vial was left at room temperature for 30 min prior to resuspension. Using 1,1,1,3,3,3-hexafluoro-2-propanol (HFIP; Sigma, Castle Hill, Australia), the lyophilized peptide was initially dissolved to 1 mM and separated into 50 μl aliquots in sterile microcentrifuge tubes. Aliquots were left for 3 h in a fume hood, which allowed almost complete evaporation of HFIP. The resulting peptide films were further dried under vacuum using a Speed Vac (ThermoSavant, Patterson, CA, USA). This procedure is important as HFIP evaporated Aβ will form fibrils if exposed to moisture in the air, hence it is paramount to store HFIP evaporated Aβ films in desiccant. These preparations were then stored at −20 °C until required.

#### Production of recombinant Aβ_42_ oligomers

Each HFIP aliquot of the peptide film was thoroughly resuspended to 5 mM in anhydrous dimethyl sulfoxide (DMSO; Sigma, Castle Hill, Australia) via pipette mixing, followed by 10-min bath sonication (Model: FX8, Unisonics, Sydney, Australia). Oligomers were formed by adding ice-cold Dulbecco’s modified Eagle medium/Ham F-12 without phenol red (Sigma, Castle Hill, Australia) to a final concentration of 100 μM. Following a 30 s vortex, the preparation was incubated at 4 °C for 6 weeks for the formation of Aβ_42_ oligomers.

#### Cell lipidomics

Samples were collected using a cell scraper to 1 mL PBS solvent. After centrifuging 5 min at 13,000 × *g*, cell pellets were collected and reconstituted in 110 µL MilliQ water. 10 µL of cell solution was used for protein assay and the rest was used for lipid extraction. Lipids extraction and LC–MS methods were the same as plasma lipids, which were described above. 10 lipid classes including Cer, SM, PC, PE, PI, LPC, lyso-phosphatidylethanolamines (LPE), ChE, DG, and TG were detected.

### Quantification of oligomeric Aβ

Oligomeric Aβ was quantified as previously described^14^. Briefly, plasma samples were thawed at 37 °C for 15 min. Afterwards, 10 μl of plasma, 4 μl of HAMA (human anti-murine antibody, HAMA) blocker (Scantibodies Laboratory, Santee, CA, USA), were mixed. 10 μl of PBR-1 (1% proprietary + 1.25% dimethyl sulfoxide (DMSO) + 96.75% phosphate-buffered saline contains Tween 20 (PBST) + 1% ultra-pure water) were further mixed into the plasma mixture. The mixtures were incubated for 48 and 1 h, respectively. The plasma sample mixture and serially diluted standards were added to separate wells of the plate in a total volume of 100 μl. The plates were incubated at RT for 1 h. The detection antibody was added to the wells, and the plate was incubated for 1 h at RT. Finally, 100 μl of 3,3′,5,5′-tetramethylbenzidine (TMB) reagent was added as a substrate, and after 15 min, the reaction was stopped with 50 μl of 1 M H_2_SO_4_. Optical density (OD) values were measured using the BMG Fluostar Optima multimode plate reader (NY, USA), at a wavelength of 450 nm. Prior studies using this method detected the raw luminescence signal and used relative luminescence units (RLU) to present the oligomeric Aβ levels^15^.

### Genotyping

DNA was extracted using standard procedures. Genotyping of the *APOE* ε2/3/4 polymorphism was performed as described^16^. Genome-wide genotyping was undertaken using the Affymetrix Genome-wide Human SNP Array 6.0 (California, USA) at the Ramaciotti Centre, UNSW Australia^17^. The CRLMM package (v1.10.0) in R (v2.12.1) was used to call genotypes. SNPs were excluded if the genotyping call rate was <95%, had a minor allele frequency <0.01 or if they failed a Hardy–Weinberg equilibrium threshold of <1 × 10^−6^. After further QC checks, there were 925 Sydney MAS participants with data for 734,550 SNPs. Imputation was undertaken to the 1000 Genome reference panel using the Michigan Imputation Server. SNPs with poor imputation quality were omitted from any further analyses (*R*^2^ ≤ 0.6).

### Polygenic Risk Scores (PRS) and AD implicated SNPs

PRS were generated using the PRSice program^18^ from summary statistics obtained from a previous Alzheimer’s disease GWAS^19^. Linkage disequilibrium pruning was performed using the clumping option (*r*^2^ > 0.25 and physical distance threshold of 250 kb KB). We present the association of lipids with the PRS calculated using the SNPs with the AD GWAS *p*-value threshold ≤5 × 10^−5^. Associations between 33 individual AD-related SNPs^20^ that passed QC checks (SNPs with MAF > 0.05 and imputation quality >0.6) and lipids were also undertaken. Details regarding the SNPs and their associated genes utilized in the analyses are provided in Supplementary Table 1.

### Statistical methods

#### Comparison of lipids between AD and controls

Inverse normal transformed residuals for the individual lipids and group lipids were obtained after regressing out the effect of possible confounders: age, sex, BMI, diabetes status, hypertension status, medication status for hypertension and hyperlipidemia, *APOE* e4 carrier status, education, and current smoking status. This transformed data was used in all subsequent analyses. *T*-tests were used to compare the mean value of lipids residuals between AD and controls. We used a significance threshold of 0.05 after False discovery rate (FDR) correction for all comparisons. Fold change (FC) was calculated as the ratio of the average lipids abundance in AD and controls. *T*-tests were also applied to compare lipid abundance between Aβ treated and untreated cells. Between-group comparisons are done assuming unequal variance between groups with an approximation for degrees of freedom.

#### Classification of AD vs. Control using GLMnet

We used a machine-learning algorithm, glmnet (elastic net penalization for the generalized linear model [glm]) to classify AD versus control samples. A combination of two penalty functions with two tuning parameters was utilized to shrink the beta coefficients in the glm^21^. R (version 3.5.1)^22^ package caret^23^ for fitting the elastic net glm model with default options was used to identify the optimum values for the tuning parameters.

For classification analysis, the data were randomly split into 70% training and 30% test samples maintaining the proportion of cases and controls in the training and test samples as in the full dataset. For the training data, the algorithms were run with three cross-validations with five repeats. To avoid bias due to a single random split of the original data, we have repeated the analysis 10 times and the results were summarized over the 10 iterations.

The glmnet classification accuracy was examined based on several subgroups of the lipid species. The receiver operating curve (ROC) and area under ROC (AUC) were obtained using the R package pROC^24^. Average sensitivity (proportion of AD cases predicted by the model in the test data), specificity (proportion of controls predicted by the model in the test data), and the AUC across 10 iterations are reported.

#### Genetic variation and lipid profiles among AD vs. controls

Linear regression was used to examine the association of AD risk variants and AD PRS with the lipids. The inverse normal transformed lipid residuals were used as the dependent variable and individual SNPs or PRS, case-controls (CC) status (AD vs. controls), and the relevant interaction term (CC × SNP or CC × PRS) were used as independent variables. Differential association of the AD risk variants and the PRS with lipids among cases and controls were examined based on the significance of the interaction term.

## Results

### Descriptive statistics

The sample comprised 82 plasma samples (40 AD patients and 42 cognitively normal ‘healthy’ controls) from the Sydney MAS cohort. The demographic information of participants is displayed in Table 1. The AD patients were significantly older than the controls with all aged more than 75 years old. There were no gender differences between the 2 groups (*χ*^2^ = 1.233, *p* = 0.267). As expected, cognitive scores on the Mini-Mental State Examination (MMSE) were significantly lower in AD patients. Education levels, which represent a protective factor for AD were measured in years and showed no statistical difference between the AD and control groups. AD and controls did not differ in vascular risk factors, including frequency of hypertension, diabetes, and current smoking status. Carriers of the APOE*4 allele, a strong genetic risk factor for AD risk, were more common in AD patients than controls, although this did not reach statistical significance (AD 37.5% vs. control 19%, *χ*^2^ = 3.457, *p* = 0.063). The elevated levels of oligomeric Aβ in the plasma distinguished the AD and control groups and were associated with increased MMSE, in patients with AD.

**Table 1:** Characteristics of Alzheimer’s disease and control participants.

	Healthy controls (*n* = 42)	Alzheimer’s disease (*n* = 40)	Difference
Age(years)	81.27(2.48)	86.72(5.03)	*t* = 6.270, *p* < 0.01
Sex (*males/females)*	19/23	23/17	*χ*^2^ = 1.233, *p* = 0.267
Oligomeric Aβ (OD value)	0.25(0.05)	0.40(0.10)	*t* = 6.391, *p* < 0.01
Education (years)	11.30(3.59)	11.59(3.64)	*t* = 0.363, *p* = 0.717
Body mass index	26.69(3.19)	25.45(3.72)	*t* = 1.631, *p* = 0.107
Diabetes	7.10%	15%	*χ*^2^ = 1.294, *p* = 0.255
Hypertension	61.90%	77.50%	*χ*^2^ = 2.351, *p* = 0.125
Anti-hypertensive medication	60.00%	54.30%	*χ*^2^ = 0.249, *p* = 0.618
Anti-hyperlipidemia medication	64.30%	50.00%	*χ*^2^ = 1.709, *p* = 0.791
Current smoker	9.50%	5.00%	*χ*^2^ = 0.618, *p* = 0.432
APOE*4 carriers	19%	37.50%	*χ*^2^ = 3.457, *p* = 0.063
MMSE	28.21(1.58)	22.63(3.86)	*t* = 8.557, *p* < 0.01

Continuous variables are expressed as means and standard deviations. Categorical variables are expressed as %.

APOE*4 apolipoprotein epsilon 4, *MMSE* Mini-mental state examination.

### Comparison of lipids between AD and control patients

**Fig. 1:**
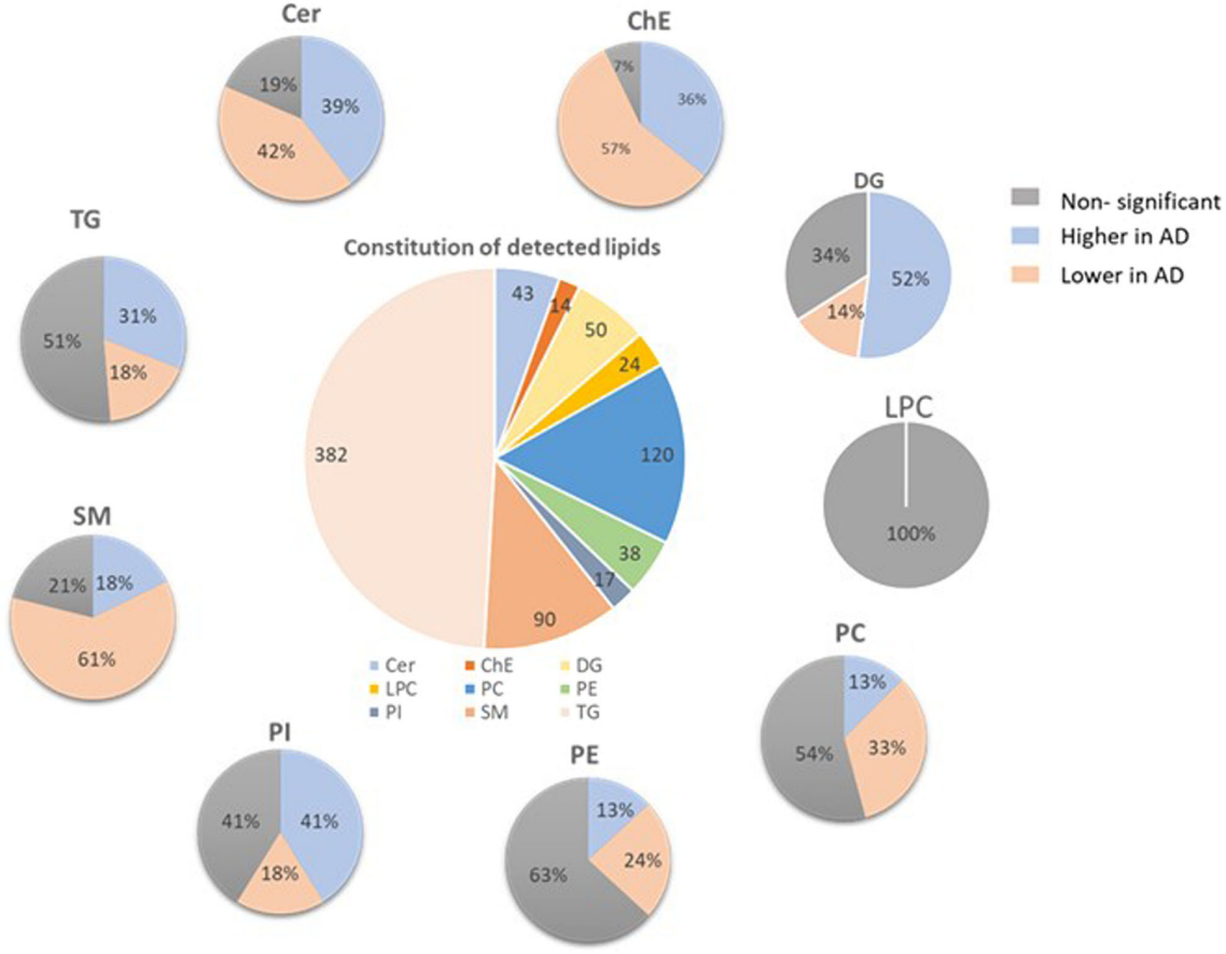
Proportion of lipids significantly different between AD and control in lipid classes. Overall pie chart presenting the number of lipids measured in each lipid class. Separate pie charts describing the proportion of lipids with non-significant differences or significantly higher/lower in AD versus controls for each lipid class. Cer ceramides, SM sphingomyelins, ChE cholesteryl esters, DG diglycerides, TG triglycerides, PC phosphatidylcholines, LPC lyso-phosphatidylcholines, PE phosphatidylethanolamines, PI phosphatidylinositols.

**Fig. 2:**
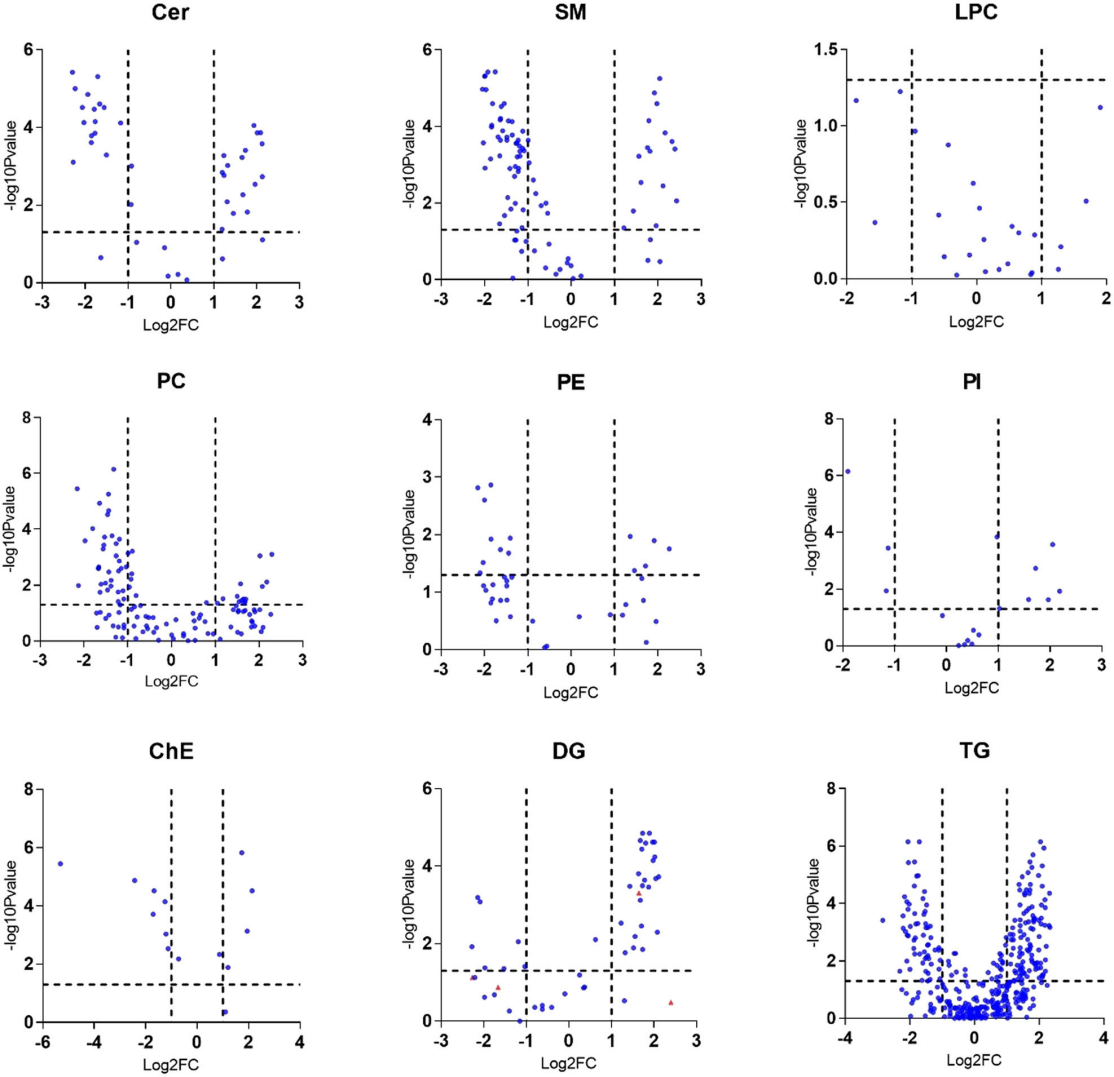
Volcano plots showing significant lipid species in the nine separate lipid groups. Each dot on the plot is a single lipid species. Horizontal axis: fold change (in log2 scale); vertical axis: adjusted *p*-value (in log10 scale). Vertical dashed lines highlight log2 fold changes of −1 and +1, while a horizontal dashed line represents a *p*-value of 0.05. Cer ceramides, SM sphingomyelins, ChE Cholesteryl esters, DG diglycerides, TG triglycerides, PC phosphatidylcholines, LPC lyso- phosphatidylcholines, PE phosphatidylethanolamines, PI phosphatidylinositols.

A total of 778 distinct lipid species from 9 lipid classes were analyzed, including neutral lipids such as 14 cholesteryl esters (ChE), 50 diglycerides (DG), 382 triglycerides (TG); sphingolipids including 43 ceramides (Cer), 90 sphingomyelins (SM); and phospholipid subclasses including 120 phosphatidylcholines (PC), 24 lyso-phosphatidylcholines(LPC), 38 phosphatidylethanolamines (PE), and 17 phosphatidylinositols (PI). The proportion of lipids significantly different between AD and controls according to each of the examined lipid classes are shown in Fig. 1. Volcano plots were drawn according to lipid fold changes of abundance comparing AD and controls and FDR-corrected *p* values in for all individual lipid species (see Fig. 2 and Supplementary Table 2).

Almost all ceramides were significantly different between control and AD groups after adjustment for age, sex, and vascular risk factors, even after multiple testing corrections. Molecular profiles of the ceramidome showed that dihydroxy Cer species comprising fatty acyls (18C) with 0 or 2 double bonds and all dihydroxy Cer with 19C were significantly higher in AD. On the other hand, dihydroxy Cer containing 16C or 18C with 1 double bond showed the opposite results. Most monohydroxy and trihydroxy Cer were significantly lower in AD subjects. Four AD positive ceramides including Cer(d18:0_16:0), Cer(d19:1_24:0), Cer(d18:0_23:0), and Cer(d18:2_25:0), and AD negatively correlated ceramides: Cer(d18:1_23:0), Cer(t16:1_14:0), Cer(m18:1_20:0), Cer(m18:0_22:0), and Cer(d19:1_22:0) showed the largest fold changes (FC > 4). Sphingomyelin is another type of sphingolipid. Most lipids in SM class showed significantly lower levels in AD plasma. The lipids with FC > 4 included SM(d35:4), SM(d34:1), SM(d31:1), SM(d18:1_21:0), SM(t36:2), and SM(d40:4). In contrast, SM(d41:1), SM(d41:4), and SM(d18:1_24:3) were largest downregulated in AD.

In the AD cases, there was a higher abundance of PC lipids compared to controls. They were comprised of very short and short-chain fatty acyls except for PC containing C15 without double bonds and C16 with 2 double bonds. The largest fold changes were observed in PC(16:0_22:6), PC(18:2_18:2), PC(18:0_20:4), PC(36:2), PC(16:1_22:5) that showed upregulation in AD. PC (20:2_18:2) and PC (38:7) showed lower plasma concentrations in AD. LPCs with longer fatty acid chains tended to be lower in AD patients. No LPC lipids differed significantly between cognitively normal and AD groups. Most of PE lipids were lower in AD-affected patients. Major PE lipids were reduced in AD, including highly changed (FC > 4) PE (16:0p_22:6), PE(18:0p_20:4), PE(16:0p_18:1), and PE(18:0p_22:4). PE (18:0_18:1) was higher in plasma of Alzheimer’s patients. PI showed an elevated abundance in AD and the PI lipids, with PI (18:0_18:3) and PI (18:1_20:4) the most significantly elevated in AD cases.

ChE (18:3), ChE (20:3), and ChE (22:3) showed the largest fold changes. Consistent with the result of group DGs, most DG lipids species were higher in AD patients, including DG(16:0_18:3), DG(22:4e), DG(17:1_18:1), DG(20:0_18:2), and DG(36:4e) with largest fold changes. Most downregulated DGs include DG(18:1_20:4), DG(16:0_18:1), and DG(18:0_18:1).

**Fig. 3:**
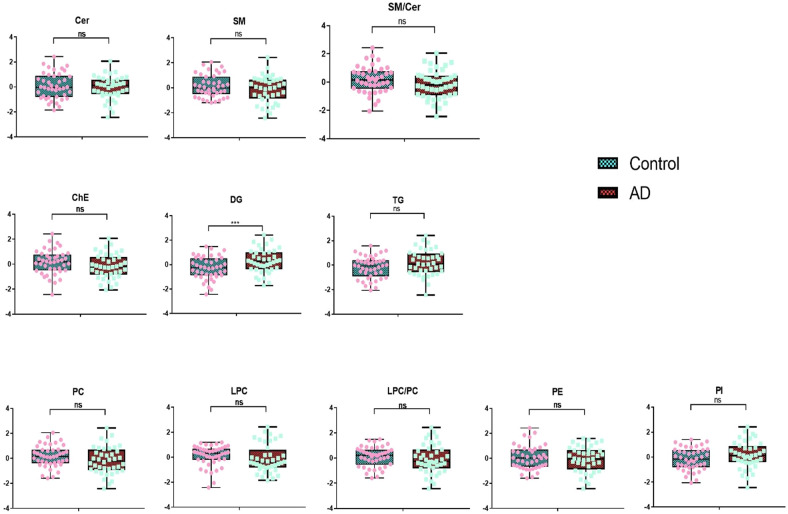
Residuals of group lipids in AD and control group. Cer ceramides, SM sphingomyelins, ChE cholesteryl esters, DG diglycerides, TG triglycerides, PC phosphatidylcholines, LPC lyso-phosphatidylcholines, PE phosphatidylethanolamines, PI phosphatidylinositols. Dots in the plot represent the residuals of group lipids in each participant. Residuals were obtained after regression out age, sex, BMI, diabetes status, hypertension status, medication status for hypertension, and hyperlipidemia, *APOE* e4 carrier status, education, and current smoking status. *T*-tests were used to compare the lipid differences between AD and controls. ns: no significance, *p < 0.05.

As well, around 30% of TG lipids were observed to be higher in AD patients. The most upregulated TG lipids include TG(18:1_17:1_18:3), TG(14:0_18:2_20:5), TG(16:1_20:1_22:4), TG(60:10), and TG(18:1_18:1_22:5). On the other hand, most of the long-chain polyunsaturated fatty acid-containing TGs were reduced in AD compared to age-matched controls. About 18% of plasma TG lipids are decreased in AD patients, and TG(18:1_12:0_14:0), TG(18:4_16:1_18:3), TG(16:0_20:4_22:6), TG(16:0_14:0_18:1), and TG(16:0_16:0_16:0) showed the largest fold changes. We also identified suggestive group differences between almost all species containing more than 2 double bonds. Nine lipid groups calculated by summarizing all lipid species in the same lipid class were compared between AD and controls (Fig. 3 and Supplementary Table 3). Only the DG group was significantly higher in AD (*p* = 0.009, mean of residuals: Control: −0.276 ± 0.143, AD:0.290 ± 0.154). Other lipid group’s lipids did not differ after adjusting for covariates.

### Cell lipidomics

**Fig. 4:**
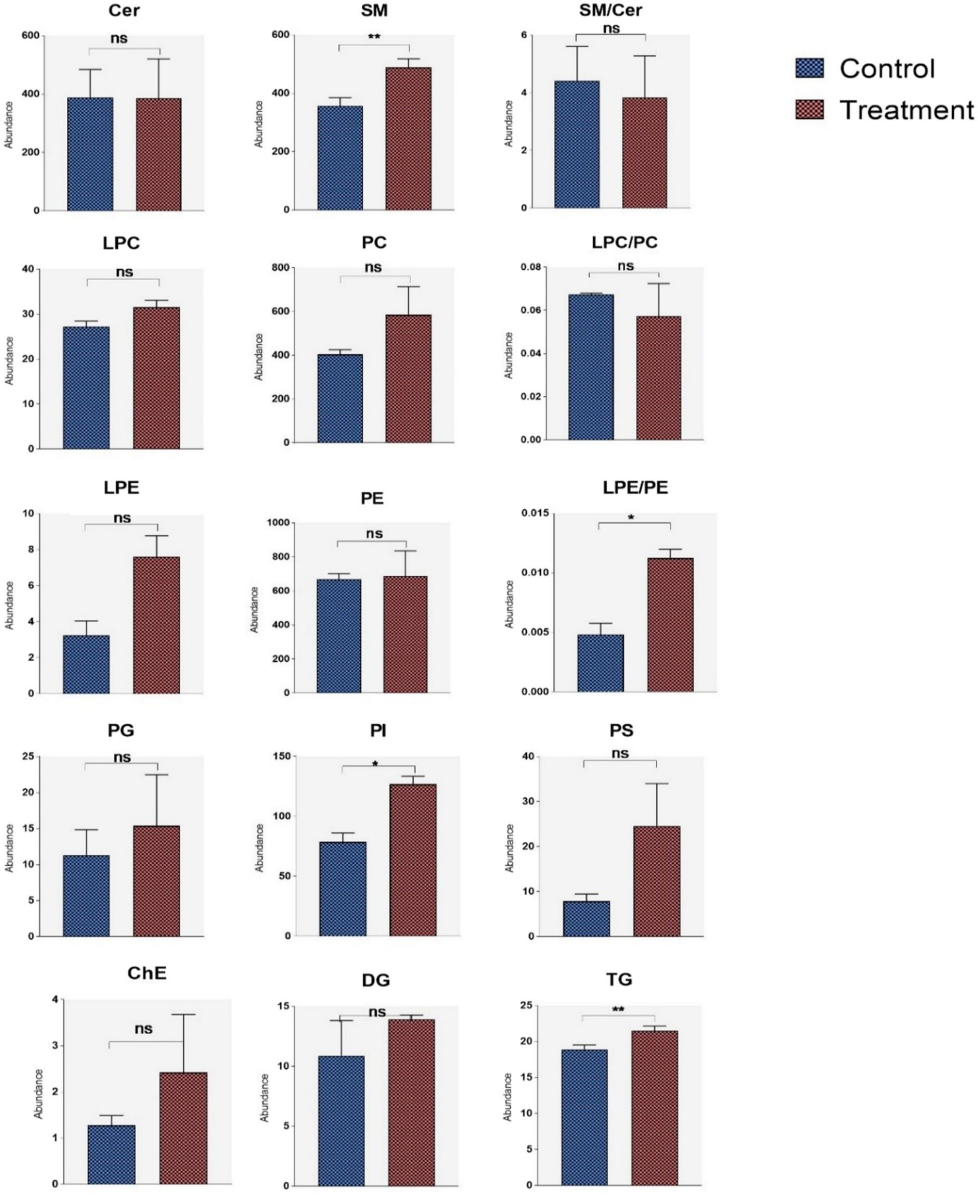
Normalized lipid group abundance in amyloid beta-treated astrocytes versus controls. Cer ceramides, SM sphingomyelins, ChE Cholesteryl esters, DG diglycerides, TG triglycerides, PC phosphatidylcholines, LPC lyso-phosphatidylcholines, PE phosphatidylethanolamines, PI phosphatidylinositols. *T* tests were used to compare lipids between two groups. ns: no significance, *p < 0.05, **p < 0.01.

Results are plotted in Fig. 4. The concentration of oligomeric peptide used in this study is similar to that reported from human CSF and human cell culture conditioned medium^25,26^. Additionally, it also represents a concentration closer to the levels found in brain^25,26^ offering a more physiologic impression of the effect of Aβ oligomers on astrocyte glial lipid profiles. SMs were significantly elevated in astroglioma cells treated with Aβ_42_ oligomers compared to non-treated cells (*p* = 0.0015). No significant differences were found in phospholipids except that the level of the PI group was higher in the Aβ_42_ group (*p* = 0.041), which is consistent with the result found in human AD plasma samples. Even though PE and LPE were not significantly different between treated and non-treated cells, the ratio of LPE/PE significantly increased in Aβ_42_-treated astrogliomas (*p* value). TG was also increased in the Aβ_42_ group (*p* = 0.005).

### Classification of AD vs. Control using lipid profiles

**Fig. 5:**
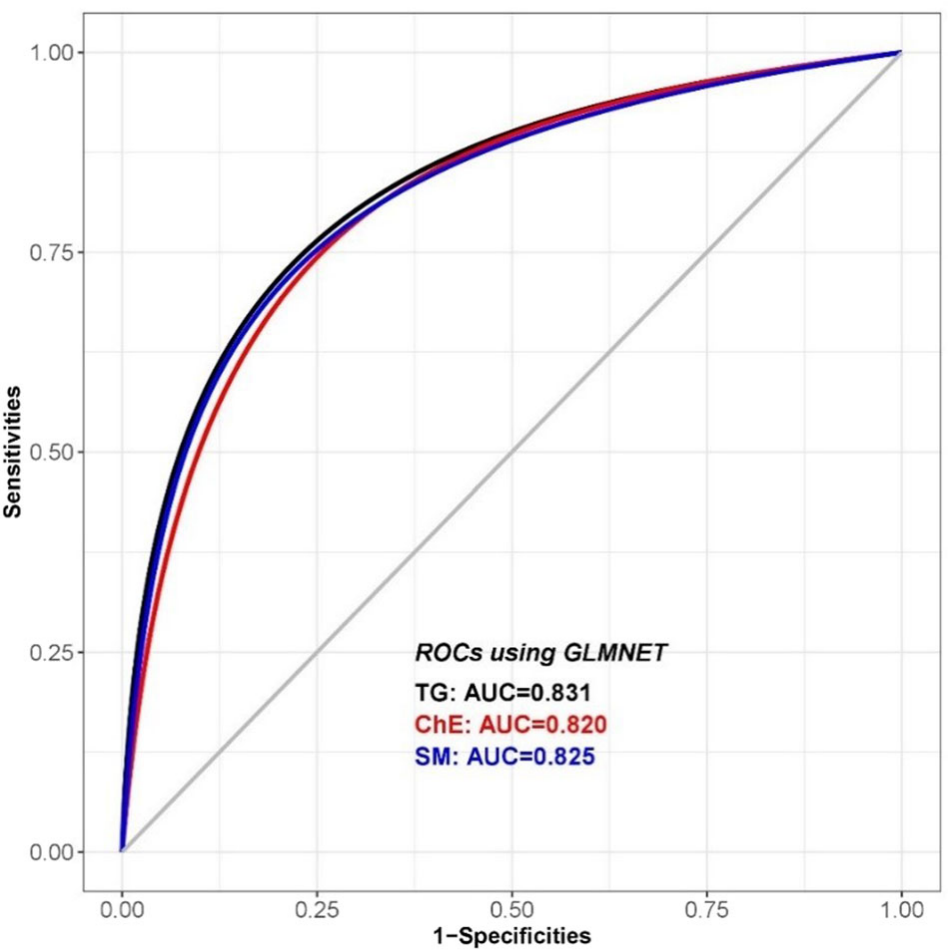
ROC of TG, ChE, and SM lipid models in the classification of AD versus control by GLMnet. SM sphingomyelins, ChE cholesteryl esters, TG triglycerides, GLMnet elastic net penalization for the generalized linear model.

Several models were used for the classification of AD versus controls based on the plasma lipidome profile. The glmnet package was used in all the analyses. The analyses were repeated 10 times and results were summarized across the 10 iterations. The algorithms were run in turn using the full list of individual lipids, all of the 9 subclasses of lipids, and group lipids. The average sensitivity, specificity, and AUC of the test data are summarized in Table 2. The ROC curves for the top 3 models, ChE, SM, and TG are presented in Fig. 5 and resulted in good classification accuracy with more than 80% AUC. In general, group lipids and the lipid subclasses LPC and PE had less classification accuracy compared to the other subclasses.

**Table 2:** GLMnet results of lipid models in the classification of AD versus control.

Lipid models	Sensitivity	Specificity	AUC
All_lipids	0.66	0.73	0.78
Cer	0.7	0.79	0.79
ChE	0.78	0.75	0.82
DG	0.72	0.69	0.76
LPC	0.49	0.62	0.58
PC	0.69	0.76	0.78
PE	0.58	0.5	0.57
PI	0.7	0.76	0.79
SM	0.71	0.78	0.83
TG	0.65	0.82	0.83
SM_TG	0.69	0.75	0.81
ChE_SM	0.7	0.81	0.79
ChE_TG	0.71	0.78	0.82
ChE_SM_TG	0.67	0.77	0.79

*Cer* ceramides, *SM* sphingomyelins, *ChE* cholesteryl esters, *DG* diglycerides, *TG* triglycerides, *PC* phosphatidylcholines, *LPC* lyso-phosphatidylcholines, *PE* phosphatidylethanolamines, *PI* phosphatidylinositols, *GLMnet* elastic net penalization for the generalized linear model.

### SNPs and lipids with AD versus control

The associations of AD PRS and AD-related SNPs with the lipidome were examined. The individual lipids that were significantly different between AD and controls (*n* = 420) were utilized to explore the association. No significant association of AD PRS with lipids was observed after FDR correction (Supplementary Table 4). However, the AD PRS × CC interaction was nominally significant (*p*-value < =0.05) for 57 lipid species (Table 3), which were mainly PCs, SMs, and TGs. Out of the 57 significant interactions, only two PRS scores had marginal significant effects (*p* value < 0.05). The same analysis was also repeated using 33 AD implicated individual SNPs recently identified in another study^20^. All of these individual SNPs were found to have different effects on a certain number of lipids between AD and controls before adjustment for multiple testing. Nominally significantly different associations of SNPs with lipids between AD and controls are listed in Supplementary Table 5.

**Table 3:** Nominally significantly differential association of the AD risk PRS with lipids between AD and controls control.

Lipid	Beta.PRS	Beta.PRS_CC	SE.PRS	SE.PRS_CC	t.PRS	t.PRS_CC	Pval.PRS	Pval.PRS_CC
Cer(d18:1_16:0)	0.197	−0.511	0.133	0.195	1.483	−2.624	0.142	0.011
Cer(t16:1_14:0)	0.268	−0.510	0.141	0.207	1.894	−2.461	0.062	0.016
ChE(22:5)	−0.080	0.539	0.137	0.200	−0.583	2.690	0.562	0.009
PC(16:0_20:4)	−0.107	0.495	0.144	0.211	−0.745	2.345	0.459	0.022
PC(16:0_20:5)	0.001	0.535	0.144	0.210	0.007	2.544	0.994	0.013
PC(16:0_22:6)	0.150	0.419	0.123	0.180	1.221	2.331	0.226	0.022
PC(16:2e_16:0)	0.089	−0.428	0.145	0.212	0.612	−2.018	0.543	0.047
PC(18:0_20:3)	0.131	0.433	0.135	0.198	0.973	2.187	0.334	0.032
PC(18:1_20:3)	0.025	0.441	0.143	0.209	0.174	2.108	0.863	0.038
PC(18:2_18:2)	−0.005	0.415	0.138	0.202	−0.039	2.053	0.969	0.044
PC(34:0)	0.057	−0.510	0.142	0.208	0.404	−2.447	0.688	0.017
PC(37:2)	0.285	−0.488	0.147	0.215	1.942	−2.267	0.056	0.026
PE(16:0_20:3)	−0.059	0.445	0.144	0.210	−0.413	2.117	0.681	0.038
PI(18:0_18:2)	0.026	−0.421	0.123	0.180	0.208	−2.346	0.836	0.022
PI(36:3)	−0.163	0.603	0.148	0.217	−1.105	2.784	0.273	0.007
SM(d17:1_13:0)	0.353	−0.668	0.142	0.209	2.476	−3.200	0.016	0.002
SM(d17:1_18:3)	0.041	−0.405	0.134	0.196	0.303	−2.064	0.763	0.043
SM(d18:2_24:3)	0.240	−0.470	0.145	0.213	1.653	−2.207	0.103	0.030
SM(d32:4)	0.166	−0.585	0.134	0.197	1.233	−2.971	0.222	0.004
SM(d33:1)	0.095	−0.521	0.139	0.204	0.683	−2.551	0.497	0.013
SM(d34:2)	0.132	−0.416	0.141	0.206	0.933	−2.014	0.354	0.048
SM(d35:1)	0.204	−0.462	0.148	0.216	1.384	−2.134	0.171	0.036
SM(d36:3)	0.365	−0.568	0.146	0.213	2.506	−2.662	0.014	0.010
SM(d37:2)	−0.012	−0.429	0.136	0.199	−0.090	−2.161	0.929	0.034
SM(d38:1)	0.006	−0.419	0.127	0.186	0.049	−2.250	0.961	0.027
SM(d38:2)	0.027	−0.458	0.137	0.200	0.197	−2.285	0.844	0.025
SM(d39:1)	−0.031	−0.413	0.131	0.192	−0.240	−2.153	0.811	0.035
SM(d39:2)	0.108	−0.516	0.139	0.203	0.776	−2.539	0.440	0.013
SM(d40:1)	0.038	−0.453	0.135	0.198	0.283	−2.294	0.778	0.025
SM(d40:3)	0.118	−0.458	0.149	0.218	0.789	−2.098	0.433	0.039
SM(d41:4)	−0.070	−0.397	0.135	0.198	−0.520	−2.003	0.605	0.049
SM(d42:1)	0.103	−0.435	0.138	0.202	0.746	−2.152	0.458	0.035
SM(d42:2)	0.028	−0.507	0.129	0.189	0.221	−2.688	0.826	0.009
SM(d42:3)	0.041	−0.494	0.140	0.205	0.296	−2.409	0.768	0.018
SM(d43:1)	0.106	−0.500	0.147	0.215	0.721	−2.328	0.473	0.023
SM(d43:2)	0.167	−0.606	0.140	0.204	1.200	−2.966	0.234	0.004
SM(d44:4)	0.019	−0.421	0.136	0.200	0.143	−2.107	0.887	0.038
SM(d44:5)	0.134	−0.575	0.131	0.192	1.028	−3.001	0.308	0.004
SM(d44:6)	0.054	−0.427	0.139	0.204	0.389	−2.092	0.699	0.040
SM(t18:0_24:2)	0.163	−0.509	0.143	0.209	1.145	−2.436	0.256	0.017
SM(t42:1)	0.240	−0.578	0.139	0.204	1.724	−2.832	0.089	0.006
TG(12:0_17:1_18:2)	−0.122	0.473	0.144	0.211	−0.848	2.243	0.399	0.028
TG(12:0_18:2_18:2)	−0.159	0.555	0.134	0.196	−1.191	2.832	0.237	0.006
TG(14:0_14:3_18:2)	−0.136	0.470	0.143	0.209	−0.950	2.250	0.345	0.027
TG(14:0_18:3_18:3)	−0.046	0.518	0.135	0.197	−0.345	2.623	0.731	0.011
TG(15:0_14:1_16:1)	−0.048	0.463	0.129	0.189	−0.375	2.453	0.709	0.017
TG(15:0_16:0_20:5)	−0.063	0.480	0.133	0.195	−0.474	2.464	0.637	0.016
TG(16:0_14:0_18:3)	−0.142	0.468	0.138	0.202	−1.032	2.317	0.305	0.023
TG(16:0_14:1_16:1)	−0.198	0.579	0.146	0.213	−1.360	2.711	0.178	0.008
TG(16:1_12:0_18:2)	−0.046	0.421	0.142	0.208	−0.324	2.023	0.747	0.047
TG(16:1_20:5_20:5)	0.088	0.503	0.126	0.185	0.696	2.724	0.488	0.008
TG(18:3_14:1_18:2)	−0.045	0.412	0.141	0.206	−0.319	1.999	0.751	0.049
TG(18:4_14:0_16:1)	−0.155	0.616	0.143	0.209	−1.082	2.944	0.283	0.004
TG(18:4_16:0_20:4)	−0.081	0.525	0.141	0.207	−0.571	2.533	0.570	0.013
TG(20:1_20:4_20:4)	0.304	−0.531	0.144	0.210	2.117	−2.524	0.038	0.014
TG(22:5_18:2_18:2)	−0.078	0.454	0.147	0.215	−0.529	2.110	0.598	0.038
TG(33:4e)	−0.160	0.651	0.137	0.201	−1.168	3.241	0.247	0.002

Marginal effect: Beta.PRS/SE.PRS/t.PRS/Pval. PRS: Beta coefficient/standard error/t statistics/*p* value of association between PRS scores and individual lipids.

Interaction effect: Beta.PRS_CC/SE.PRS_CC/t.PRS_CC/Pval.PRS_CC: Beta coefficient/standard error/t statistics/*p* value of differential association of PRS with lipids between AD and controls control.

*Cer* ceramides, *SM* sphingomyelins, *ChE* cholesteryl esters, *DG* diglycerides, *TG* triglycerides, *PC* phosphatidylcholines, *LPC* lyso-phosphatidylcholines, *PE* phosphatidylethanolamines, *PI* phosphatidylinositols.

**Fig. 6:**
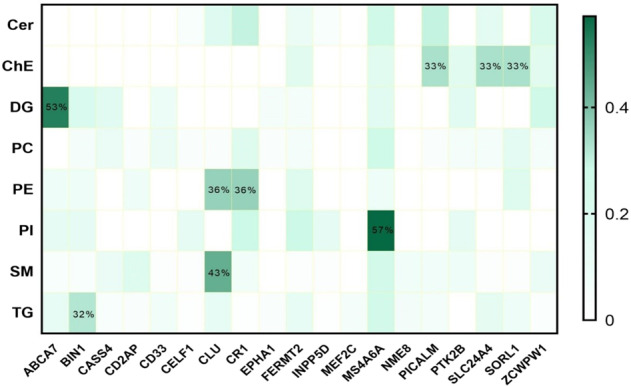
Proportion of lipids significantly differentially associated with AD-related genes. ABCA7 ATP binding cassette subfamily A member 7, BIN1 bridging integrator 1, CASS4 Crk associated substrate 4, CD2AP CD2 associated protein, CD33 sialic acid binding Ig-like lectin 3, CUGBP elav-like family member 1, CLU clusterin, CR1 complement C3b/C4b receptor 1, EPHA1 EPH receptor A1, FERMT2 FERM domain containing kindlin 2, INPP5D inositol polyphosphate-5-phosphatase D, MEF2C myocyte enhancer factor 2C, MS4A6A membrane spanning 4-domains A6A, NME8 NME/NM23 family member 8, PICALM phosphatidylinositol binding clathrin assembly protein, PTK2B protein tyrosine kinase 2 beta, SLC24A4 solute carrier family 24 member 4, SORL1 sortilin related receptor 1, ZCWPW1 zinc finger CW-type and PWWP domain containing 1.

We further explored the association of AD-associated genes (*n* = 19) derived from the list of 33 SNPs with lipids in each lipid class (Table 4). *FERMT2* and *MS4A6A* showed a significantly differential association with lipids in all lipid classes across disease and control groups. In addition, PC, SM, and TG lipids were differentially associated with the largest number of AD-related genes. The heatmap in Fig. 6 shows the proportion of lipids in each lipid class having differential associations with the AD-associated genes. *ABCA7* had a differential association with more than half of the DG lipids (52.63%) and PI lipids (57.14%), respectively. Additionally, 43.4% of lipids in the SM class were differentially associated with *CLU*. More than 30% of lipids in ChE, PE, and TG classes had differential associations with separate genes (ChE-*PICALM, SLC24A4*, and *SORL1*; PE-*CLU* and *CR1*; TG-*BINI*) between AD and control group. Finally, we found 43 lipids that were significantly associated with both the AD-related genes and PRS (Table 5).

**Table 4:** Differential interaction of genes with lipids between AD and control.

Genes	Cer	ChE	DG	PC	PE	PI	SM	TG
*ABCA7*			✓		✓	✓	✓	✓
*BIN1*			✓	✓	✓	✓	✓	✓
*CASS4*			✓	✓			✓	✓
*CD2AP*				✓	✓		✓	✓
*CD33*			✓	✓			✓	✓
*CELF1*	✓			✓		✓	✓	✓
*CLU*	✓			✓	✓		✓	✓
*CR1*	✓			✓	✓	✓	✓	✓
*EPHA1*			✓	✓				✓
*FERMT2*	✓	✓	✓	✓	✓	✓	✓	✓
*INPP5D*	✓					✓	✓	✓
*MEF2C*								✓
*MS4A6A*	✓	✓	✓	✓	✓	✓	✓	✓
*NME8*							✓	✓
*PICALM*	✓	✓		✓			✓	✓
*PTK2B*		✓	✓	✓		✓	✓	
*SLC24A4*	✓	✓		✓			✓	✓
*SORL1*		✓		✓	✓		✓	✓
*ZCWPW1*	✓	✓	✓	✓			✓	✓

“✓”: ≥1 lipid in each lipid class were differentially associated with the named gene between AD and controls.

*Cer* ceramides, *SM* sphingomyelins, *ChE* cholesteryl esters, *DG* diglycerides, *TG* triglycerides, *PC* phosphatidylcholines, *LPC* lyso-phosphatidylcholines, *PE* phosphatidylethanolamines, *PI* phosphatidylinositols.

**Table 5:** Overlap of lipids with significant association with SNPs and PRS scores.

Lipid	Number of associated SNPs	Most significant Pval.SNPs	Gene (minimum Pvalue.SNPs)	Pval.PRS_CC
Cer(d18:1_16:0)	3	0.009	*CLU*	0.011
ChE(22:5)	5	0.014	*SORL1*	0.009
PC(16:0_20:4)	1	0.045	*CASS4*	0.022
PC(16:0_20:5)	2	0.033	*SORL1*	0.013
PC(18:0_20:3)	1	0.027	*MS4A6A*	0.032
PC(18:1_20:3)	1	0.042	*CELF1*	0.022
PC(18:2_18:2)	1	0.007	*FERMT2*	0.044
PE(16:0_20:3)	1	0.005	*CR1*	0.038
PI(36:3)	1	0.002	*INPP5D*	0.007
SM(d17:1_13:0)	2	0.014	*SORL1*	0.002
SM(d17:1_18:3)	3	0.040	*CLU*	0.043
SM(d32:4)	3	0.008	*MS4A6A*	0.004
SM(d33:1)	5	0.021	*CLU*	0.013
SM(d34:2)	2	0.045	*CLU*	0.048
SM(d35:1)	1	0.012	*MS4A6A*	0.036
SM(d37:2)	4	0.014	*CD2AP*	0.034
SM(d38:1)	3	0.041	*CLU*	0.027
SM(d38:2)	3	0.022	*CLU*	0.025
SM(d39:1)	3	0.027	*CLU*	0.035
SM(d39:2)	3	0.027	*CLU*	0.013
SM(d40:1)	5	0.021	*CLU*	0.025
SM(d41:4)	1	0.028	*MS4A6A*	0.049
SM(d42:1)	2	0.013	*MS4A6A*	0.035
SM(d42:2)	5	0.012	*MS4A6A*	0.009
SM(d42:3)	3	0.044	*ABCA7*	0.018
SM(d43:1)	2	0.030	*CD2AP*	0.023
SM(d43:2)	1	0.032	*CASS4*	0.004
SM(d44:4)	3	0.016	*CLU*	0.038
SM(d44:5)	4	0.026	*CLU*	0.004
SM(t18:0_24:2)	3	0.022	*MS4A6A*	0.017
SM(t42:1)	5	0.023	*MS4A6A*	0.006
TG(12:0_17:1_18:2)	1	0.013	*NME8*	0.028
TG(14:0_14:3_18:2)	1	0.037	*BIN1*	0.027
TG(14:0_18:3_18:3)	3	0.003	*NME8*	0.011
TG(15:0_14:1_16:1)	1	0.032	*NME8*	0.017
TG(15:0_16:0_20:5)	2	0.048	*BIN1*	0.016
TG(16:0_14:0_18:3)	5	0.011	*CLU*	0.023
TG(16:0_14:1_16:1)	1	0.018	*SORL1*	0.008
TG(18:3_14:1_18:2)	2	0.035	*ABCA7*	0.049
TG(18:4_14:0_16:1)	1	0.045	*ZCWPW1*	0.004
TG(18:4_16:0_20:4)	3	0.011	*CLU*	0.013
TG(22:5_18:2_18:2)	4	0.017	*NME8*	0.038
TG(33:4e)	4	0.011	*MS4A6A*	0.002

Only lipids with significant differential associations with both PRS and SNPs were included in the table. The most significant *p* value of lipids association with SNPs of the same gene was presented when the SNP had the most significant differential association with the lipid across AD and control.

## Discussion

In this study, significant differences in plasma lipids between AD cases and controls were observed. In vitro analyses demonstrated similar changes in astroglial cells when treated with *Aβ*_*42*_. GLMnet algorithm was used to distinguish AD from controls using different plasma lipid-based models. SMs, ChEs, and TGs showed the greatest accuracy in discriminant analysis with AUC of more than 80%. Finally, associations between AD case-control status and genetic risk factors for AD were nominally significant when examining lipid profiles.

### Identification of the roles of lipids associated with AD

Our study did not show a significant group ceramide difference between AD and controls. However, we found that most individual ceramides differed significantly between these two groups. Molecular profiles of the ceramidome showed that dihydroxy Cer species, comprising fatty acyls (18C) with 0 or 2 double bonds and all dihydroxy Cers with 19C, were significantly increased in AD. On the other side, dihydroxy Cers containing 16C or 18C with 1 double bond showed an opposite result. Most monohydroxy and trihydroxy Cers decreased significantly in AD subjects, which might suggest the opposite pathobiology of different Cer subgroups. Ceramides are essential for the maintenance of membrane structure stabilization, cell-to-cell recognition, and secondary messenger signaling^27^. Dysregulation of Cers has been linked to cognitive decline and brain atrophy^28^. Higher levels of serum ceramide were associated with increased risk of AD compared to subjects with the lowest tertile of serum ceramide levels in another study^29^. In addition, even though the sum of SM lipids was not significantly different between the two groups, we found that most individual SM lipids were lower in AD plasma. Altered sphingolipid metabolism has been associated with the pathogenesis of several neurodegenerative diseases including AD, and metabolic syndrome^29–32^. The decrease in SM lipids has been shown to be strongly correlated with parameters of insulin resistance, and lipid metabolism, which are major risk factors for AD^33^. A cross-sectional study (*n* = 26 NC, 26 AD) observed reduced SM species in AD versus controls and long aliphatic chains (C22, C24) in particular ^31^, which is consistent with our results.

We did not find any significant differences among group PC lipids. However, the AD population tended to present higher abundance in individual PC lipids comprising very short and short-chain fatty acyls except for PC containing C15 without double bonds and C16 with 2 double bonds. Recent studies have demonstrated altered PC metabolism in cognitively impaired elderly and AD patients^9,34,35^. Similar to PC lipids, we did not observe significant changes in group PE. About 24% of individual PEs were all lower in AD patients, which is almost 2 fold of PEs that were higher in AD. PE has been previously reported to be significantly reduced in the brains of individuals with AD and HD^36^. Since a multitude of neuropathological processes can lead to a decrease in PC and PE, it is likely that reduced levels of these phospholipids may be linked to the observed neuronal loss. Increased activities of the Kennedy pathway enzymes, phosphoethanolamine cytidylyltransferase, phosphocholine cytidylyltransferase, and PS synthase have been previously reported to be elevated in the diseased brain regions of patients with AD and PD^34^. Contrary to the decreased trend of other phospholipids, including PC and PE, we observed an opposite change in PI in AD patients, with most of the individual PI lipids higher in AD subjects. PI is another phospholipid that is present in the membrane of almost all cell types and is involved in mediating Ca^2+^ mobilization in response to many hormones, neurotransmitters, and growth factors^37^. Consistently, an animal study showed that lower levels of plasma PC and higher plasma PI were correlated with post-stroke cognitive impairment^38^.

We also found significant differences in ChEs between AD and controls. ChE is produced in the plasma by the conversion of fatty acids to cholesterol from PC by the enzymatic activity of cholesterol acyl transferase (LCAT)^9,39^. While free cholesterol can be taken up by APOE containing liposomes (e.g. HDL) and is bound to the outer particle surface, esterification enhances cholesterol uptake within the interior of the lipoproteins and enhances cholesterol transport through the bloodstream. LCAT has a preference for highly unsaturated fatty acid chain PC and can link the reduction in PC to dysregulation of specific steps in cholesterol metabolism in AD^40^. Another enzyme, acyl-coenzyme A can also esterify cholesterol in other tissues. Inhibition of this enzyme has been reported to reduce amyloid plaque load in the brain of AD mice and improves cognitive outcomes^41^. Consistent with our results showing a significant increase of DGs in Alzheimer’s disease, previous studies have demonstrated augmented levels of DGs in the frontal cortex and plasma of AD patients^42^. DGs are important in maintaining structural integrity and signal transduction^42^ The conversion of DGs to phosphatidic acid by DG kinase has been reported to be decreased in the AD brain^43^. We also found almost 2/3 plasma TG lipids in all significant TG lipids were higher in AD subjects, even though the abundance of group TGs were not different among disease groups. A longitudinal finding was reported in the Honolulu-Asia Aging Study in which a 1SD increase in TG levels during midlife significantly increased the risk of dementia a quarter of a decade later^44^. The exploration of the exact pathobiology of individual TG lipids is needed in the future.

### Relationship between plasma lipid profiles and the cell lipidome exposed to oligomeric Aβ_42_ in vivo

Several studies have reported significant differences in lipid profiles between plasma and postmortem brain tissue^45^. The separation of the brain from the periphery is a major challenge when examining the biological significance of plasma lipids in Alzheimer’s disease since not all lipids can be transported across the blood–brain barrier. This separation may likely be compromised by age-related changes in blood–brain barrier integrity, affecting lipid and Aβ kinetics. To determine whether our observed changes in the plasma lipidome were related to AD pathology we also quantified the cellular lipidome in human U251 astroglioma cell lines exposed to pathophysiological concentrations of oligomeric Aβ_42_.

Astrocytes are important cells in the CNS and are involved in various physiological aspects^46^. Neurons are thought to be the main source of Aβ in the adult brain. Moreover, the ability of neurons to generate cholesterol in the adult brain is impaired during development. However, astrocytes can produce cholesterol and transport it to neurons with apolipoprotein E (apoE)^47^. ApoE is the major genetic risk factor for sporadic AD. This has led to the hypothesis that apoE regulates Aβ formation via modulation of lipid raft functions^48^. Recently, astrocyte-derived cholesterol has been shown to regulate Aβ formation in vivo and influence processes involved in inflammation and neurodegeneration^49^. In our study, we quantified the sum of lipid classes when the astroglioma cell line was exposed to subpathological amounts of oligomeric Aβ_42_. We still found a similar trend of lipid changes between plasma and cells treated with oligomeric Aβ_42_. For example, SM, PI, and TG were significantly higher oligomeric Aβ_42_-treated cells, and some SM, PI, and TG species were upregulated in AD plasma. However, no significant differences were observed between AD and control in plasma and cells for other lipid groups. The results in the cellular lipidome support findings that Aβ_42_ plays a contributory if not causal role in mediating changes in plasma and cellular lipid profiles in AD.

### The lipidome signature as a biomarker of AD

We also examined whether a battery of plasma lipids can be used to discriminate AD patients from controls. All individual lipids, 9 subgroup lipids, and group lipids were used in the GLMnet algorithm. We observed that three classification models, using either TG, ChE, and SM, had significant power in discriminating AD from controls, with >80% AUC. Additionally, individual lipids in TG, ChE, and SM showed even higher AUC than group lipids. Therefore, measurement of these group lipids using mass spectrometry will provide renewed insight in the underlying mechanisms of AD and provide additional drug targets. Taken together, our findings suggest that alterations in both cholesterol and sphingolipid metabolism may play an important role in the pathobiology of AD.

### AD genetic risk, lipids, and AD

We examined the relationships between AD PRS and the AD-associated lipids in our sample. Even though no significant association was found after FDR correction, we observed a number of lipid species that were nominally differentially affected between AD and controls, most of which were PC, SM, and TGs. Our data suggest that these lipids might mediate the effect of several SNPs linked to AD.

We also investigated individual AD-related SNPs and lipid levels. Significant associations between AD-related SNPs and individual lipids were only observed before FDR correction. However, we found all SNPs having nominally significant differential association with a certain number of lipid species between AD and control group.

*FERMT2* and *MS4A6A* genes showed a significantly differential association with lipids in all lipid classes across disease groups. *FERMT2* is a member of the Fermitin family of proteins, which are involved in cell–matrix adhesion complexes. Shulman et al.^50^ validated the association of *FERMT2* with AD risk after performing a gene screen and in vivo studies in *Drosophila melanogaster*. Their work showed altered expression of both *FERMT2* and *CELF1* homologs modulates tau neurotoxicity. It is also upregulated in atherosclerotic plaques, which is a risk factor for AD^51^. The correlation of *FERMT2* with post-stroke brain recovery^52^ and higher plasma PI in post-stroke mice^38^ supports the relationship between *FERMT2* and PI in our study, i.e. the PI class had the highest proportion of lipids of differential association with *FERMT2* between AD and control. It has been hypothesized that FERMT2 regulates APP internalization and degradation^53^. For instance, FERMT2 silencing induces increased amounts of full-length APP and by-products and FERMT2 over-expression leads to a reduction of APP and its related metabolites. Recently, a direct interaction between FERMT2 and APP—through the F3 domain of FERMT2 and the NxTY motif within APP’s intracellular domain was reported^54^ and can influence axonal growth^55^. Importantly, because amyloidogenic processing of APP is dependent on lipid rafts, we postulate that the effect of FERMT2 might be—at least in part—due to alterations in lipid profiles which alter the accumulation of full-length APP or its by-products within the growth cone, impairing vesicle trafficking and/or cell adhesion.

*MS4A6A* is membrane-spanning 4-domains, subfamily A, member 6A having been revealed to be associated with cortical and hippocampal atrophy independent of glucose metabolism and Aβ deposition^56^. The important association between *MS4A6A* and PI (57.14% had differential association with *MS4A6A*) might indicate the involvement of PI mediating *MS4A6A*’s effect on brain atrophy. Another gene *ABCA7* had an important effect on more than 50% of lipids in the DG lipid class, but with little influence on other lipid classes. DG metabolism and cholesterol synthesis might be the key component linking *ABCA7* to Alzheimer’s onset. Increasing evidence has demonstrated that ABCA7 deficiency exacerbates Aβ pathology in vitro and in vivo models. In detail, ABCA7 is involved in the microglial Aβ clearance pathway and accelerated Aβ production^57^. ABCA7 may also mediate the release of cellular cholesterol and phospholipids to generate HDL. Human ABCA7 mediates the efflux of both cellular cholesterol and phospholipids to apoA-I, whereas mouse ABCA7 mediates only phospholipids but not cholesterol efflux to apoA-I^58^. Both *MS4A6A* and *ABCA7* have been previously linked to the immune and complement system^59^, lipid metabolism, and immune system^60^, immune response and inflammation^61^, cytokine signaling in immune system ^62^, cholesterol/lipid metabolism, and immune and complement systems^63^.

*CLU-*encoded apoliprotein J associated with the clearance of cellular debris and apoptosis, which was suggested to be involved in Abeta-independent pathways as part of the cascade leading to Alzheimer pathology^64^. In our study, SM and PE were revealed to be associated with *CLU* in the pathogenesis of AD. ChE lipids were differentially associated with *PICALM, SLC24A4*, and *SORL1* between AD and control*. PICALM* has been associated with reduced connectivity of the frontal gyrus with the hippocampus, as well as with the precuneus. The *SLC24A4* gene encodes the 24 solute carrier family member 4 protein, which is a member of the K^+^-dependent Na^+^/Ca^2+^ exchanger protein family. These exchanger proteins are widely expressed in brain tissues, suggesting that SLC24A4 may play an important role in the nervous system. The association of this gene with blood pressure in African Americans suggests it may have relevance with AD through vascular disease^65^. *SORL1* belongs to the low-density lipoprotein receptor family, binding ApoE and lipoprotein lipase on membranes^66^. Decreased Sorl1 has been identified as related to AD^67^. It is downregulated in lymphoblasts and cortical pyramidal neurons of AD patients. The mechanisms may include APP trafficking and recycling^68^, Additionally, the association of SORL1 with risk of amnestic mild cognitive impairment (aMCI) has been reported in the Han Chinese^69^. TG lipids were correlated with *BIN*, which functions in clathrin-mediated endocytosis and endocytic recycling, as does the AD risk gene *PICALM*. DNA methylation of the BIN1 promoter has been suggested as a possible epigenetic mechanism influencing AD risk.

Several prior studies have been conducted to explore the potential pathological roles of AD-related SNPs. A review of GWAS-identified risk genes for AD found they were clustered into three main molecular pathways: lipid metabolism, immune function, and endosome vesicle cycling^70^. The lipids that have shown marked differences between AD versus controls which been directly and indirectly linked to at least one of these mechanisms. The significant associations between lipid profiles and AD which were observed in our study encouraged us to further explore the possible mechanisms of lipids changes in AD patients.

Our results highlight that genetic pathway and SNPs related to AD influence lipids that are associated with AD. These results reinforce the importance of lipid metabolism and dysregulation in AD. It also suggests inflammation, ion channel modification, and Aβ pathways influence lipid levels that are dysregulated in AD. While studies examining the exact mechanisms linking these SNPs with reported lipid changes in AD patients are nascent in the current literature, our results provide renewed insights on potential lipid-related mechanisms in AD which may be examined further in preclinical and clinical studies.

### Limitations

Firstly, the sample size was around 40 each in the AD and paired control group, which provides enough power for analyzing 9 lipid classes. However, the strength of the data is limited for examining a large number of individual lipids. FDR correction was used to reduce the bias, although a larger cohort is needed to replicate the findings.

Secondly, we did not subclassify AD to different stages. In addition, we did not look at overlapping cerebral vascular disease in AD patients because only 10 of 40 AD subjects had MRI scanning. Therefore, future studies will look at lipid differences between control, pure AD, and mixed AD in the same stage.

Thirdly, our study is useful for exploring clinical biomarkers of AD because plasma lipids are relatively easy to extract. However, plasma lipids may also be affected by damage to peripheral organs as well, which limits the accuracy of any association between changes in plasma lipid levels and brain pathology. The lipidomics of CSF or post-mortem brain samples from AD patients will be necessary to validate our findings in the future.

## Conclusion

Our findings suggest that plasma lipids may differentiate between AD and cognitively normal controls and lipids models can be applied to discriminate the two groups. In addition, AD-related SNPs may have a different association with lipids between AD and controls and the underlying mechanisms need to be explored further in the future. In summary, our study provides evidence that certain lipids may be involved in AD pathogenesis and are associated with AD-related SNPs.

## Supplementary information

Supplementary table 1. Variants of of AD risk genes included in analysis

Supplementary table 2. Fold change of original abundance of lipids and difference of lipid residuals between AD and control

Supplementary table 3. Comparison of residuals of group lipids in AD and Control group

Supplementary table 4. Differential association of the AD risk PRS scores with lipids between AD and controls

Supplementary table 5. Significantly differential association of the AD risk variants and the SNPs with lipids between AD and controls
