# Supplementary material for: Plasma lipidome is dysregulated in Alzheimer’s disease and is associated with disease risk genes
Source: Transl Psychiatry. 2021 Jun 7;11:344. doi: 10.1038/s41398-021-01362-2 (PMC8180517; doi:10.1038/s41398-021-01362-2)
Supplement: Supplementary file 1 — Supplementary table 1. Variants of of AD risk genes included in analysis [file 41398_2021_1362_MOESM1_ESM.docx]

Supplementary table 1. Variants of of AD risk genes included in analysis

| **Gene** | **SNP** | **Chromosome** | **Position** | **Minor Allele** | **MAF** | **Imputation quality** | **Genotyped** |
| --- | --- | --- | --- | --- | --- | --- | --- |
| *CR1* | rs6656401 | 1 | 207692049 | A | 0.18004 | 1.00028 | Genotyped |
| *CR1* | rs3818361 | 1 | 207784968 | A | 0.18809 | 0.99634 | Imputed |
| *CR1* | rs6701713 | 1 | 207786289 | A | 0.18794 | 0.99728 | Imputed |
| *CR1* | rs12034383 | 1 | 207803595 | G | 0.40166 | 0.99951 | Imputed |
| *BIN1* | rs7561528 | 2 | 127889637 | A | 0.33443 | 0.85384 | Imputed |
| *BIN1* | rs6733839 | 2 | 127892810 | T | 0.36753 | 0.64097 | Imputed |
| *BIN1* | rs744373 | 2 | 127894615 | G | 0.26929 | 0.67836 | Imputed |
| *INPP5D* | rs35349669 | 2 | 234068476 | T | 0.46992 | 0.83336 | Imputed |
| *MEF2C* | rs190982 | 5 | 88223420 | G | 0.39686 | 0.89843 | Imputed |
| *CD2AP* | rs9349407 | 6 | 47453378 | C | 0.26984 | 0.99987 | Imputed |
| *CD2AP* | rs10948363 | 6 | 47487762 | G | 0.27289 | 0.99076 | Imputed |
| *NME8* | rs2718058 | 7 | 37841534 | G | 0.37436 | 0.95086 | Imputed |
| *ZCWPW1* | rs1476679 | 7 | 100004446 | C | 0.31123 | 0.99518 | Genotyped |
| *EPHA1* | rs11767557 | 7 | 143109139 | C | 0.18762 | 0.99589 | Genotyped |
| *EPHA1* | rs11771145 | 7 | 143110762 | A | 0.31222 | 0.79223 | Imputed |
| *PTK2B* | rs28834970 | 8 | 27195121 | C | 0.35459 | 0.9836 | Imputed |
| *CLU* | rs11136000 | 8 | 27464519 | T | 0.40107 | 0.99977 | Genotyped |
| *CLU* | rs1532278 | 8 | 27466315 | T | 0.3986 | 0.98024 | Imputed |
| *CLU* | rs9331896 | 8 | 27467686 | C | 0.40284 | 0.96147 | Imputed |
| *CELF1* | rs10838725 | 11 | 47557871 | C | 0.31304 | 0.79772 | Imputed |
| *MS4A6A* | rs983392 | 11 | 59923508 | G | 0.40198 | 0.99313 | Imputed |
| *MS4A6A* | rs610932 | 11 | 59939307 | T | 0.43607 | 0.98512 | Imputed |
| *PICALM* | rs561655 | 11 | 85800279 | G | 0.34353 | 0.9999 | Genotyped |
| *PICALM* | rs10792832 | 11 | 85867875 | A | 0.37491 | 0.99651 | Imputed |
| *PICALM* | rs3851179 | 11 | 85868640 | T | 0.37465 | 0.99677 | Genotyped |
| *SORL1* | rs11218343 | 11 | 121435587 | C | 0.04466 | 0.99743 | Genotyped |
| *SORL1* | rs1131497 | 11 | 121502745 | G | 0.43181 | 0.91474 | Imputed |
| *FERMT2* | rs17125944 | 14 | 53400629 | C | 0.08217 | 1.00044 | Genotyped |
| *SLC24A4* | rs10498633 | 14 | 92926952 | T | 0.24147 | 0.99886 | Genotyped |
| *ABCA7* | rs3752246 | 19 | 1056492 | G | 0.20324 | 0.75676 | Imputed |
| *ABCA7* | rs4147929 | 19 | 1063443 | A | 0.20323 | 0.90575 | Imputed |
| *CD33* | rs3865444 | 19 | 51727962 | A | 0.27724 | 0.98835 | Imputed |
| *CASS4* | rs7274581 | 20 | 55018260 | C | 0.09316 | 0.96957 | Imputed |

MAF: minor allele frequency
