# Supplementary material for: Plasma lipidome is dysregulated in Alzheimer’s disease and is associated with disease risk genes
Source: Transl Psychiatry. 2021 Jun 7;11:344. doi: 10.1038/s41398-021-01362-2 (PMC8180517; doi:10.1038/s41398-021-01362-2)
Supplement: Supplementary file 2 — Supplementary table 2. Fold change of original abundance of lipids and difference of lipid residuals between AD and control [file 41398_2021_1362_MOESM2_ESM.docx]

**Supplementary table 2. Fold change of original abundance of lipids and difference of lipid residuals between AD and control**

| **Individual lipids** | **Mean_Control (residuals)** | **Mean_AD (residuals)** | **Fold change (AD/control)** | **t statistics** | **p value** | **Adjusted p value** |
| --- | --- | --- | --- | --- | --- | --- |
| Cer(d16:0_24:1) | -0.245 | 0.257 | 2.289 | -2.352 | 2.14E-02 | 4.17E-02 |
| Cer(d16:1_16:0) | -0.285 | 0.3 | 2.745 | -2.773 | 7.06E-03 | 1.63E-02 |
| Cer(d16:1_22:0) | 0.495 | -0.519 | 0.261 | 5.403 | 6.58E-07 | 1.40E-05 |
| Cer(d16:1_23:0) | 0.419 | -0.44 | 0.276 | 4.366 | 3.76E-05 | 2.43E-04 |
| Cer(d18:0_16:0) | -0.366 | 0.385 | 4.377 | -3.658 | 5.28E-04 | 1.86E-03 |
| Cer(d18:0_18:0) | -0.292 | 0.307 | 3.435 | -2.827 | 6.45E-03 | 1.51E-02 |
| Cer(d18:0_22:0) | -0.37 | 0.388 | 2.295 | -3.72 | 3.87E-04 | 1.44E-03 |
| Cer(d18:0_23:0) | -0.443 | 0.465 | 4.262 | -4.633 | 1.60E-05 | 1.36E-04 |
| Cer(d18:0_24:0) | -0.382 | 0.401 | 2.501 | -3.869 | 2.36E-04 | 9.46E-04 |
| Cer(d18:0_24:1) | -0.351 | 0.369 | 3.901 | -3.486 | 8.78E-04 | 2.91E-03 |
| Cer(d18:0_24:2) | -0.217 | 0.228 | 4.395 | -2.048 | 4.54E-02 | 7.77E-02 |
| Cer(d18:1_16:0) | 0.483 | -0.507 | 0.315 | 5.22 | 1.38E-06 | 2.46E-05 |
| Cer(d18:1_18:0) | 0.472 | -0.496 | 0.289 | 5.071 | 2.52E-06 | 3.37E-05 |
| Cer(d18:1_22:0) | 0.522 | -0.548 | 0.306 | 5.817 | 1.18E-07 | 4.89E-06 |
| Cer(d18:1_23:0) | 0.528 | -0.555 | 0.203 | 5.93 | 7.37E-08 | 3.80E-06 |
| Cer(d18:1_24:0) | 0.474 | -0.498 | 0.339 | 5.111 | 2.14E-06 | 3.02E-05 |
| Cer(d18:1_24:1) | 0.203 | -0.213 | 0.573 | 1.954 | 5.44E-02 | 9.06E-02 |
| Cer(d18:1_25:0) | -0.362 | 0.38 | 2.362 | -3.648 | 4.72E-04 | 1.70E-03 |
| Cer(d18:1_26:1) | 0.452 | -0.474 | 0.443 | 4.785 | 7.70E-06 | 7.59E-05 |
| Cer(d18:2_22:0) | 0.067 | -0.07 | 1.118 | 0.626 | 5.33E-01 | 6.06E-01 |
| Cer(d18:2_23:0) | 0.186 | -0.195 | 0.902 | 1.778 | 7.93E-02 | 1.25E-01 |
| Cer(d18:2_24:0) | -0.456 | 0.478 | 3.834 | -4.794 | 9.20E-06 | 8.85E-05 |
| Cer(d18:2_24:1) | -0.414 | 0.434 | 3.337 | -4.238 | 7.32E-05 | 3.85E-04 |
| Cer(d18:2_25:0) | -0.445 | 0.467 | 4.036 | -4.645 | 1.64E-05 | 1.38E-04 |
| Cer(d19:0_23:0) | -0.398 | 0.418 | 2.351 | -4.074 | 1.10E-04 | 5.30E-04 |
| Cer(d19:0_24:0) | 0.055 | -0.058 | 0.955 | 0.522 | 6.03E-01 | 6.71E-01 |
| Cer(d19:1_22:0) | 0.452 | -0.474 | 0.245 | 4.8 | 7.33E-06 | 7.42E-05 |
| Cer(d19:1_24:0) | -0.427 | 0.449 | 4.372 | -4.409 | 4.14E-05 | 2.63E-04 |
| Cer(d19:1_24:1) | -0.315 | 0.331 | 2.484 | -3.084 | 2.97E-03 | 8.21E-03 |
| Cer(d38:1) | -0.398 | 0.418 | 3.16 | -4.057 | 1.26E-04 | 5.91E-04 |
| Cer(m18:0_20:0) | 0.431 | -0.453 | 0.277 | 4.522 | 2.11E-05 | 1.63E-04 |
| Cer(m18:0_22:0) | 0.475 | -0.499 | 0.238 | 5.114 | 2.11E-06 | 3.02E-05 |
| Cer(m18:0_23:0) | -0.027 | 0.028 | 1.294 | -0.25 | 8.03E-01 | 8.50E-01 |
| Cer(m18:0_24:0) | 0.377 | -0.396 | 0.528 | 3.838 | 2.47E-04 | 9.85E-04 |
| Cer(m18:0_24:1) | 0.435 | -0.457 | 0.293 | 4.578 | 1.70E-05 | 1.40E-04 |
| Cer(m18:1_20:0) | 0.507 | -0.533 | 0.212 | 5.584 | 3.15E-07 | 9.93E-06 |
| Cer(m18:1_22:0) | -0.331 | 0.348 | 3.195 | -3.258 | 1.80E-03 | 5.41E-03 |
| Cer(m18:1_23:0) | 0.397 | -0.417 | 0.352 | 4.089 | 1.03E-04 | 5.10E-04 |
| Cer(m18:1_24:0) | 0.303 | -0.318 | 0.525 | 3 | 3.62E-03 | 9.61E-03 |
| Cer(m18:1_24:1) | 0.454 | -0.477 | 0.294 | 4.83 | 6.46E-06 | 7.06E-05 |
| Cer(t16:1_12:0) | -0.147 | 0.155 | 2.299 | -1.379 | 1.73E-01 | 2.40E-01 |
| Cer(t16:1_14:0) | 0.383 | -0.402 | 0.206 | 3.939 | 1.88E-04 | 7.83E-04 |
| Cer(t16:1_16:0) | 0.148 | -0.156 | 0.322 | 1.416 | 1.61E-01 | 2.26E-01 |
| ChE(16:0) | -0.557 | 0.585 | 3.335 | -6.368 | 1.32E-08 | 1.49E-06 |
| ChE(17:0) | 0.426 | -0.447 | 0.305 | 4.46 | 2.71E-05 | 1.93E-04 |
| ChE(18:0) | 0.318 | -0.334 | 0.607 | 3.149 | 2.30E-03 | 6.56E-03 |
| ChE(18:2) | 0.346 | -0.363 | 0.457 | 3.457 | 8.88E-04 | 2.92E-03 |
| ChE(18:3) | 0.533 | -0.56 | 0.025 | 6.02 | 5.14E-08 | 3.55E-06 |
| ChE(20:1) | -0.335 | 0.352 | 1.824 | -3.306 | 1.52E-03 | 4.63E-03 |
| ChE(20:2) | 0.384 | -0.403 | 0.432 | 3.883 | 2.25E-04 | 9.16E-04 |
| ChE(20:3) | 0.497 | -0.522 | 0.186 | 5.439 | 5.71E-07 | 1.32E-05 |
| ChE(20:4) | -0.101 | 0.106 | 2.148 | -0.933 | 3.55E-01 | 4.33E-01 |
| ChE(22:3) | -0.479 | 0.503 | 4.393 | -5.146 | 2.06E-06 | 3.01E-05 |
| ChE(22:4) | 0.476 | -0.5 | 0.314 | 5.139 | 1.93E-06 | 2.99E-05 |
| ChE(22:5) | -0.295 | 0.31 | 2.301 | -2.876 | 5.33E-03 | 1.28E-02 |
| ChE(22:6) | -0.391 | 0.411 | 3.856 | -3.972 | 1.70E-04 | 7.28E-04 |
| ChE(23:1) | 0.466 | -0.489 | 0.42 | 4.922 | 6.62E-06 | 7.06E-05 |
| DG(13:0) | -0.124 | 0.13 | 5.258 | -1.153 | 2.53E-01 | 3.27E-01 |
| DG(16:0_16:0) | 0.24 | -0.252 | 0.347 | 2.32 | 2.29E-02 | 4.40E-02 |
| DG(16:0_16:1) | -0.297 | 0.312 | 2.86 | -2.887 | 5.23E-03 | 1.27E-02 |
| DG(16:0_18:1) | 0.391 | -0.41 | 0.227 | 3.996 | 1.43E-04 | 6.43E-04 |
| DG(16:0_18:2) | 0.214 | -0.225 | 0.207 | 2.064 | 4.24E-02 | 7.44E-02 |
| DG(16:0_18:3) | -0.436 | 0.458 | 4.321 | -4.526 | 2.51E-05 | 1.87E-04 |
| DG(16:0_20:4) | -0.403 | 0.423 | 3.122 | -4.125 | 9.66E-05 | 4.86E-04 |
| DG(16:0_22:6) | -0.184 | 0.193 | 1.292 | -1.746 | 8.48E-02 | 1.32E-01 |
| DG(16:1_18:1) | -0.347 | 0.364 | 2.341 | -3.459 | 8.92E-04 | 2.92E-03 |
| DG(16:1_18:2) | -0.285 | 0.299 | 2.499 | -2.759 | 7.45E-03 | 1.71E-02 |
| DG(17:0_18:1) | -0.389 | 0.408 | 3.2 | -3.949 | 1.78E-04 | 7.55E-04 |
| DG(17:1_18:1) | -0.429 | 0.451 | 4.115 | -4.454 | 2.96E-05 | 2.09E-04 |
| DG(18:0_16:0) | 0.154 | -0.161 | 0.297 | 1.47 | 1.46E-01 | 2.08E-01 |
| DG(18:0_18:0) | -0.423 | 0.444 | 3.431 | -4.395 | 3.38E-05 | 2.28E-04 |
| DG(18:0_18:1) | 0.382 | -0.401 | 0.235 | 3.904 | 1.99E-04 | 8.25E-04 |
| DG(18:1_14:0) | 0.308 | -0.324 | 0.439 | 3.033 | 3.29E-03 | 8.88E-03 |
| DG(18:1_18:1) | -0.415 | 0.436 | 2.695 | -4.274 | 5.73E-05 | 3.30E-04 |
| DG(18:1_18:3) | -0.312 | 0.328 | 1.543 | -3.084 | 2.81E-03 | 7.81E-03 |
| DG(18:1_20:3) | -0.414 | 0.435 | 3.658 | -4.26 | 6.05E-05 | 3.44E-04 |
| DG(18:1_20:4) | 0.294 | -0.308 | 0.206 | 2.898 | 4.88E-03 | 1.20E-02 |
| DG(18:1_20:5) | -0.097 | 0.101 | 0.573 | -0.914 | 3.64E-01 | 4.39E-01 |
| DG(18:1_22:5) | -0.321 | 0.337 | 2.931 | -3.165 | 2.25E-03 | 6.46E-03 |
| DG(18:1_22:6) | 0.076 | -0.08 | 0.379 | 0.718 | 4.75E-01 | 5.47E-01 |
| DG(18:2_18:2) | -0.291 | 0.305 | 3.321 | -2.836 | 5.86E-03 | 1.40E-02 |
| DG(18:3_18:2) | -0.087 | 0.091 | 0.649 | -0.816 | 4.17E-01 | 4.91E-01 |
| DG(19:1_6:0) | 0.214 | -0.224 | 0.216 | 2.063 | 4.26E-02 | 7.46E-02 |
| DG(20:0_18:1) | -0.489 | 0.513 | 3.197 | -5.291 | 1.08E-06 | 2.17E-05 |
| DG(20:0_18:2) | -0.463 | 0.486 | 4.052 | -4.917 | 4.88E-06 | 5.75E-05 |
| DG(20:1_18:2) | -0.417 | 0.438 | 3.307 | -4.295 | 5.30E-05 | 3.17E-04 |
| DG(20:5_18:2) | -0.342 | 0.359 | 3.255 | -3.398 | 1.10E-03 | 3.49E-03 |
| DG(22:4e) | -0.33 | 0.346 | 4.215 | -3.264 | 1.66E-03 | 5.03E-03 |
| DG(23:2) | 0.144 | -0.151 | 0.254 | 1.374 | 1.74E-01 | 2.41E-01 |
| DG(23:4e) | -0.132 | 0.139 | 2.469 | -1.231 | 2.23E-01 | 2.95E-01 |
| DG(25:1) | 0.002 | -0.002 | 0.45 | 0.017 | 9.87E-01 | 9.89E-01 |
| DG(29:1) | -0.098 | 0.102 | 0.756 | -0.917 | 3.62E-01 | 4.38E-01 |
| DG(30:2e) | -0.457 | 0.48 | 3.936 | -4.839 | 6.77E-06 | 7.12E-05 |
| DG(32:2e) | -0.47 | 0.494 | 3.282 | -5.044 | 2.80E-06 | 3.62E-05 |
| DG(32:3e) | 0.108 | -0.113 | 0.648 | 1.014 | 3.14E-01 | 3.93E-01 |
| DG(34:1e) | -0.496 | 0.521 | 3.316 | -5.414 | 6.44E-07 | 1.40E-05 |
| DG(34:2e) | -0.221 | 0.232 | 1.189 | -2.137 | 3.58E-02 | 6.39E-02 |
| DG(34:3e) | -0.181 | 0.19 | 1.269 | -1.713 | 9.07E-02 | 1.38E-01 |
| DG(34:4e) | -0.434 | 0.456 | 3.11 | -4.543 | 1.98E-05 | 1.56E-04 |
| DG(35:3e) | 0.247 | -0.26 | 0.489 | 2.384 | 1.95E-02 | 3.88E-02 |
| DG(36:1) | -0.488 | 0.512 | 3.903 | -5.267 | 1.30E-06 | 2.38E-05 |
| DG(36:3) | -0.484 | 0.508 | 3.489 | -5.211 | 1.59E-06 | 2.53E-05 |
| DG(36:3e) | -0.159 | 0.167 | 0.936 | -1.505 | 1.36E-01 | 1.97E-01 |
| DG(36:4e) | -0.486 | 0.51 | 4.028 | -5.25 | 1.27E-06 | 2.38E-05 |
| DG(36:5) | 0.182 | -0.192 | 0.316 | 1.743 | 8.53E-02 | 1.32E-01 |
| DG(36:5e) | -0.499 | 0.524 | 3.711 | -5.437 | 6.22E-07 | 1.40E-05 |
| DG(40:6) | 0.242 | -0.254 | 0.255 | 2.35 | 2.14E-02 | 4.17E-02 |
| LPC(14:0) | 0.095 | -0.1 | 1.456 | 0.883 | 3.80E-01 | 4.53E-01 |
| LPC(15:0) | 0.076 | -0.079 | 1.08 | 0.705 | 4.83E-01 | 5.54E-01 |
| LPC(16:0) | 0.119 | -0.125 | 1.029 | 1.109 | 2.71E-01 | 3.45E-01 |
| LPC(16:0e) | 0.012 | -0.012 | 1.78 | 0.108 | 9.14E-01 | 9.37E-01 |
| LPC(16:1) | 0.148 | -0.155 | 0.963 | 1.385 | 1.70E-01 | 2.38E-01 |
| LPC(16:1e) | 0.195 | -0.205 | 0.517 | 1.856 | 6.73E-02 | 1.08E-01 |
| LPC(17:0) | 0.016 | -0.017 | 1.802 | 0.15 | 8.81E-01 | 9.10E-01 |
| LPC(18:0) | -0.129 | 0.135 | 3.208 | -1.198 | 2.35E-01 | 3.11E-01 |
| LPC(18:0e) | -0.066 | 0.069 | 2.454 | -0.608 | 5.46E-01 | 6.18E-01 |
| LPC(18:1e) | -0.086 | 0.09 | 1.563 | -0.798 | 4.28E-01 | 5.00E-01 |
| LPC(18:2) | 0.024 | -0.026 | 2.387 | 0.226 | 8.22E-01 | 8.66E-01 |
| LPC(18:3) | 0.019 | -0.02 | 1.096 | 0.174 | 8.62E-01 | 8.97E-01 |
| LPC(18:4) | 0.225 | -0.237 | 0.442 | 2.17 | 3.30E-02 | 5.96E-02 |
| LPC(20:0) | 0.218 | -0.229 | 0.276 | 2.11 | 3.83E-02 | 6.82E-02 |
| LPC(20:1) | -0.215 | 0.226 | 3.736 | -2.051 | 4.38E-02 | 7.57E-02 |
| LPC(20:2) | 0.023 | -0.024 | 1.267 | 0.215 | 8.31E-01 | 8.70E-01 |
| LPC(20:3) | 0.051 | -0.054 | 0.926 | 0.48 | 6.32E-01 | 7.00E-01 |
| LPC(20:4) | -0.01 | 0.011 | 0.806 | -0.096 | 9.24E-01 | 9.44E-01 |
| LPC(20:5) | 0.048 | -0.05 | 0.706 | 0.45 | 6.54E-01 | 7.17E-01 |
| LPC(22:1) | 0.11 | -0.115 | 0.666 | 1.033 | 3.05E-01 | 3.83E-01 |
| LPC(22:5) | 0.036 | -0.038 | 1.393 | 0.336 | 7.38E-01 | 7.97E-01 |
| LPC(22:6) | 0.183 | -0.192 | 0.737 | 1.737 | 8.63E-02 | 1.33E-01 |
| LPC(24:0) | 0.099 | -0.104 | 0.337 | 0.941 | 3.50E-01 | 4.28E-01 |
| LPC(26:0) | -0.083 | 0.087 | 1.852 | -0.77 | 4.44E-01 | 5.16E-01 |
| PC(14:0_18:2) | -0.21 | 0.22 | 2.871 | -1.979 | 5.28E-02 | 8.85E-02 |
| PC(14:1e_18:1) | -0.257 | 0.27 | 3.121 | -2.482 | 1.53E-02 | 3.15E-02 |
| PC(14:1e_18:2) | 0.177 | -0.186 | 0.697 | 1.69 | 9.50E-02 | 1.43E-01 |
| PC(14:1e_20:4) | -0.242 | 0.254 | 1.737 | -2.347 | 2.14E-02 | 4.17E-02 |
| PC(15:0_18:1) | 0.233 | -0.244 | 0.443 | 2.252 | 2.71E-02 | 5.07E-02 |
| PC(15:0_18:2) | 0.411 | -0.432 | 0.415 | 4.257 | 5.59E-05 | 3.27E-04 |
| PC(15:0_20:2) | 0.386 | -0.405 | 0.502 | 3.953 | 1.67E-04 | 7.26E-04 |
| PC(16:0_16:1) | -0.195 | 0.205 | 3.443 | -1.84 | 7.04E-02 | 1.13E-01 |
| PC(16:0_18:1) | -0.216 | 0.227 | 3.212 | -2.037 | 4.66E-02 | 7.96E-02 |
| PC(16:0_18:2) | -0.077 | 0.081 | 1.942 | -0.714 | 4.78E-01 | 5.50E-01 |
| PC(16:0_20:3) | -0.248 | 0.261 | 3.209 | -2.387 | 1.96E-02 | 3.88E-02 |
| PC(16:0_20:4) | -0.252 | 0.264 | 2.912 | -2.402 | 1.96E-02 | 3.88E-02 |
| PC(16:0_20:5) | -0.257 | 0.269 | 3.048 | -2.453 | 1.71E-02 | 3.47E-02 |
| PC(16:0_22:6) | -0.391 | 0.411 | 4.892 | -3.962 | 1.88E-04 | 7.83E-04 |
| PC(16:1_18:1) | -0.09 | 0.094 | 2.842 | -0.83 | 4.10E-01 | 4.85E-01 |
| PC(16:1_20:3) | 0.197 | -0.207 | 0.305 | 1.908 | 6.09E-02 | 9.95E-02 |
| PC(16:1_22:5) | -0.218 | 0.229 | 4.045 | -2.067 | 4.32E-02 | 7.51E-02 |
| PC(16:1e_20:3) | -0.21 | 0.221 | 3.119 | -1.985 | 5.19E-02 | 8.71E-02 |
| PC(16:2e_16:0) | 0.342 | -0.359 | 0.432 | 3.446 | 9.41E-04 | 3.07E-03 |
| PC(16:2e_18:2) | 0.131 | -0.138 | 0.397 | 1.251 | 2.15E-01 | 2.88E-01 |
| PC(18:0_15:0) | -0.157 | 0.165 | 1.7 | -1.491 | 1.40E-01 | 2.01E-01 |
| PC(18:0_18:2) | -0.214 | 0.224 | 2.887 | -2.02 | 4.79E-02 | 8.14E-02 |
| PC(18:0_20:3) | -0.27 | 0.283 | 2.825 | -2.594 | 1.18E-02 | 2.49E-02 |
| PC(18:0_20:4) | -0.306 | 0.321 | 4.209 | -2.971 | 4.35E-03 | 1.11E-02 |
| PC(18:0_20:5) | -0.232 | 0.244 | 2.79 | -2.22 | 2.96E-02 | 5.45E-02 |
| PC(18:0_22:4) | -0.133 | 0.14 | 3.732 | -1.24 | 2.20E-01 | 2.92E-01 |
| PC(18:0_22:5) | -0.217 | 0.228 | 2.66 | -2.058 | 4.36E-02 | 7.55E-02 |
| PC(18:0_22:6) | -0.218 | 0.229 | 3.694 | -2.064 | 4.32E-02 | 7.51E-02 |
| PC(18:1_13:0) | 0.349 | -0.367 | 0.45 | 3.512 | 7.34E-04 | 2.51E-03 |
| PC(18:1_18:1) | 0.089 | -0.094 | 0.636 | 0.854 | 3.97E-01 | 4.70E-01 |
| PC(18:1_20:3) | -0.312 | 0.328 | 2.983 | -3.049 | 3.31E-03 | 8.92E-03 |
| PC(18:1_20:4) | -0.259 | 0.272 | 3.236 | -2.489 | 1.54E-02 | 3.16E-02 |
| PC(18:2_13:0) | 0.182 | -0.191 | 0.667 | 1.747 | 8.47E-02 | 1.32E-01 |
| PC(18:2_18:2) | -0.32 | 0.336 | 4.524 | -3.125 | 2.74E-03 | 7.65E-03 |
| PC(18:2_20:4) | 0.271 | -0.284 | 0.569 | 2.612 | 1.10E-02 | 2.36E-02 |
| PC(19:3e) | -0.004 | 0.004 | 1.298 | -0.034 | 9.73E-01 | 9.83E-01 |
| PC(20:0_18:1) | 0.379 | -0.398 | 0.416 | 3.887 | 2.18E-04 | 8.95E-04 |
| PC(20:2_18:2) | 0.533 | -0.56 | 0.225 | 6.03 | 5.40E-08 | 3.55E-06 |
| PC(20:5_13:0) | 0.145 | -0.152 | 0.921 | 1.36 | 1.78E-01 | 2.46E-01 |
| PC(20:5_18:2) | -0.208 | 0.219 | 3.739 | -1.968 | 5.36E-02 | 8.96E-02 |
| PC(21:3e) | 0.217 | -0.228 | 0.529 | 2.076 | 4.13E-02 | 7.27E-02 |
| PC(22:0_11:3) | 0.254 | -0.267 | 0.47 | 2.48 | 1.54E-02 | 3.16E-02 |
| PC(22:4_14:1) | -0.195 | 0.205 | 1.58 | -1.837 | 7.12E-02 | 1.13E-01 |
| PC(28:0) | 0.283 | -0.297 | 0.464 | 2.78 | 6.80E-03 | 1.58E-02 |
| PC(28:1_6:0) | -0.094 | 0.099 | 4.132 | -0.867 | 3.90E-01 | 4.63E-01 |
| PC(30:0) | 0.225 | -0.236 | 0.557 | 2.184 | 3.24E-02 | 5.87E-02 |
| PC(30:1) | 0.011 | -0.012 | 0.814 | 0.106 | 9.16E-01 | 9.38E-01 |
| PC(30:2) | -0.258 | 0.271 | 2.261 | -2.494 | 1.48E-02 | 3.06E-02 |
| PC(31:0) | 0.353 | -0.37 | 0.316 | 3.566 | 6.32E-04 | 2.20E-03 |
| PC(31:1) | 0.119 | -0.125 | 0.788 | 1.136 | 2.60E-01 | 3.34E-01 |
| PC(32:0) | 0.334 | -0.351 | 0.533 | 3.364 | 1.25E-03 | 3.89E-03 |
| PC(32:1) | 0.426 | -0.447 | 0.346 | 4.454 | 2.71E-05 | 1.93E-04 |
| PC(32:2) | 0.298 | -0.313 | 0.383 | 2.963 | 4.16E-03 | 1.07E-02 |
| PC(32:3) | 0.177 | -0.186 | 0.747 | 1.689 | 9.53E-02 | 1.43E-01 |
| PC(32:4) | -0.066 | 0.069 | 1.002 | -0.622 | 5.36E-01 | 6.08E-01 |
| PC(33:0) | 0.171 | -0.18 | 0.352 | 1.65 | 1.04E-01 | 1.55E-01 |
| PC(33:1) | 0.39 | -0.41 | 0.537 | 4.025 | 1.35E-04 | 6.17E-04 |
| PC(33:2) | 0.352 | -0.37 | 0.488 | 3.559 | 6.37E-04 | 2.21E-03 |
| PC(33:3) | 0.088 | -0.092 | 0.754 | 0.825 | 4.12E-01 | 4.86E-01 |
| PC(34:0) | 0.34 | -0.357 | 0.37 | 3.404 | 1.04E-03 | 3.35E-03 |
| PC(34:1) | 0.518 | -0.544 | 0.368 | 5.749 | 1.61E-07 | 5.58E-06 |
| PC(34:2) | 0.569 | -0.598 | 0.4 | 6.616 | 3.83E-09 | 7.10E-07 |
| PC(34:3) | 0.245 | -0.258 | 0.429 | 2.401 | 1.92E-02 | 3.83E-02 |
| PC(34:4) | -0.046 | 0.049 | 0.413 | -0.44 | 6.62E-01 | 7.25E-01 |
| PC(34:5) | 0.305 | -0.32 | 0.327 | 3.029 | 3.41E-03 | 9.16E-03 |
| PC(35:1) | 0.422 | -0.443 | 0.439 | 4.395 | 3.38E-05 | 2.28E-04 |
| PC(35:3) | 0.417 | -0.438 | 0.254 | 4.339 | 4.16E-05 | 2.63E-04 |
| PC(35:4) | 0.487 | -0.511 | 0.37 | 5.285 | 1.06E-06 | 2.17E-05 |
| PC(35:5) | -0.174 | 0.183 | 1.547 | -1.661 | 1.01E-01 | 1.51E-01 |
| PC(36:0) | 0.445 | -0.468 | 0.287 | 4.734 | 1.01E-05 | 9.50E-05 |
| PC(36:1) | 0.5 | -0.525 | 0.32 | 5.487 | 4.67E-07 | 1.17E-05 |
| PC(36:2) | -0.386 | 0.406 | 4.05 | -3.907 | 2.19E-04 | 8.95E-04 |
| PC(36:3) | -0.246 | 0.259 | 2.068 | -2.34 | 2.31E-02 | 4.42E-02 |
| PC(36:4) | 0.429 | -0.451 | 0.39 | 4.503 | 2.27E-05 | 1.73E-04 |
| PC(36:5) | -0.01 | 0.011 | 1.45 | -0.095 | 9.25E-01 | 9.44E-01 |
| PC(36:6) | 0.406 | -0.427 | 0.343 | 4.212 | 6.69E-05 | 3.71E-04 |
| PC(37:1) | 0.201 | -0.211 | 0.324 | 1.937 | 5.67E-02 | 9.37E-02 |
| PC(37:2) | 0.278 | -0.292 | 0.323 | 2.75 | 7.81E-03 | 1.77E-02 |
| PC(37:3) | -0.243 | 0.255 | 3.148 | -2.323 | 2.33E-02 | 4.43E-02 |
| PC(37:4) | 0.278 | -0.292 | 0.43 | 2.742 | 7.67E-03 | 1.75E-02 |
| PC(37:5) | -0.059 | 0.062 | 1.211 | -0.545 | 5.87E-01 | 6.58E-01 |
| PC(37:6) | 0.115 | -0.121 | 0.685 | 1.08 | 2.83E-01 | 3.59E-01 |
| PC(38:1) | -0.197 | 0.207 | 4.809 | -1.854 | 6.90E-02 | 1.11E-01 |
| PC(38:2) | 0.285 | -0.299 | 0.363 | 2.83 | 6.32E-03 | 1.48E-02 |
| PC(38:3) | 0.189 | -0.199 | 0.372 | 1.813 | 7.37E-02 | 1.17E-01 |
| PC(38:4) | 0.348 | -0.366 | 0.314 | 3.529 | 7.46E-04 | 2.53E-03 |
| PC(38:5) | 0.123 | -0.13 | 0.43 | 1.184 | 2.42E-01 | 3.16E-01 |
| PC(38:6) | 0.181 | -0.191 | 0.579 | 1.719 | 8.96E-02 | 1.38E-01 |
| PC(38:7) | 0.301 | -0.316 | 0.229 | 2.977 | 3.91E-03 | 1.02E-02 |
| PC(38:8) | 0.369 | -0.388 | 0.437 | 3.733 | 3.55E-04 | 1.34E-03 |
| PC(38:9) | 0.118 | -0.124 | 0.471 | 1.12 | 2.67E-01 | 3.41E-01 |
| PC(39:3) | -0.165 | 0.173 | 3.56 | -1.54 | 1.29E-01 | 1.88E-01 |
| PC(39:4) | -0.078 | 0.081 | 1.21 | -0.722 | 4.73E-01 | 5.45E-01 |
| PC(39:5) | -0.033 | 0.034 | 0.568 | -0.308 | 7.59E-01 | 8.17E-01 |
| PC(39:6) | -0.128 | 0.134 | 3.543 | -1.189 | 2.39E-01 | 3.14E-01 |
| PC(39:7) | 0.133 | -0.14 | 0.673 | 1.259 | 2.12E-01 | 2.84E-01 |
| PC(40:1) | -0.165 | 0.174 | 1.071 | -1.581 | 1.18E-01 | 1.73E-01 |
| PC(40:10) | -0.078 | 0.082 | 1.778 | -0.734 | 4.65E-01 | 5.37E-01 |
| PC(40:2) | -0.161 | 0.169 | 1.431 | -1.529 | 1.30E-01 | 1.89E-01 |
| PC(40:3) | -0.122 | 0.128 | 0.308 | -1.166 | 2.48E-01 | 3.23E-01 |
| PC(40:4) | 0.308 | -0.324 | 0.352 | 3.056 | 3.06E-03 | 8.39E-03 |
| PC(40:5) | 0.323 | -0.339 | 0.526 | 3.185 | 2.09E-03 | 6.10E-03 |
| PC(40:6) | 0.477 | -0.501 | 0.363 | 5.13 | 2.04E-06 | 3.01E-05 |
| PC(40:7) | 0.2 | -0.21 | 0.435 | 1.933 | 5.75E-02 | 9.48E-02 |
| PC(40:8) | 0.13 | -0.137 | 0.553 | 1.248 | 2.17E-01 | 2.89E-01 |
| PC(40:9) | 0.231 | -0.242 | 0.612 | 2.224 | 2.90E-02 | 5.38E-02 |
| PC(41:5) | -0.077 | 0.08 | 3.019 | -0.709 | 4.81E-01 | 5.53E-01 |
| PC(41:6) | -0.196 | 0.206 | 1.406 | -1.886 | 6.32E-02 | 1.02E-01 |
| PC(42:10) | -0.125 | 0.132 | 4.259 | -1.163 | 2.50E-01 | 3.25E-01 |
| PC(42:11) | -0.127 | 0.134 | 2.558 | -1.188 | 2.39E-01 | 3.14E-01 |
| PC(42:3) | 0.041 | -0.043 | 0.46 | 0.389 | 6.99E-01 | 7.61E-01 |
| PC(42:5) | -0.031 | 0.032 | 1.028 | -0.286 | 7.76E-01 | 8.30E-01 |
| PC(42:6) | -0.026 | 0.027 | 2.162 | -0.238 | 8.12E-01 | 8.58E-01 |
| PC(42:7) | 0.351 | -0.368 | 0.315 | 3.536 | 6.87E-04 | 2.37E-03 |
| PC(42:8) | 0.138 | -0.145 | 0.47 | 1.31 | 1.94E-01 | 2.64E-01 |
| PC(42:9) | 0.316 | -0.332 | 0.384 | 3.145 | 2.35E-03 | 6.68E-03 |
| PC(44:10) | 0.296 | -0.311 | 0.512 | 2.918 | 4.58E-03 | 1.15E-02 |
| PC(44:11) | -0.119 | 0.125 | 1.452 | -1.112 | 2.69E-01 | 3.44E-01 |
| PC(44:12) | -0.158 | 0.165 | 3.593 | -1.471 | 1.46E-01 | 2.08E-01 |
| PC(44:5) | 0.396 | -0.416 | 0.338 | 4.085 | 1.06E-04 | 5.15E-04 |
| PE(16:0_18:1) | -0.247 | 0.259 | 2.746 | -2.355 | 2.16E-02 | 4.19E-02 |
| PE(16:0_18:2) | -0.125 | 0.132 | 3.891 | -1.166 | 2.48E-01 | 3.23E-01 |
| PE(16:0_20:3) | -0.255 | 0.268 | 3.286 | -2.445 | 1.74E-02 | 3.49E-02 |
| PE(16:0_20:4) | 0.272 | -0.285 | 0.368 | 2.656 | 9.55E-03 | 2.09E-02 |
| PE(16:0_22:6) | 0.124 | -0.13 | 0.54 | 1.175 | 2.44E-01 | 3.18E-01 |
| PE(16:0e) | -0.232 | 0.243 | 3.097 | -2.203 | 3.14E-02 | 5.73E-02 |
| PE(16:0p_18:1) | 0.256 | -0.269 | 0.245 | 2.497 | 1.46E-02 | 3.03E-02 |
| PE(16:0p_18:2) | 0.18 | -0.189 | 0.358 | 1.725 | 8.86E-02 | 1.36E-01 |
| PE(16:0p_20:4) | 0.367 | -0.385 | 0.277 | 3.735 | 3.64E-04 | 1.37E-03 |
| PE(16:0p_20:5) | 0.229 | -0.24 | 0.387 | 2.211 | 2.99E-02 | 5.49E-02 |
| PE(16:0p_22:6) | 0.363 | -0.381 | 0.225 | 3.687 | 4.19E-04 | 1.53E-03 |
| PE(17:1e) | -0.144 | 0.151 | 2.27 | -1.347 | 1.83E-01 | 2.50E-01 |
| PE(18:0_18:1) | -0.285 | 0.3 | 4.802 | -2.753 | 7.86E-03 | 1.78E-02 |
| PE(18:0_20:4) | 0.18 | -0.189 | 0.324 | 1.715 | 9.02E-02 | 1.38E-01 |
| PE(18:0_22:6) | 0.23 | -0.241 | 0.344 | 2.215 | 2.96E-02 | 5.45E-02 |
| PE(18:0p_18:1) | 0.297 | -0.311 | 0.377 | 2.919 | 4.56E-03 | 1.15E-02 |
| PE(18:0p_18:2) | 0.277 | -0.291 | 0.323 | 2.714 | 8.15E-03 | 1.83E-02 |
| PE(18:0p_20:3) | 0.137 | -0.144 | 0.378 | 1.305 | 1.96E-01 | 2.66E-01 |
| PE(18:0p_20:4) | 0.237 | -0.249 | 0.233 | 2.298 | 2.43E-02 | 4.60E-02 |
| PE(18:0p_20:5) | 0.212 | -0.222 | 0.357 | 2.037 | 4.50E-02 | 7.72E-02 |
| PE(18:0p_22:4) | 0.349 | -0.366 | 0.25 | 3.515 | 7.38E-04 | 2.51E-03 |
| PE(18:0p_22:5) | 0.212 | -0.222 | 0.247 | 2.04 | 4.48E-02 | 7.69E-02 |
| PE(18:0p_22:6) | 0.202 | -0.212 | 0.256 | 1.943 | 5.57E-02 | 9.25E-02 |
| PE(18:1e) | -0.045 | 0.047 | 3.322 | -0.412 | 6.82E-01 | 7.45E-01 |
| PE(18:1e_22:6) | -0.304 | 0.319 | 2.569 | -2.965 | 4.21E-03 | 1.08E-02 |
| PE(18:1p_18:1) | -0.298 | 0.313 | 3.768 | -2.891 | 5.26E-03 | 1.27E-02 |
| PE(18:1p_18:2) | 0.015 | -0.016 | 0.656 | 0.144 | 8.86E-01 | 9.12E-01 |
| PE(18:1p_20:4) | 0.221 | -0.232 | 0.357 | 2.141 | 3.55E-02 | 6.35E-02 |
| PE(18:1p_20:5) | -0.023 | 0.024 | 0.677 | -0.217 | 8.29E-01 | 8.70E-01 |
| PE(18:1p_22:6) | 0.183 | -0.192 | 0.284 | 1.754 | 8.34E-02 | 1.31E-01 |
| PE(18:2e) | -0.172 | 0.181 | 2.394 | -1.619 | 1.10E-01 | 1.64E-01 |
| PE(19:4e) | 0.125 | -0.131 | 0.301 | 1.188 | 2.39E-01 | 3.14E-01 |
| PE(20:0p_20:4) | 0.214 | -0.225 | 0.287 | 2.067 | 4.21E-02 | 7.40E-02 |
| PE(20:0p_20:5) | -0.184 | 0.193 | 3.173 | -1.722 | 9.05E-02 | 1.38E-01 |
| PE(20:0p_22:5) | 0.173 | -0.182 | 0.277 | 1.655 | 1.02E-01 | 1.53E-01 |
| PE(20:0p_22:6) | 0.294 | -0.309 | 0.277 | 2.896 | 4.88E-03 | 1.20E-02 |
| PE(20:1p_22:6) | -0.138 | 0.145 | 1.137 | -1.304 | 1.96E-01 | 2.66E-01 |
| PE(20:3e) | -0.144 | 0.151 | 1.869 | -1.358 | 1.78E-01 | 2.46E-01 |
| PI(16:0_16:1) | -0.017 | 0.018 | 1.274 | -0.164 | 8.70E-01 | 9.03E-01 |
| PI(16:0_18:1) | 0.297 | -0.312 | 0.447 | 2.926 | 4.48E-03 | 1.14E-02 |
| PI(16:0_18:2) | -0.104 | 0.11 | 1.545 | -0.982 | 3.29E-01 | 4.09E-01 |
| PI(16:0_20:3) | -0.363 | 0.381 | 3.299 | -3.636 | 5.20E-04 | 1.85E-03 |
| PI(16:0_20:4) | 0.004 | -0.005 | 1.179 | 0.042 | 9.67E-01 | 9.81E-01 |
| PI(16:0_22:6) | -0.06 | 0.063 | 1.328 | -0.562 | 5.76E-01 | 6.46E-01 |
| PI(18:0_18:1) | 0.408 | -0.429 | 0.458 | 4.217 | 6.47E-05 | 3.62E-04 |
| PI(18:0_18:2) | 0.57 | -0.598 | 0.268 | 6.621 | 3.77E-09 | 7.10E-07 |
| PI(18:0_18:3) | -0.299 | 0.314 | 4.55 | -2.92 | 4.66E-03 | 1.17E-02 |
| PI(18:0_20:3) | -0.136 | 0.143 | 1.434 | -1.27 | 2.09E-01 | 2.81E-01 |
| PI(18:0_20:4) | -0.438 | 0.459 | 1.966 | -4.575 | 1.80E-05 | 1.46E-04 |
| PI(18:0_22:5) | 0.208 | -0.219 | 0.946 | 1.986 | 5.06E-02 | 8.51E-02 |
| PI(18:0_22:6) | -0.025 | 0.026 | 1.404 | -0.228 | 8.21E-01 | 8.66E-01 |
| PI(18:1_18:2) | -0.273 | 0.287 | 3.004 | -2.631 | 1.07E-02 | 2.29E-02 |
| PI(18:1_20:4) | -0.426 | 0.447 | 4.138 | -4.391 | 4.33E-05 | 2.67E-04 |
| PI(34:1) | -0.274 | 0.288 | 3.907 | -2.632 | 1.09E-02 | 2.33E-02 |
| PI(36:3) | -0.236 | 0.248 | 2.033 | -2.281 | 2.52E-02 | 4.75E-02 |
| SM(d16:1_20:0) | 0.53 | -0.556 | 0.264 | 5.927 | 7.71E-08 | 3.80E-06 |
| SM(d16:1_24:3) | 0.504 | -0.529 | 0.256 | 5.531 | 3.99E-07 | 1.09E-05 |
| SM(d17:1_13:0) | 0.302 | -0.317 | 0.666 | 2.979 | 3.83E-03 | 1.01E-02 |
| SM(d17:1_18:3) | 0.481 | -0.505 | 0.342 | 5.199 | 1.50E-06 | 2.52E-05 |
| SM(d18:0_16:1) | 0.534 | -0.561 | 0.296 | 5.994 | 6.15E-08 | 3.73E-06 |
| SM(d18:1_18:3) | 0.447 | -0.469 | 0.46 | 4.671 | 1.54E-05 | 1.32E-04 |
| SM(d18:1_21:0) | -0.441 | 0.463 | 4.49 | -4.601 | 1.83E-05 | 1.47E-04 |
| SM(d18:1_24:0) | -0.352 | 0.369 | 3.048 | -3.492 | 8.83E-04 | 2.91E-03 |
| SM(d18:1_24:3) | 0.524 | -0.55 | 0.25 | 5.84 | 1.11E-07 | 4.86E-06 |
| SM(d18:2_16:0) | -0.486 | 0.511 | 3.938 | -5.235 | 1.61E-06 | 2.53E-05 |
| SM(d18:2_18:3) | 0.438 | -0.46 | 0.334 | 4.614 | 1.48E-05 | 1.30E-04 |
| SM(d18:2_20:4) | 0.049 | -0.051 | 0.782 | 0.455 | 6.51E-01 | 7.16E-01 |
| SM(d18:2_24:3) | 0.355 | -0.373 | 0.404 | 3.602 | 5.73E-04 | 2.00E-03 |
| SM(d28:1) | 0.386 | -0.406 | 0.275 | 3.962 | 1.62E-04 | 7.08E-04 |
| SM(d30:1) | -0.414 | 0.435 | 3.386 | -4.257 | 6.36E-05 | 3.58E-04 |
| SM(d30:2) | 0.162 | -0.17 | 0.453 | 1.552 | 1.25E-01 | 1.83E-01 |
| SM(d31:1) | -0.423 | 0.444 | 5.01 | -4.376 | 3.87E-05 | 2.48E-04 |
| SM(d32:0) | 0.431 | -0.453 | 0.389 | 4.511 | 2.18E-05 | 1.67E-04 |
| SM(d32:1) | 0.113 | -0.118 | 0.938 | 1.069 | 2.89E-01 | 3.64E-01 |
| SM(d32:2) | 0.099 | -0.104 | 0.997 | 0.926 | 3.58E-01 | 4.35E-01 |
| SM(d32:4) | 0.401 | -0.421 | 0.44 | 4.133 | 8.75E-05 | 4.46E-04 |
| SM(d33:0) | -0.287 | 0.301 | 2.699 | -2.781 | 6.97E-03 | 1.61E-02 |
| SM(d33:1) | 0.404 | -0.424 | 0.47 | 4.153 | 8.28E-05 | 4.30E-04 |
| SM(d33:2) | 0.086 | -0.09 | 0.663 | 0.812 | 4.19E-01 | 4.93E-01 |
| SM(d34:0) | 0.458 | -0.481 | 0.32 | 4.859 | 6.06E-06 | 6.73E-05 |
| SM(d34:1) | -0.413 | 0.433 | 5.255 | -4.23 | 7.30E-05 | 3.85E-04 |
| SM(d34:2) | 0.407 | -0.427 | 0.459 | 4.203 | 6.81E-05 | 3.73E-04 |
| SM(d34:3) | 0.201 | -0.211 | 0.405 | 1.947 | 5.60E-02 | 9.27E-02 |
| SM(d34:4) | 0.393 | -0.413 | 0.44 | 4.031 | 1.26E-04 | 5.91E-04 |
| SM(d34:5) | 0.271 | -0.285 | 0.344 | 2.65 | 9.72E-03 | 2.12E-02 |
| SM(d35:0) | 0.165 | -0.173 | 0.554 | 1.566 | 1.21E-01 | 1.78E-01 |
| SM(d35:1) | 0.313 | -0.329 | 0.361 | 3.122 | 2.57E-03 | 7.23E-03 |
| SM(d35:2) | 0.421 | -0.443 | 0.322 | 4.389 | 3.45E-05 | 2.29E-04 |
| SM(d35:4) | -0.315 | 0.331 | 5.393 | -3.073 | 3.24E-03 | 8.81E-03 |
| SM(d36:0) | 0.239 | -0.251 | 0.454 | 2.322 | 2.29E-02 | 4.40E-02 |
| SM(d36:1) | 0.284 | -0.298 | 0.463 | 2.809 | 6.49E-03 | 1.51E-02 |
| SM(d36:2) | 0.452 | -0.474 | 0.388 | 4.791 | 7.50E-06 | 7.49E-05 |
| SM(d36:3) | 0.286 | -0.3 | 0.38 | 2.832 | 6.14E-03 | 1.44E-02 |
| SM(d36:5) | -0.503 | 0.528 | 3.778 | -5.489 | 5.57E-07 | 1.32E-05 |
| SM(d37:1) | 0.374 | -0.392 | 0.375 | 3.77 | 3.25E-04 | 1.25E-03 |
| SM(d37:2) | 0.424 | -0.445 | 0.358 | 4.403 | 3.31E-05 | 2.27E-04 |
| SM(d37:5) | 0.198 | -0.207 | 0.484 | 1.894 | 6.19E-02 | 1.01E-01 |
| SM(d38:0) | -0.035 | 0.037 | 1.17 | -0.323 | 7.47E-01 | 8.05E-01 |
| SM(d38:1) | 0.525 | -0.551 | 0.252 | 5.852 | 1.03E-07 | 4.80E-06 |
| SM(d38:2) | 0.416 | -0.437 | 0.414 | 4.313 | 4.59E-05 | 2.76E-04 |
| SM(d38:3) | 0.313 | -0.329 | 0.361 | 3.123 | 2.57E-03 | 7.23E-03 |
| SM(d38:5) | 0.229 | -0.241 | 0.419 | 2.229 | 2.89E-02 | 5.38E-02 |
| SM(d39:1) | 0.481 | -0.505 | 0.284 | 5.195 | 1.53E-06 | 2.52E-05 |
| SM(d39:2) | 0.393 | -0.412 | 0.421 | 4.012 | 1.37E-04 | 6.22E-04 |
| SM(d40:1) | 0.456 | -0.479 | 0.366 | 4.825 | 6.90E-06 | 7.16E-05 |
| SM(d40:2) | 0.322 | -0.338 | 0.566 | 3.209 | 1.93E-03 | 5.68E-03 |
| SM(d40:3) | 0.25 | -0.262 | 0.318 | 2.432 | 1.74E-02 | 3.49E-02 |
| SM(d40:4) | -0.521 | 0.547 | 4.119 | -5.771 | 1.63E-07 | 5.58E-06 |
| SM(d41:0) | 0.422 | -0.444 | 0.428 | 4.392 | 3.42E-05 | 2.29E-04 |
| SM(d41:1) | 0.504 | -0.529 | 0.242 | 5.537 | 3.80E-07 | 1.07E-05 |
| SM(d41:2) | 0.078 | -0.082 | 0.834 | 0.736 | 4.64E-01 | 5.37E-01 |
| SM(d41:3) | 0.412 | -0.433 | 0.437 | 4.263 | 5.52E-05 | 3.25E-04 |
| SM(d41:4) | 0.418 | -0.439 | 0.245 | 4.332 | 4.34E-05 | 2.67E-04 |
| SM(d42:1) | 0.444 | -0.466 | 0.28 | 4.699 | 1.11E-05 | 1.03E-04 |
| SM(d42:2) | 0.477 | -0.501 | 0.327 | 5.129 | 2.02E-06 | 3.01E-05 |
| SM(d42:3) | 0.372 | -0.39 | 0.431 | 3.765 | 3.18E-04 | 1.23E-03 |
| SM(d42:4) | -0.459 | 0.482 | 3.458 | -4.852 | 6.58E-06 | 7.06E-05 |
| SM(d42:5) | -0.121 | 0.128 | 4.134 | -1.13 | 2.63E-01 | 3.37E-01 |
| SM(d43:1) | 0.302 | -0.317 | 0.409 | 2.974 | 3.89E-03 | 1.02E-02 |
| SM(d43:2) | 0.372 | -0.39 | 0.251 | 3.768 | 3.14E-04 | 1.22E-03 |
| SM(d43:3) | 0.417 | -0.438 | 0.419 | 4.325 | 4.38E-05 | 2.68E-04 |
| SM(d43:4) | 0.447 | -0.469 | 0.28 | 4.723 | 9.77E-06 | 9.29E-05 |
| SM(d43:5) | 0.189 | -0.199 | 0.7 | 1.804 | 7.50E-02 | 1.19E-01 |
| SM(d44:1) | 0.135 | -0.141 | 0.951 | 1.264 | 2.10E-01 | 2.83E-01 |
| SM(d44:2) | -0.243 | 0.255 | 2.321 | -2.324 | 2.32E-02 | 4.43E-02 |
| SM(d44:3) | -0.397 | 0.417 | 2.948 | -4.044 | 1.30E-04 | 6.00E-04 |
| SM(d44:4) | 0.427 | -0.448 | 0.31 | 4.473 | 2.55E-05 | 1.88E-04 |
| SM(d44:5) | 0.459 | -0.481 | 0.323 | 4.877 | 5.41E-06 | 6.19E-05 |
| SM(d44:6) | 0.427 | -0.448 | 0.357 | 4.462 | 2.63E-05 | 1.90E-04 |
| SM(t18:0_16:1) | 0.351 | -0.369 | 0.545 | 3.516 | 7.34E-04 | 2.51E-03 |
| SM(t18:0_23:1) | -0.207 | 0.218 | 3.544 | -1.96 | 5.43E-02 | 9.05E-02 |
| SM(t18:0_24:2) | 0.365 | -0.384 | 0.427 | 3.691 | 4.08E-04 | 1.51E-03 |
| SM(t32:1) | 0.279 | -0.293 | 0.686 | 2.711 | 8.29E-03 | 1.86E-02 |
| SM(t34:0) | 0.38 | -0.399 | 0.51 | 3.879 | 2.14E-04 | 8.85E-04 |
| SM(t34:1) | 0.423 | -0.444 | 0.502 | 4.388 | 3.52E-05 | 2.32E-04 |
| SM(t34:2) | 0.374 | -0.392 | 0.409 | 3.798 | 2.83E-04 | 1.12E-03 |
| SM(t36:1) | 0.298 | -0.312 | 0.618 | 2.912 | 4.70E-03 | 1.17E-02 |
| SM(t36:2) | -0.344 | 0.361 | 4.338 | -3.406 | 1.12E-03 | 3.56E-03 |
| SM(t38:3) | -0.405 | 0.425 | 3.52 | -4.152 | 8.75E-05 | 4.46E-04 |
| SM(t38:6) | -0.012 | 0.013 | 1.031 | -0.113 | 9.10E-01 | 9.34E-01 |
| SM(t39:5) | 0.202 | -0.212 | 0.413 | 1.94 | 5.60E-02 | 9.27E-02 |
| SM(t39:6) | -0.25 | 0.262 | 3.886 | -2.389 | 2.00E-02 | 3.93E-02 |
| SM(t40:6) | -0.128 | 0.135 | 3.404 | -1.19 | 2.39E-01 | 3.14E-01 |
| SM(t40:7) | -0.014 | 0.015 | 0.393 | -0.137 | 8.92E-01 | 9.17E-01 |
| SM(t42:1) | 0.393 | -0.413 | 0.319 | 4.038 | 1.23E-04 | 5.84E-04 |
| TG(11:0_18:0_18:0) | -0.053 | 0.055 | 1.916 | -0.487 | 6.28E-01 | 6.96E-01 |
| TG(11:0_9:0_9:0) | -0.264 | 0.277 | 2.903 | -2.549 | 1.29E-02 | 2.70E-02 |
| TG(12:0_12:0_14:0) | 0.268 | -0.282 | 0.201 | 2.623 | 1.05E-02 | 2.26E-02 |
| TG(12:0_12:0_18:2) | 0.004 | -0.005 | 0.719 | 0.042 | 9.66E-01 | 9.81E-01 |
| TG(12:0_14:0_18:3) | -0.017 | 0.017 | 3.052 | -0.153 | 8.79E-01 | 9.09E-01 |
| TG(12:0_14:0_20:5) | 0.006 | -0.006 | 0.902 | 0.057 | 9.55E-01 | 9.71E-01 |
| TG(12:0_14:0_22:6) | 0.156 | -0.164 | 0.722 | 1.478 | 1.43E-01 | 2.05E-01 |
| TG(12:0_17:1_18:2) | -0.285 | 0.299 | 4.115 | -2.757 | 7.57E-03 | 1.74E-02 |
| TG(12:0_18:2_18:2) | -0.284 | 0.299 | 2.79 | -2.752 | 7.68E-03 | 1.75E-02 |
| TG(12:0_18:2_20:5) | 0.095 | -0.1 | 0.657 | 0.901 | 3.70E-01 | 4.44E-01 |
| TG(12:0_18:3_18:3) | -0.202 | 0.212 | 2.853 | -1.908 | 6.06E-02 | 9.92E-02 |
| TG(12:0_18:3_20:5) | 0.159 | -0.167 | 0.458 | 1.513 | 1.34E-01 | 1.95E-01 |
| TG(12:0_18:3_22:6) | -0.008 | 0.008 | 1.26 | -0.074 | 9.41E-01 | 9.58E-01 |
| TG(14:0_14:1_22:6) | -0.144 | 0.151 | 2.834 | -1.351 | 1.81E-01 | 2.48E-01 |
| TG(14:0_14:3_18:2) | -0.299 | 0.314 | 3.714 | -2.905 | 5.03E-03 | 1.23E-02 |
| TG(14:0_17:1_20:5) | -0.039 | 0.041 | 1.098 | -0.367 | 7.15E-01 | 7.77E-01 |
| TG(14:0_18:2_18:3) | -0.017 | 0.017 | 1.198 | -0.154 | 8.78E-01 | 9.09E-01 |
| TG(14:0_18:2_20:5) | -0.467 | 0.49 | 4.98 | -4.99 | 3.51E-06 | 4.37E-05 |
| TG(14:0_18:3_18:3) | -0.403 | 0.423 | 4.507 | -4.102 | 1.19E-04 | 5.69E-04 |
| TG(14:0_18:3_20:5) | -0.138 | 0.145 | 1.325 | -1.301 | 1.97E-01 | 2.67E-01 |
| TG(14:0_18:3_22:6) | -0.218 | 0.229 | 2.082 | -2.079 | 4.13E-02 | 7.27E-02 |
| TG(14:0_20:5_20:5) | 0.056 | -0.059 | 0.692 | 0.528 | 5.99E-01 | 6.68E-01 |
| TG(14:0e_18:0_20:1) | -0.279 | 0.293 | 3.686 | -2.695 | 8.87E-03 | 1.96E-02 |
| TG(15:0_12:0_14:0) | -0.147 | 0.154 | 1.993 | -1.384 | 1.70E-01 | 2.38E-01 |
| TG(15:0_12:0_16:0) | -0.158 | 0.166 | 2.401 | -1.488 | 1.41E-01 | 2.02E-01 |
| TG(15:0_12:0_16:1) | -0.12 | 0.126 | 1.637 | -1.123 | 2.65E-01 | 3.40E-01 |
| TG(15:0_14:0_18:2) | 0.19 | -0.2 | 0.719 | 1.819 | 7.27E-02 | 1.16E-01 |
| TG(15:0_14:0_18:3) | -0.179 | 0.188 | 2.957 | -1.686 | 9.65E-02 | 1.45E-01 |
| TG(15:0_14:1_16:1) | -0.311 | 0.327 | 4.571 | -3.039 | 3.43E-03 | 9.17E-03 |
| TG(15:0_15:0_15:0) | 0.268 | -0.281 | 0.676 | 2.594 | 1.13E-02 | 2.41E-02 |
| TG(15:0_16:0_16:0) | 0.256 | -0.268 | 0.82 | 2.47 | 1.57E-02 | 3.20E-02 |
| TG(15:0_16:0_16:1) | 0.276 | -0.29 | 0.815 | 2.677 | 9.11E-03 | 2.01E-02 |
| TG(15:0_16:0_18:1) | 0.324 | -0.34 | 0.645 | 3.226 | 1.83E-03 | 5.45E-03 |
| TG(15:0_16:0_18:3) | -0.261 | 0.274 | 2.729 | -2.516 | 1.40E-02 | 2.93E-02 |
| TG(15:0_16:0_20:5) | -0.399 | 0.419 | 4.159 | -4.084 | 1.06E-04 | 5.15E-04 |
| TG(15:0_16:0_22:6) | 0.14 | -0.147 | 0.553 | 1.325 | 1.89E-01 | 2.58E-01 |
| TG(15:0_16:1_18:1) | -0.084 | 0.088 | 0.906 | -0.787 | 4.34E-01 | 5.06E-01 |
| TG(15:0_16:1_18:2) | 0.308 | -0.324 | 0.374 | 3.072 | 3.02E-03 | 8.31E-03 |
| TG(15:0_16:1_18:3) | -0.056 | 0.059 | 0.593 | -0.53 | 5.98E-01 | 6.68E-01 |
| TG(15:0_16:1_20:5) | -0.276 | 0.289 | 2.638 | -2.663 | 9.71E-03 | 2.12E-02 |
| TG(15:0_18:1_20:4) | 0.574 | -0.603 | 0.24 | 6.705 | 2.61E-09 | 7.10E-07 |
| TG(15:0_18:1_22:5) | -0.102 | 0.107 | 1.518 | -0.949 | 3.46E-01 | 4.26E-01 |
| TG(15:0_18:1_22:6) | 0.423 | -0.444 | 0.277 | 4.406 | 3.24E-05 | 2.24E-04 |
| TG(15:0_18:2_18:2) | -0.331 | 0.347 | 2.77 | -3.25 | 1.86E-03 | 5.53E-03 |
| TG(15:0_18:2_18:3) | -0.214 | 0.225 | 3.552 | -2.024 | 4.73E-02 | 8.06E-02 |
| TG(15:0_18:2_20:5) | 0.372 | -0.391 | 0.391 | 3.725 | 4.25E-04 | 1.55E-03 |
| TG(15:0_18:2_22:6) | 0.13 | -0.137 | 0.568 | 1.226 | 2.24E-01 | 2.96E-01 |
| TG(15:0_6:0_10:0) | -0.069 | 0.072 | 0.733 | -0.647 | 5.20E-01 | 5.93E-01 |
| TG(15:0_8:0_10:0) | 0.03 | -0.032 | 0.347 | 0.284 | 7.78E-01 | 8.31E-01 |
| TG(16:0_11:1_18:1) | 0.37 | -0.388 | 0.334 | 3.754 | 3.29E-04 | 1.26E-03 |
| TG(16:0_12:0_17:1) | 0.427 | -0.449 | 0.345 | 4.468 | 2.57E-05 | 1.88E-04 |
| TG(16:0_12:0_18:3) | -0.023 | 0.024 | 1.578 | -0.209 | 8.35E-01 | 8.72E-01 |
| TG(16:0_12:0_20:4) | -0.186 | 0.196 | 2.424 | -1.755 | 8.39E-02 | 1.31E-01 |
| TG(16:0_12:0_22:6) | 0.096 | -0.101 | 0.853 | 0.903 | 3.69E-01 | 4.44E-01 |
| TG(16:0_12:1_18:1) | -0.283 | 0.298 | 3.876 | -2.74 | 7.94E-03 | 1.79E-02 |
| TG(16:0_13:0_16:1) | 0.388 | -0.407 | 0.353 | 3.966 | 1.58E-04 | 6.93E-04 |
| TG(16:0_14:0_14:0) | 0.317 | -0.333 | 0.368 | 3.153 | 2.28E-03 | 6.51E-03 |
| TG(16:0_14:0_16:0) | 0.39 | -0.41 | 0.252 | 4.005 | 1.39E-04 | 6.27E-04 |
| TG(16:0_14:0_18:1) | 0.459 | -0.482 | 0.223 | 4.898 | 4.97E-06 | 5.77E-05 |
| TG(16:0_14:0_18:3) | -0.413 | 0.433 | 3.956 | -4.23 | 7.21E-05 | 3.85E-04 |
| TG(16:0_14:0_20:5) | -0.157 | 0.165 | 2.838 | -1.471 | 1.47E-01 | 2.08E-01 |
| TG(16:0_14:1_16:1) | -0.253 | 0.266 | 4.267 | -2.427 | 1.80E-02 | 3.60E-02 |
| TG(16:0_14:1_18:2) | -0.314 | 0.33 | 3.375 | -3.066 | 3.23E-03 | 8.81E-03 |
| TG(16:0_14:3_18:3) | -0.137 | 0.144 | 1.662 | -1.275 | 2.07E-01 | 2.80E-01 |
| TG(16:0_16:0_16:0) | 0.378 | -0.397 | 0.227 | 3.854 | 2.34E-04 | 9.41E-04 |
| TG(16:0_16:0_17:0) | 0.302 | -0.317 | 0.57 | 2.973 | 3.90E-03 | 1.02E-02 |
| TG(16:0_16:0_18:1) | 0.391 | -0.411 | 0.386 | 4.011 | 1.35E-04 | 6.17E-04 |
| TG(16:0_16:0_18:2) | -0.271 | 0.284 | 2.839 | -2.599 | 1.18E-02 | 2.49E-02 |
| TG(16:0_16:0_18:3) | 0.399 | -0.419 | 0.295 | 4.098 | 9.96E-05 | 4.97E-04 |
| TG(16:0_16:0_20:4) | -0.456 | 0.479 | 4.508 | -4.821 | 7.22E-06 | 7.40E-05 |
| TG(16:0_16:0_20:5) | -0.354 | 0.372 | 3.273 | -3.521 | 7.85E-04 | 2.65E-03 |
| TG(16:0_16:0_23:0) | 0.323 | -0.339 | 0.308 | 3.205 | 1.94E-03 | 5.69E-03 |
| TG(16:0_16:0_24:0) | -0.365 | 0.383 | 1.968 | -3.683 | 4.20E-04 | 1.53E-03 |
| TG(16:0_16:0_24:1) | -0.301 | 0.316 | 1.828 | -2.953 | 4.17E-03 | 1.07E-02 |
| TG(16:0_17:0_18:1) | 0.325 | -0.341 | 0.665 | 3.224 | 1.83E-03 | 5.45E-03 |
| TG(16:0_17:0_20:4) | 0.503 | -0.529 | 0.298 | 5.541 | 3.75E-07 | 1.07E-05 |
| TG(16:0_17:1_18:1) | 0.47 | -0.494 | 0.341 | 5.032 | 3.03E-06 | 3.85E-05 |
| TG(16:0_17:1_18:3) | -0.468 | 0.491 | 3.224 | -4.987 | 3.68E-06 | 4.47E-05 |
| TG(16:0_17:1_20:5) | -0.266 | 0.279 | 2.411 | -2.565 | 1.24E-02 | 2.62E-02 |
| TG(16:0_18:1_18:1) | -0.051 | 0.053 | 0.94 | -0.475 | 6.36E-01 | 7.03E-01 |
| TG(16:0_18:1_18:2) | 0.388 | -0.408 | 0.367 | 3.971 | 1.56E-04 | 6.90E-04 |
| TG(16:0_18:1_18:3) | 0.448 | -0.47 | 0.234 | 4.753 | 8.89E-06 | 8.66E-05 |
| TG(16:0_18:1_19:0) | -0.548 | 0.576 | 3.487 | -6.239 | 2.03E-08 | 2.01E-06 |
| TG(16:0_18:1_20:3) | -0.469 | 0.493 | 2.916 | -5.003 | 3.55E-06 | 4.37E-05 |
| TG(16:0_18:1_20:4) | 0.208 | -0.219 | 0.52 | 1.991 | 4.99E-02 | 8.42E-02 |
| TG(16:0_18:1_22:1) | -0.447 | 0.469 | 2.644 | -4.688 | 1.24E-05 | 1.12E-04 |
| TG(16:0_18:1_22:4) | 0.143 | -0.15 | 0.475 | 1.362 | 1.77E-01 | 2.45E-01 |
| TG(16:0_18:1_22:6) | 0.369 | -0.388 | 0.236 | 3.752 | 3.32E-04 | 1.27E-03 |
| TG(16:0_18:1_23:0) | -0.008 | 0.009 | 1.044 | -0.079 | 9.37E-01 | 9.56E-01 |
| TG(16:0_18:1_23:1) | 0.023 | -0.024 | 0.68 | 0.219 | 8.27E-01 | 8.69E-01 |
| TG(16:0_18:1_24:0) | -0.281 | 0.295 | 1.721 | -2.734 | 7.79E-03 | 1.77E-02 |
| TG(16:0_18:1_24:1) | -0.323 | 0.34 | 2.193 | -3.189 | 2.11E-03 | 6.11E-03 |
| TG(16:0_18:2_18:2) | -0.118 | 0.124 | 3.524 | -1.095 | 2.78E-01 | 3.53E-01 |
| TG(16:0_18:2_18:3) | -0.57 | 0.599 | 4.097 | -6.607 | 4.50E-09 | 7.10E-07 |
| TG(16:0_18:2_20:4) | -0.18 | 0.189 | 2.821 | -1.701 | 9.34E-02 | 1.41E-01 |
| TG(16:0_18:2_22:6) | -0.002 | 0.002 | 0.73 | -0.016 | 9.87E-01 | 9.89E-01 |
| TG(16:0_18:3_18:3) | -0.5 | 0.525 | 2.77 | -5.483 | 4.75E-07 | 1.17E-05 |
| TG(16:0_18:3_20:4) | -0.101 | 0.106 | 0.915 | -0.945 | 3.48E-01 | 4.27E-01 |
| TG(16:0_18:3_20:5) | -0.279 | 0.293 | 2.676 | -2.708 | 8.44E-03 | 1.89E-02 |
| TG(16:0_18:3_22:6) | -0.159 | 0.167 | 2.992 | -1.492 | 1.41E-01 | 2.02E-01 |
| TG(16:0_19:0_20:4) | -0.003 | 0.003 | 0.795 | -0.027 | 9.79E-01 | 9.86E-01 |
| TG(16:0_19:0_22:0) | 0.116 | -0.122 | 0.588 | 1.1 | 2.75E-01 | 3.49E-01 |
| TG(16:0_20:4_20:5) | 0.155 | -0.162 | 0.984 | 1.459 | 1.49E-01 | 2.10E-01 |
| TG(16:0_20:4_22:6) | 0.386 | -0.405 | 0.216 | 3.951 | 1.68E-04 | 7.26E-04 |
| TG(16:0_20:4_24:0) | 0.099 | -0.104 | 0.684 | 0.93 | 3.55E-01 | 4.33E-01 |
| TG(16:0_22:1_22:6) | 0.057 | -0.059 | 1.088 | 0.523 | 6.03E-01 | 6.71E-01 |
| TG(16:0_22:3_24:2) | -0.226 | 0.237 | 2.01 | -2.154 | 3.46E-02 | 6.24E-02 |
| TG(16:0_22:6_22:6) | -0.037 | 0.038 | 2.127 | -0.339 | 7.36E-01 | 7.96E-01 |
| TG(16:0_22:6_24:0) | -0.29 | 0.304 | 2.499 | -2.829 | 5.95E-03 | 1.41E-02 |
| TG(16:0_6:0_11:1) | -0.29 | 0.304 | 2.696 | -2.827 | 5.99E-03 | 1.41E-02 |
| TG(16:0_8:0_14:0) | -0.264 | 0.277 | 1.336 | -2.575 | 1.19E-02 | 2.52E-02 |
| TG(16:0e_16:0_16:0) | -0.1 | 0.105 | 1.729 | -0.925 | 3.58E-01 | 4.35E-01 |
| TG(16:0e_16:0_18:1) | -0.072 | 0.075 | 1.624 | -0.666 | 5.08E-01 | 5.81E-01 |
| TG(16:0e_16:0_18:2) | 0.305 | -0.321 | 0.479 | 3.005 | 3.55E-03 | 9.47E-03 |
| TG(16:0e_18:0_20:1) | -0.254 | 0.267 | 3.071 | -2.439 | 1.74E-02 | 3.49E-02 |
| TG(16:0e_18:1_18:3) | 0.131 | -0.137 | 0.823 | 1.228 | 2.23E-01 | 2.95E-01 |
| TG(16:0e_18:1_20:4) | 0.111 | -0.116 | 0.797 | 1.034 | 3.04E-01 | 3.83E-01 |
| TG(16:0e_18:1_22:0) | -0.145 | 0.152 | 1.831 | -1.363 | 1.77E-01 | 2.45E-01 |
| TG(16:1_12:0_18:1) | -0.273 | 0.287 | 3.225 | -2.646 | 9.95E-03 | 2.16E-02 |
| TG(16:1_12:0_18:2) | -0.372 | 0.391 | 3.988 | -3.739 | 3.76E-04 | 1.41E-03 |
| TG(16:1_12:0_22:6) | -0.032 | 0.034 | 1.054 | -0.297 | 7.67E-01 | 8.22E-01 |
| TG(16:1_14:0_18:3) | -0.239 | 0.251 | 2.315 | -2.287 | 2.51E-02 | 4.75E-02 |
| TG(16:1_16:1_18:2) | -0.199 | 0.209 | 3.074 | -1.888 | 6.29E-02 | 1.02E-01 |
| TG(16:1_16:1_18:3) | 0.328 | -0.344 | 0.34 | 3.276 | 1.58E-03 | 4.80E-03 |
| TG(16:1_17:1_18:1) | 0.533 | -0.56 | 0.276 | 6.023 | 5.20E-08 | 3.55E-06 |
| TG(16:1_17:1_18:2) | -0.339 | 0.356 | 3.082 | -3.351 | 1.32E-03 | 4.09E-03 |
| TG(16:1_18:1_18:2) | -0.425 | 0.446 | 3.475 | -4.381 | 4.26E-05 | 2.66E-04 |
| TG(16:1_18:1_20:4) | 0.235 | -0.247 | 0.324 | 2.292 | 2.50E-02 | 4.73E-02 |
| TG(16:1_18:2_18:2) | -0.144 | 0.152 | 1.054 | -1.357 | 1.79E-01 | 2.46E-01 |
| TG(16:1_18:2_18:3) | -0.301 | 0.316 | 3.817 | -2.938 | 4.50E-03 | 1.14E-02 |
| TG(16:1_18:2_20:5) | 0.184 | -0.193 | 0.301 | 1.773 | 8.07E-02 | 1.27E-01 |
| TG(16:1_18:3_18:3) | -0.018 | 0.019 | 0.983 | -0.164 | 8.70E-01 | 9.03E-01 |
| TG(16:1_18:3_20:5) | 0.186 | -0.195 | 0.389 | 1.773 | 8.01E-02 | 1.26E-01 |
| TG(16:1_18:3_22:6) | -0.105 | 0.111 | 2.57 | -0.982 | 3.29E-01 | 4.09E-01 |
| TG(16:1_20:1_20:1) | -0.425 | 0.446 | 2.871 | -4.417 | 3.17E-05 | 2.21E-04 |
| TG(16:1_20:1_22:4) | -0.397 | 0.417 | 4.932 | -4.046 | 1.27E-04 | 5.92E-04 |
| TG(16:1_20:4_20:5) | -0.133 | 0.14 | 1.601 | -1.242 | 2.18E-01 | 2.91E-01 |
| TG(16:1_20:4_22:6) | 0.016 | -0.017 | 0.714 | 0.146 | 8.85E-01 | 9.12E-01 |
| TG(16:1_20:5_20:5) | -0.294 | 0.309 | 3.679 | -2.853 | 5.91E-03 | 1.40E-02 |
| TG(16:1e_16:0_16:0) | 0.396 | -0.416 | 0.284 | 4.089 | 1.06E-04 | 5.15E-04 |
| TG(16:1e_16:0_18:1) | 0.218 | -0.229 | 0.867 | 2.058 | 4.43E-02 | 7.63E-02 |
| TG(17:0_17:1_17:1) | -0.256 | 0.269 | 2.635 | -2.465 | 1.61E-02 | 3.27E-02 |
| TG(17:0_17:1_19:0) | 0.443 | -0.465 | 0.358 | 4.661 | 1.24E-05 | 1.12E-04 |
| TG(17:0_18:1_18:1) | 0.105 | -0.11 | 0.724 | 0.987 | 3.26E-01 | 4.06E-01 |
| TG(17:0_18:1_18:2) | 0.586 | -0.615 | 0.305 | 6.945 | 9.37E-10 | 7.10E-07 |
| TG(17:0_18:1_18:3) | 0.111 | -0.116 | 1.156 | 1.026 | 3.09E-01 | 3.88E-01 |
| TG(17:0_18:1_20:3) | -0.56 | 0.588 | 4.413 | -6.435 | 8.95E-09 | 1.18E-06 |
| TG(17:0_18:1_20:4) | 0.456 | -0.478 | 0.33 | 4.848 | 6.03E-06 | 6.73E-05 |
| TG(17:0_18:1_20:5) | -0.523 | 0.549 | 3.407 | -5.797 | 1.46E-07 | 5.50E-06 |
| TG(17:0_18:1_22:4) | 0.34 | -0.357 | 0.465 | 3.39 | 1.09E-03 | 3.49E-03 |
| TG(17:0_18:1_22:5) | 0.528 | -0.555 | 0.241 | 5.92 | 7.66E-08 | 3.80E-06 |
| TG(17:0_18:1_22:6) | -0.021 | 0.022 | 1.163 | -0.192 | 8.48E-01 | 8.84E-01 |
| TG(17:0_18:2_20:3) | 0.42 | -0.441 | 0.338 | 4.376 | 3.63E-05 | 2.37E-04 |
| TG(17:0_18:2_22:6) | 0.273 | -0.287 | 0.25 | 2.694 | 8.86E-03 | 1.96E-02 |
| TG(17:1_17:1_17:1) | -0.106 | 0.111 | 1.241 | -1.006 | 3.18E-01 | 3.98E-01 |
| TG(18:0_12:1_16:0) | 0.337 | -0.353 | 0.271 | 3.37 | 1.16E-03 | 3.66E-03 |
| TG(18:0_16:0_16:0) | -0.15 | 0.158 | 1.467 | -1.423 | 1.58E-01 | 2.23E-01 |
| TG(18:0_16:0_16:1) | 0.503 | -0.528 | 0.289 | 5.514 | 4.22E-07 | 1.11E-05 |
| TG(18:0_16:0_17:0) | -0.198 | 0.208 | 1.993 | -1.873 | 6.52E-02 | 1.05E-01 |
| TG(18:0_16:0_17:1) | -0.343 | 0.36 | 3.958 | -3.381 | 1.33E-03 | 4.13E-03 |
| TG(18:0_16:0_18:0) | 0.097 | -0.101 | 0.794 | 0.907 | 3.67E-01 | 4.42E-01 |
| TG(18:0_16:0_18:1) | -0.106 | 0.111 | 1.139 | -0.991 | 3.24E-01 | 4.04E-01 |
| TG(18:0_16:0_18:3) | -0.1 | 0.105 | 1.033 | -0.939 | 3.51E-01 | 4.29E-01 |
| TG(18:0_16:0_19:0) | -0.363 | 0.381 | 3.112 | -3.645 | 4.95E-04 | 1.77E-03 |
| TG(18:0_16:0_20:4) | 0.202 | -0.212 | 0.676 | 1.921 | 5.86E-02 | 9.62E-02 |
| TG(18:0_16:0_20:5) | 0.122 | -0.128 | 0.39 | 1.159 | 2.51E-01 | 3.25E-01 |
| TG(18:0_16:0_21:0) | -0.346 | 0.363 | 3.311 | -3.445 | 9.49E-04 | 3.08E-03 |
| TG(18:0_16:0_22:6) | -0.127 | 0.133 | 2.041 | -1.182 | 2.42E-01 | 3.16E-01 |
| TG(18:0_16:0_23:0) | -0.485 | 0.509 | 4.255 | -5.23 | 1.47E-06 | 2.51E-05 |
| TG(18:0_16:0_24:0) | -0.437 | 0.459 | 3.197 | -4.569 | 1.88E-05 | 1.50E-04 |
| TG(18:0_16:1_18:0) | -0.488 | 0.512 | 2.585 | -5.283 | 1.13E-06 | 2.17E-05 |
| TG(18:0_16:1_24:0) | -0.384 | 0.403 | 2.652 | -3.883 | 2.26E-04 | 9.16E-04 |
| TG(18:0_17:0_18:0) | -0.21 | 0.221 | 2.016 | -2.004 | 4.87E-02 | 8.23E-02 |
| TG(18:0_17:0_18:1) | 0.03 | -0.032 | 1.062 | 0.282 | 7.79E-01 | 8.32E-01 |
| TG(18:0_17:0_18:3) | 0.231 | -0.243 | 0.836 | 2.218 | 2.95E-02 | 5.45E-02 |
| TG(18:0_17:0_20:4) | 0.041 | -0.043 | 1.009 | 0.384 | 7.02E-01 | 7.64E-01 |
| TG(18:0_18:0_18:0) | -0.405 | 0.426 | 3.179 | -4.161 | 8.24E-05 | 4.30E-04 |
| TG(18:0_18:0_18:1) | 0 | 0 | 0.929 | 0.002 | 9.98E-01 | 9.98E-01 |
| TG(18:0_18:0_20:0) | -0.405 | 0.426 | 3.545 | -4.158 | 8.35E-05 | 4.31E-04 |
| TG(18:0_18:0_20:3) | 0.113 | -0.119 | 0.59 | 1.071 | 2.87E-01 | 3.63E-01 |
| TG(18:0_18:1_18:1) | -0.227 | 0.238 | 1.199 | -2.184 | 3.19E-02 | 5.80E-02 |
| TG(18:0_18:1_19:0) | -0.18 | 0.19 | 1.472 | -1.708 | 9.18E-02 | 1.39E-01 |
| TG(18:0_18:1_20:0) | -0.395 | 0.415 | 2.652 | -4.036 | 1.27E-04 | 5.91E-04 |
| TG(18:0_18:1_20:3) | 0.003 | -0.003 | 0.917 | 0.024 | 9.81E-01 | 9.87E-01 |
| TG(18:0_18:1_20:4) | -0.217 | 0.228 | 1.194 | -2.084 | 4.04E-02 | 7.15E-02 |
| TG(18:0_18:1_22:3) | -0.093 | 0.097 | 1.062 | -0.871 | 3.86E-01 | 4.60E-01 |
| TG(18:0_18:1_22:4) | -0.417 | 0.438 | 3.564 | -4.285 | 6.00E-05 | 3.43E-04 |
| TG(18:0_18:1_22:5) | -0.029 | 0.031 | 1.123 | -0.271 | 7.87E-01 | 8.36E-01 |
| TG(18:0_18:1_22:6) | 0.443 | -0.465 | 0.246 | 4.695 | 1.15E-05 | 1.05E-04 |
| TG(18:0_18:1_24:0) | -0.244 | 0.256 | 1.444 | -2.348 | 2.14E-02 | 4.17E-02 |
| TG(18:0_18:2_22:6) | -0.148 | 0.155 | 1.256 | -1.39 | 1.69E-01 | 2.36E-01 |
| TG(18:0_20:1_20:4) | -0.09 | 0.095 | 1.118 | -0.843 | 4.02E-01 | 4.75E-01 |
| TG(18:0_20:3_20:4) | 0.222 | -0.233 | 0.503 | 2.143 | 3.53E-02 | 6.32E-02 |
| TG(18:0_20:4_22:5) | 0.243 | -0.255 | 0.474 | 2.345 | 2.15E-02 | 4.18E-02 |
| TG(18:0_20:4_22:6) | -0.226 | 0.238 | 2.748 | -2.162 | 3.39E-02 | 6.12E-02 |
| TG(18:0_22:5_22:6) | -0.053 | 0.055 | 1.54 | -0.487 | 6.28E-01 | 6.96E-01 |
| TG(18:0_6:0_16:0) | 0.14 | -0.147 | 0.58 | 1.322 | 1.90E-01 | 2.59E-01 |
| TG(18:0e_16:0_16:0) | -0.084 | 0.088 | 1.752 | -0.776 | 4.41E-01 | 5.14E-01 |
| TG(18:0e_16:0_18:0) | -0.049 | 0.051 | 1.648 | -0.452 | 6.53E-01 | 7.17E-01 |
| TG(18:0e_16:0_18:1) | -0.095 | 0.1 | 1.605 | -0.886 | 3.78E-01 | 4.52E-01 |
| TG(18:0e_16:0_18:3) | 0.289 | -0.303 | 0.578 | 2.829 | 5.91E-03 | 1.40E-02 |
| TG(18:0e_18:0_18:1) | -0.071 | 0.074 | 1.666 | -0.657 | 5.13E-01 | 5.86E-01 |
| TG(18:0e_18:1_18:1) | -0.152 | 0.16 | 1.871 | -1.43 | 1.57E-01 | 2.22E-01 |
| TG(18:1_11:1_18:1) | 0.274 | -0.288 | 0.467 | 2.7 | 8.70E-03 | 1.93E-02 |
| TG(18:1_12:0_12:0) | -0.156 | 0.164 | 3.493 | -1.465 | 1.48E-01 | 2.09E-01 |
| TG(18:1_12:0_14:0) | 0.406 | -0.426 | 0.139 | 4.194 | 7.05E-05 | 3.83E-04 |
| TG(18:1_12:0_20:4) | -0.378 | 0.396 | 2.877 | -3.801 | 3.11E-04 | 1.22E-03 |
| TG(18:1_12:0_22:6) | 0.049 | -0.051 | 0.912 | 0.454 | 6.51E-01 | 7.16E-01 |
| TG(18:1_14:0_18:2) | -0.338 | 0.355 | 3.178 | -3.33 | 1.48E-03 | 4.53E-03 |
| TG(18:1_17:1_18:1) | 0.256 | -0.269 | 0.609 | 2.483 | 1.51E-02 | 3.12E-02 |
| TG(18:1_17:1_18:2) | -0.489 | 0.514 | 3.295 | -5.293 | 1.13E-06 | 2.17E-05 |
| TG(18:1_17:1_18:3) | -0.394 | 0.414 | 5.011 | -4.005 | 1.52E-04 | 6.76E-04 |
| TG(18:1_17:1_20:4) | 0.397 | -0.417 | 0.35 | 4.099 | 1.02E-04 | 5.04E-04 |
| TG(18:1_17:1_22:5) | 0.091 | -0.096 | 0.421 | 0.869 | 3.88E-01 | 4.61E-01 |
| TG(18:1_18:1_18:1) | -0.088 | 0.092 | 0.953 | -0.82 | 4.15E-01 | 4.88E-01 |
| TG(18:1_18:1_20:3) | -0.004 | 0.004 | 0.911 | -0.037 | 9.71E-01 | 9.82E-01 |
| TG(18:1_18:1_20:4) | 0.1 | -0.105 | 0.529 | 0.949 | 3.46E-01 | 4.26E-01 |
| TG(18:1_18:1_21:0) | -0.417 | 0.438 | 4.16 | -4.296 | 5.44E-05 | 3.23E-04 |
| TG(18:1_18:1_21:1) | -0.373 | 0.392 | 2.14 | -3.782 | 2.99E-04 | 1.18E-03 |
| TG(18:1_18:1_22:0) | -0.525 | 0.552 | 4.259 | -5.84 | 1.24E-07 | 4.89E-06 |
| TG(18:1_18:1_22:1) | -0.391 | 0.411 | 2.26 | -3.982 | 1.57E-04 | 6.93E-04 |
| TG(18:1_18:1_22:3) | 0.196 | -0.206 | 0.541 | 1.869 | 6.53E-02 | 1.05E-01 |
| TG(18:1_18:1_22:4) | 0.482 | -0.506 | 0.275 | 5.216 | 1.40E-06 | 2.46E-05 |
| TG(18:1_18:1_22:5) | -0.346 | 0.363 | 4.644 | -3.443 | 9.51E-04 | 3.08E-03 |
| TG(18:1_18:1_22:6) | 0.24 | -0.252 | 0.385 | 2.325 | 2.27E-02 | 4.38E-02 |
| TG(18:1_18:1_23:0) | 0.148 | -0.156 | 0.488 | 1.411 | 1.62E-01 | 2.27E-01 |
| TG(18:1_18:1_23:1) | -0.141 | 0.148 | 1.426 | -1.325 | 1.89E-01 | 2.58E-01 |
| TG(18:1_18:1_24:0) | -0.321 | 0.337 | 1.774 | -3.168 | 2.20E-03 | 6.32E-03 |
| TG(18:1_18:1_24:1) | -0.374 | 0.393 | 2.274 | -3.779 | 3.10E-04 | 1.22E-03 |
| TG(18:1_18:2_18:2) | -0.476 | 0.499 | 3.022 | -5.098 | 2.41E-06 | 3.33E-05 |
| TG(18:1_18:2_20:3) | 0.293 | -0.308 | 0.394 | 2.866 | 5.35E-03 | 1.28E-02 |
| TG(18:1_18:2_21:1) | 0.03 | -0.031 | 0.938 | 0.275 | 7.84E-01 | 8.35E-01 |
| TG(18:1_18:2_22:0) | -0.442 | 0.464 | 3.419 | -4.63 | 1.50E-05 | 1.30E-04 |
| TG(18:1_18:2_22:2) | 0.029 | -0.03 | 0.77 | 0.268 | 7.90E-01 | 8.37E-01 |
| TG(18:1_18:2_22:5) | -0.339 | 0.356 | 3.274 | -3.364 | 1.23E-03 | 3.84E-03 |
| TG(18:1_18:2_23:0) | -0.405 | 0.425 | 2.979 | -4.142 | 9.26E-05 | 4.68E-04 |
| TG(18:1_18:2_23:1) | -0.34 | 0.357 | 2.286 | -3.382 | 1.14E-03 | 3.59E-03 |
| TG(18:1_18:2_24:0) | -0.476 | 0.499 | 3.266 | -5.091 | 2.56E-06 | 3.37E-05 |
| TG(18:1_18:2_24:1) | -0.413 | 0.434 | 3.622 | -4.263 | 5.66E-05 | 3.28E-04 |
| TG(18:1_18:3_20:4) | 0.362 | -0.381 | 0.281 | 3.671 | 4.38E-04 | 1.58E-03 |
| TG(18:1_18:3_20:5) | -0.253 | 0.265 | 1.736 | -2.444 | 1.67E-02 | 3.40E-02 |
| TG(18:1_18:3_22:0) | 0.082 | -0.087 | 0.765 | 0.773 | 4.42E-01 | 5.14E-01 |
| TG(18:1_18:3_22:6) | -0.102 | 0.107 | 1.641 | -0.954 | 3.43E-01 | 4.24E-01 |
| TG(18:1_20:2_22:5) | -0.273 | 0.287 | 4.555 | -2.632 | 1.06E-02 | 2.28E-02 |
| TG(18:1_20:3_20:3) | 0.268 | -0.281 | 0.352 | 2.61 | 1.08E-02 | 2.31E-02 |
| TG(18:1_20:3_20:4) | 0.098 | -0.103 | 0.391 | 0.93 | 3.55E-01 | 4.33E-01 |
| TG(18:1_20:3_22:5) | -0.073 | 0.076 | 0.862 | -0.681 | 4.98E-01 | 5.70E-01 |
| TG(18:1_20:3_22:6) | -0.108 | 0.113 | 1.495 | -1.003 | 3.19E-01 | 3.99E-01 |
| TG(18:1_20:4_20:5) | 0.161 | -0.169 | 0.295 | 1.54 | 1.28E-01 | 1.87E-01 |
| TG(18:1_20:4_22:0) | 0.029 | -0.031 | 0.92 | 0.273 | 7.86E-01 | 8.35E-01 |
| TG(18:1_20:4_22:1) | -0.101 | 0.106 | 1.257 | -0.944 | 3.48E-01 | 4.27E-01 |
| TG(18:1_20:4_22:5) | 0.262 | -0.275 | 0.37 | 2.547 | 1.28E-02 | 2.69E-02 |
| TG(18:1_20:4_24:0) | 0.055 | -0.058 | 0.772 | 0.515 | 6.08E-01 | 6.76E-01 |
| TG(18:1_20:5_20:5) | -0.044 | 0.046 | 1.833 | -0.41 | 6.83E-01 | 7.46E-01 |
| TG(18:1_20:5_22:0) | -0.065 | 0.068 | 1.075 | -0.606 | 5.46E-01 | 6.18E-01 |
| TG(18:1_20:5_22:5) | 0.209 | -0.219 | 0.449 | 2.009 | 4.80E-02 | 8.14E-02 |
| TG(18:1_20:5_24:0) | 0.014 | -0.015 | 0.879 | 0.134 | 8.94E-01 | 9.18E-01 |
| TG(18:1_22:4_22:6) | -0.192 | 0.202 | 1.687 | -1.831 | 7.09E-02 | 1.13E-01 |
| TG(18:1_22:5_22:5) | 0.103 | -0.108 | 0.654 | 0.965 | 3.38E-01 | 4.18E-01 |
| TG(18:1_22:5_22:6) | 0.31 | -0.325 | 0.347 | 3.064 | 2.98E-03 | 8.21E-03 |
| TG(18:1e_16:0_18:1) | -0.19 | 0.199 | 2.46 | -1.792 | 7.77E-02 | 1.23E-01 |
| TG(18:1e_16:0_18:2) | 0.334 | -0.35 | 0.427 | 3.324 | 1.34E-03 | 4.14E-03 |
| TG(18:1e_18:1_18:1) | -0.169 | 0.178 | 1.902 | -1.59 | 1.16E-01 | 1.72E-01 |
| TG(18:2_17:1_18:2) | -0.017 | 0.017 | 0.958 | -0.154 | 8.78E-01 | 9.09E-01 |
| TG(18:2_18:2_18:2) | 0.131 | -0.137 | 0.26 | 1.243 | 2.18E-01 | 2.91E-01 |
| TG(18:2_18:2_22:6) | -0.098 | 0.103 | 1.305 | -0.916 | 3.62E-01 | 4.38E-01 |
| TG(18:2_20:4_22:6) | -0.297 | 0.312 | 3.6 | -2.9 | 4.95E-03 | 1.21E-02 |
| TG(18:2e_16:0_18:1) | 0.165 | -0.173 | 1.03 | 1.541 | 1.29E-01 | 1.88E-01 |
| TG(18:2e_18:1_22:0) | -0.22 | 0.231 | 1.993 | -2.098 | 3.94E-02 | 7.00E-02 |
| TG(18:3_14:1_18:2) | -0.241 | 0.254 | 2.512 | -2.318 | 2.31E-02 | 4.42E-02 |
| TG(18:3_14:1_18:3) | -0.249 | 0.262 | 3.991 | -2.388 | 1.98E-02 | 3.90E-02 |
| TG(18:3_17:1_18:2) | -0.172 | 0.18 | 1.872 | -1.611 | 1.12E-01 | 1.67E-01 |
| TG(18:3_18:2_18:2) | -0.184 | 0.194 | 1.667 | -1.744 | 8.53E-02 | 1.32E-01 |
| TG(18:3_18:2_18:3) | 0.085 | -0.089 | 0.591 | 0.8 | 4.26E-01 | 4.99E-01 |
| TG(18:3_18:2_20:5) | 0.234 | -0.246 | 0.367 | 2.262 | 2.64E-02 | 4.96E-02 |
| TG(18:3_18:2_22:5) | -0.409 | 0.43 | 3.535 | -4.207 | 7.08E-05 | 3.83E-04 |
| TG(18:3_18:2_22:6) | -0.178 | 0.187 | 1.604 | -1.691 | 9.48E-02 | 1.43E-01 |
| TG(18:3_18:3_18:3) | -0.063 | 0.066 | 2.107 | -0.581 | 5.63E-01 | 6.36E-01 |
| TG(18:3_18:3_22:6) | -0.002 | 0.002 | 1.151 | -0.018 | 9.86E-01 | 9.89E-01 |
| TG(18:3_20:5_20:5) | -0.111 | 0.117 | 1.899 | -1.042 | 3.01E-01 | 3.79E-01 |
| TG(18:3_20:5_22:6) | -0.045 | 0.047 | 1.465 | -0.418 | 6.77E-01 | 7.41E-01 |
| TG(18:3_22:6_22:6) | 0.004 | -0.004 | 2.022 | 0.032 | 9.74E-01 | 9.83E-01 |
| TG(18:4_14:0_16:1) | -0.304 | 0.32 | 2.808 | -2.968 | 4.15E-03 | 1.07E-02 |
| TG(18:4_14:0_18:2) | -0.038 | 0.04 | 1.296 | -0.352 | 7.26E-01 | 7.87E-01 |
| TG(18:4_14:0_20:4) | 0.061 | -0.064 | 0.912 | 0.573 | 5.68E-01 | 6.40E-01 |
| TG(18:4_16:0_16:1) | -0.324 | 0.34 | 3.4 | -3.187 | 2.16E-03 | 6.25E-03 |
| TG(18:4_16:0_18:3) | -0.234 | 0.246 | 1.652 | -2.235 | 2.88E-02 | 5.38E-02 |
| TG(18:4_16:0_20:4) | -0.263 | 0.276 | 4.341 | -2.521 | 1.44E-02 | 2.99E-02 |
| TG(18:4_16:1_18:2) | -0.477 | 0.501 | 2.627 | -5.107 | 2.48E-06 | 3.37E-05 |
| TG(18:4_16:1_18:3) | 0.415 | -0.436 | 0.216 | 4.332 | 4.42E-05 | 2.69E-04 |
| TG(18:4_16:1_22:6) | 0.121 | -0.127 | 0.591 | 1.133 | 2.61E-01 | 3.35E-01 |
| TG(18:4_18:1_18:2) | -0.123 | 0.129 | 1.475 | -1.155 | 2.52E-01 | 3.26E-01 |
| TG(18:4_18:1_20:4) | -0.387 | 0.407 | 3.488 | -3.935 | 1.85E-04 | 7.82E-04 |
| TG(18:4_18:2_18:2) | -0.231 | 0.243 | 4.19 | -2.204 | 3.09E-02 | 5.66E-02 |
| TG(19:0_18:1_18:1) | -0.539 | 0.566 | 3.267 | -6.074 | 4.36E-08 | 3.55E-06 |
| TG(19:0_18:1_20:4) | -0.027 | 0.028 | 1.031 | -0.252 | 8.02E-01 | 8.49E-01 |
| TG(19:0_18:2_18:2) | 0.347 | -0.364 | 0.371 | 3.489 | 7.96E-04 | 2.67E-03 |
| TG(19:1_18:0_18:1) | -0.342 | 0.36 | 2.657 | -3.401 | 1.09E-03 | 3.49E-03 |
| TG(19:1_18:1_18:2) | 0.431 | -0.453 | 0.24 | 4.532 | 2.06E-05 | 1.61E-04 |
| TG(19:1_18:1_20:4) | 0.346 | -0.364 | 0.364 | 3.472 | 8.37E-04 | 2.80E-03 |
| TG(19:1_18:2_18:2) | -0.1 | 0.105 | 2.667 | -0.925 | 3.59E-01 | 4.35E-01 |
| TG(20:0_16:0_18:1) | -0.465 | 0.488 | 2.925 | -4.951 | 4.23E-06 | 5.06E-05 |
| TG(20:0_16:0_24:1) | -0.411 | 0.431 | 2.653 | -4.225 | 6.72E-05 | 3.71E-04 |
| TG(20:0_18:1_18:1) | -0.513 | 0.538 | 3.137 | -5.65 | 2.55E-07 | 8.37E-06 |
| TG(20:0_18:1_20:4) | -0.297 | 0.312 | 1.496 | -2.904 | 4.83E-03 | 1.20E-02 |
| TG(20:0_18:1_22:6) | 0.021 | -0.022 | 0.9 | 0.2 | 8.42E-01 | 8.79E-01 |
| TG(20:0_18:2_18:2) | -0.507 | 0.532 | 3.929 | -5.568 | 3.46E-07 | 1.05E-05 |
| TG(20:0_18:2_22:6) | -0.296 | 0.31 | 1.821 | -2.897 | 4.88E-03 | 1.20E-02 |
| TG(20:0_19:0_22:3) | -0.086 | 0.09 | 0.997 | -0.805 | 4.23E-01 | 4.96E-01 |
| TG(20:0e_16:0_18:0) | -0.039 | 0.04 | 1.727 | -0.357 | 7.22E-01 | 7.84E-01 |
| TG(20:0e_18:1_18:1) | -0.17 | 0.178 | 1.932 | -1.604 | 1.13E-01 | 1.67E-01 |
| TG(20:0e_18:1_18:2) | -0.098 | 0.102 | 2.097 | -0.907 | 3.68E-01 | 4.42E-01 |
| TG(20:0e_18:1_20:3) | -0.233 | 0.245 | 2.134 | -2.224 | 2.94E-02 | 5.45E-02 |
| TG(20:0e_18:1_22:1) | -0.211 | 0.221 | 1.995 | -2.005 | 4.86E-02 | 8.22E-02 |
| TG(20:1_14:1_22:4) | 0.301 | -0.316 | 0.234 | 2.987 | 3.82E-03 | 1.01E-02 |
| TG(20:1_18:1_20:4) | -0.033 | 0.035 | 0.786 | -0.306 | 7.61E-01 | 8.18E-01 |
| TG(20:1_18:1_22:6) | 0.321 | -0.337 | 0.277 | 3.208 | 1.96E-03 | 5.72E-03 |
| TG(20:1_20:4_20:4) | 0.296 | -0.31 | 0.329 | 2.91 | 4.68E-03 | 1.17E-02 |
| TG(20:1_20:4_20:5) | -0.257 | 0.27 | 4.231 | -2.476 | 1.56E-02 | 3.18E-02 |
| TG(20:1e_19:0_19:0) | -0.215 | 0.226 | 1.837 | -2.054 | 4.33E-02 | 7.52E-02 |
| TG(20:2_20:4_20:4) | 0.068 | -0.071 | 1.062 | 0.63 | 5.30E-01 | 6.04E-01 |
| TG(20:2e_19:0_19:0) | -0.181 | 0.19 | 1.896 | -1.707 | 9.19E-02 | 1.39E-01 |
| TG(20:3_18:2_18:2) | 0.313 | -0.329 | 0.358 | 3.112 | 2.60E-03 | 7.28E-03 |
| TG(20:5_14:1_18:2) | -0.317 | 0.333 | 2.607 | -3.125 | 2.53E-03 | 7.15E-03 |
| TG(20:5_18:2_20:4) | 0.198 | -0.208 | 0.21 | 1.912 | 6.03E-02 | 9.90E-02 |
| TG(20:5_18:2_20:5) | 0.023 | -0.024 | 0.525 | 0.214 | 8.31E-01 | 8.70E-01 |
| TG(20:5_18:2_22:6) | -0.145 | 0.152 | 2.551 | -1.361 | 1.78E-01 | 2.46E-01 |
| TG(20:5_20:4_20:4) | -0.168 | 0.176 | 2.641 | -1.576 | 1.20E-01 | 1.76E-01 |
| TG(22:2_14:1_14:1) | 0.357 | -0.375 | 0.273 | 3.604 | 5.42E-04 | 1.90E-03 |
| TG(22:5_17:1_18:2) | -0.161 | 0.169 | 2.715 | -1.508 | 1.36E-01 | 1.97E-01 |
| TG(22:5_18:2_18:2) | -0.242 | 0.254 | 2.757 | -2.326 | 2.27E-02 | 4.38E-02 |
| TG(22:5_18:2_22:5) | -0.096 | 0.101 | 3.267 | -0.89 | 3.77E-01 | 4.50E-01 |
| TG(25:0_16:0_16:0) | 0.333 | -0.349 | 0.466 | 3.316 | 1.37E-03 | 4.22E-03 |
| TG(25:0_16:0_18:0) | -0.199 | 0.209 | 1.587 | -1.888 | 6.29E-02 | 1.02E-01 |
| TG(25:0_16:0_18:1) | 0.058 | -0.061 | 0.911 | 0.543 | 5.89E-01 | 6.59E-01 |
| TG(25:0_18:1_18:1) | -0.035 | 0.037 | 0.992 | -0.329 | 7.43E-01 | 8.02E-01 |
| TG(25:0_18:1_18:2) | -0.049 | 0.052 | 0.842 | -0.463 | 6.45E-01 | 7.12E-01 |
| TG(26:0_16:0_16:0) | -0.323 | 0.339 | 1.716 | -3.216 | 1.88E-03 | 5.57E-03 |
| TG(26:0_16:0_18:0) | -0.216 | 0.227 | 1.716 | -2.058 | 4.32E-02 | 7.51E-02 |
| TG(26:0_16:0_22:6) | 0.024 | -0.025 | 1.176 | 0.22 | 8.26E-01 | 8.69E-01 |
| TG(26:0_18:1_18:1) | -0.308 | 0.324 | 1.739 | -3.033 | 3.29E-03 | 8.88E-03 |
| TG(26:0_18:1_18:2) | -0.433 | 0.454 | 3.036 | -4.505 | 2.44E-05 | 1.83E-04 |
| TG(26:1_18:1_18:1) | -0.361 | 0.379 | 2.194 | -3.632 | 5.05E-04 | 1.80E-03 |
| TG(26:1_18:1_18:2) | -0.392 | 0.412 | 3.339 | -3.979 | 1.71E-04 | 7.28E-04 |
| TG(27:0_16:0_18:1) | -0.062 | 0.065 | 1.231 | -0.574 | 5.68E-01 | 6.40E-01 |
| TG(28:0_16:0_18:1) | -0.249 | 0.262 | 1.614 | -2.406 | 1.85E-02 | 3.69E-02 |
| TG(28:0_18:1_18:1) | -0.362 | 0.38 | 2.295 | -3.63 | 5.22E-04 | 1.85E-03 |
| TG(29:0_14:0_16:0) | -0.194 | 0.204 | 1.443 | -1.839 | 7.00E-02 | 1.12E-01 |
| TG(29:0_16:0_16:0) | -0.291 | 0.305 | 1.611 | -2.833 | 5.94E-03 | 1.41E-02 |
| TG(29:0_16:0_18:1) | -0.172 | 0.181 | 1.85 | -1.617 | 1.11E-01 | 1.65E-01 |
| TG(29:0_16:0_18:2) | -0.12 | 0.126 | 1.591 | -1.113 | 2.70E-01 | 3.44E-01 |
| TG(29:0_18:0_18:1) | -0.135 | 0.141 | 2.423 | -1.255 | 2.14E-01 | 2.88E-01 |
| TG(29:0_18:1_18:1) | -0.295 | 0.309 | 2.156 | -2.876 | 5.25E-03 | 1.27E-02 |
| TG(29:0_18:1_18:2) | -0.237 | 0.249 | 2.339 | -2.261 | 2.70E-02 | 5.07E-02 |
| TG(30:0_16:0_18:1) | -0.275 | 0.289 | 1.772 | -2.673 | 9.16E-03 | 2.01E-02 |
| TG(30:0_18:1_18:1) | -0.181 | 0.19 | 1.673 | -1.71 | 9.17E-02 | 1.39E-01 |
| TG(31:2e) | -0.095 | 0.1 | 1.084 | -0.901 | 3.71E-01 | 4.44E-01 |
| TG(33:2) | -0.064 | 0.067 | 0.411 | -0.608 | 5.45E-01 | 6.18E-01 |
| TG(33:4e) | -0.329 | 0.345 | 2.639 | -3.257 | 1.67E-03 | 5.04E-03 |
| TG(4:0_16:0_20:5) | -0.002 | 0.002 | 0.652 | -0.02 | 9.84E-01 | 9.89E-01 |
| TG(50:5) | -0.174 | 0.183 | 2.824 | -1.638 | 1.06E-01 | 1.58E-01 |
| TG(51:3) | -0.412 | 0.433 | 2.367 | -4.228 | 7.26E-05 | 3.85E-04 |
| TG(53:4) | -0.448 | 0.47 | 4.186 | -4.694 | 1.27E-05 | 1.13E-04 |
| TG(55:5) | 0.368 | -0.386 | 0.241 | 3.748 | 3.51E-04 | 1.33E-03 |
| TG(56:7) | -0.299 | 0.314 | 4.112 | -2.915 | 4.77E-03 | 1.19E-02 |
| TG(57:6) | 0.122 | -0.128 | 0.884 | 1.145 | 2.56E-01 | 3.30E-01 |
| TG(58:5) | 0.004 | -0.004 | 0.655 | 0.036 | 9.71E-01 | 9.82E-01 |
| TG(58:9) | -0.168 | 0.177 | 2.951 | -1.583 | 1.18E-01 | 1.73E-01 |
| TG(6:0_11:1_14:2) | -0.186 | 0.195 | 2.73 | -1.754 | 8.39E-02 | 1.31E-01 |
| TG(6:0_11:1_14:3) | -0.223 | 0.234 | 1.098 | -2.147 | 3.48E-02 | 6.25E-02 |
| TG(6:0_11:1_18:2) | 0.151 | -0.158 | 0.269 | 1.436 | 1.55E-01 | 2.19E-01 |
| TG(6:0_11:1_18:3) | -0.439 | 0.461 | 4.549 | -4.597 | 1.67E-05 | 1.39E-04 |
| TG(6:0_11:2_18:2) | 0.078 | -0.082 | 0.388 | 0.737 | 4.63E-01 | 5.37E-01 |
| TG(6:0_11:2_18:3) | -0.305 | 0.32 | 3.427 | -2.975 | 4.07E-03 | 1.06E-02 |
| TG(6:0_6:0_17:1) | 0.029 | -0.031 | 0.254 | 0.277 | 7.83E-01 | 8.35E-01 |
| TG(6:0_6:0_21:1) | 0.18 | -0.189 | 0.231 | 1.737 | 8.70E-02 | 1.34E-01 |
| TG(6:0_6:0_23:0) | -0.032 | 0.034 | 0.591 | -0.303 | 7.63E-01 | 8.19E-01 |
| TG(60:10) | -0.221 | 0.232 | 4.735 | -2.099 | 3.98E-02 | 7.06E-02 |
| TG(60:3e) | -0.229 | 0.241 | 2.106 | -2.19 | 3.18E-02 | 5.79E-02 |
| TG(60:4e) | -0.273 | 0.287 | 2.365 | -2.638 | 1.03E-02 | 2.23E-02 |
| TG(60:6) | -0.35 | 0.367 | 1.944 | -3.484 | 8.47E-04 | 2.82E-03 |
| TG(67:2) | -0.367 | 0.385 | 2.694 | -3.691 | 4.16E-04 | 1.53E-03 |

Residuals were obtained adjusted for age, sex, BMI, diabetes status, hypertension status, medication status for hypertension and hyperlipidemia, APOE e4 carrier status, education and current smoking status.

Fold changes were calculated by diving original abundance of lipids in AD to that in Control group.

Cer – ceramide; PC – phosphatidylcholines; PE – phosphatidylethanolamines; PI - phosphatidylinositol (PI); LPC - lyso-phatidylcholines; ChE - cholesterol esters, DG – diacylglycerol; TG – triacylglycerols; SM – sphingomyelin
