# Supplementary material for: Plasma lipidome is dysregulated in Alzheimer’s disease and is associated with disease risk genes
Source: Transl Psychiatry. 2021 Jun 7;11:344. doi: 10.1038/s41398-021-01362-2 (PMC8180517; doi:10.1038/s41398-021-01362-2)
Supplement: Supplementary file 3 — Supplementary table 3. Comparison of residuals of group lipids in AD and Control group [file 41398_2021_1362_MOESM3_ESM.docx]

**Supplementary table 3. Comparison of residuals of group lipids in AD and Control group**

| **Lipid groups** | **Control** | | **AD** | | **t statistics** | **p value** |
| --- | --- | --- | --- | --- | --- | --- |
|  | **Mean (residuals)** | **SE** | **Mean (residuals)** | **SE** |  |  |
| **Group Cer** | 0.080 | 0.160 | -0.084 | 0.149 | 0.747 | 0.457 |
| **Group SM** | 0.133 | 0.137 | -0.140 | 0.170 | 1.256 | 0.213 |
| **SM/Cer** | 0.176 | 0.145 | -0.185 | 0.161 | 1.673 | 0.098 |
| **Group LPC** | 0.104 | 0.128 | -0.109 | 0.178 | 0.977 | 0.332 |
| **Group PC** | 0.169 | 0.132 | -0.177 | 0.173 | 1.600 | 0.114 |
| **LPC/PC** | 0.091 | 0.121 | -0.096 | 0.184 | 0.849 | 0.399 |
| **Group PE** | 0.061 | 0.147 | -0.064 | 0.163 | 0.567 | 0.573 |
| **Group PI** | -0.157 | 0.136 | 0.165 | 0.170 | -1.484 | 0.142 |
| **Group ChE** | 0.156 | 0.151 | -0.163 | 0.156 | 1.473 | 0.145 |
| **Group DG** | -0.276 | 0.143 | 0.290 | 0.154 | -2.696 | 0.009 |
| **Group TG** | -0.185 | 0.139 | 0.195 | 0.166 | -1.764 | 0.082 |

Residuals were obtained by regressing out original abundance of lipids for age, sex, BMI, diabetes status, hypertension status, medication status for hypertension and hyperlipidemia, APOE e4 carrier status, education and current smoking status.

SE- standard error; Cer – ceramide; PC – phosphatidylcholines; PE – phosphatidylethanolamines; PI - phosphatidylinositol (PI); LPC - lyso-phatidylcholines; ChE - cholesterol esters, DG – diacylglycerol; TG – triacylglycerols; SM – sphingomyelin
