# Supplementary material for: Plasma lipidome is dysregulated in Alzheimer’s disease and is associated with disease risk genes
Source: Transl Psychiatry. 2021 Jun 7;11:344. doi: 10.1038/s41398-021-01362-2 (PMC8180517; doi:10.1038/s41398-021-01362-2)
Supplement: Supplementary file 4 — Supplementary table 4. Differential association of the AD risk PRS scores with lipids between AD and controls [file 41398_2021_1362_MOESM4_ESM.docx]

**Supplementary table 4. Differential association of the AD risk PRS scores with lipids between AD and controls**

| **Lipid** | **Beta.PRS** | **Beta.PRS_CC** | **SE.PRS** | **SE.PRS_CC** | **tvalue.CC** | **tvalue.PRS_CC** | **Pval.PRS** | **Pval.PRS_CC** | **Adjusted Pval.PRS_CC** |
| --- | --- | --- | --- | --- | --- | --- | --- | --- | --- |
| **Cer(d16:0_24:1)** | -0.042 | 0.192 | 0.150 | 0.220 | 2.382 | 0.875 | 0.781 | 0.384 | 0.795 |
| **Cer(d16:1_16:0)** | 0.153 | -0.245 | 0.149 | 0.218 | 2.886 | -1.125 | 0.309 | 0.264 | 0.694 |
| **Cer(d16:1_22:0)** | 0.013 | -0.337 | 0.134 | 0.196 | -5.148 | -1.720 | 0.921 | 0.090 | 0.409 |
| **Cer(d16:1_23:0)** | 0.000 | -0.275 | 0.140 | 0.205 | -4.331 | -1.338 | 0.997 | 0.185 | 0.589 |
| **Cer(d18:0_16:0)** | 0.113 | 0.248 | 0.141 | 0.206 | 3.651 | 1.205 | 0.424 | 0.232 | 0.651 |
| **Cer(d18:0_18:0)** | 0.124 | 0.219 | 0.140 | 0.206 | 3.070 | 1.065 | 0.380 | 0.290 | 0.721 |
| **Cer(d18:0_22:0)** | 0.094 | 0.081 | 0.145 | 0.212 | 3.841 | 0.382 | 0.516 | 0.704 | 0.927 |
| **Cer(d18:0_23:0)** | 0.098 | 0.083 | 0.138 | 0.202 | 4.722 | 0.409 | 0.478 | 0.684 | 0.927 |
| **Cer(d18:0_24:0)** | 0.168 | -0.051 | 0.145 | 0.212 | 3.904 | -0.242 | 0.248 | 0.809 | 0.949 |
| **Cer(d18:0_24:1)** | 0.127 | 0.004 | 0.147 | 0.215 | 3.383 | 0.017 | 0.389 | 0.987 | 0.994 |
| **Cer(d18:1_16:0)** | 0.197 | -0.511 | 0.133 | 0.195 | -5.064 | -2.624 | 0.142 | 0.011 | 0.275 |
| **Cer(d18:1_18:0)** | -0.007 | -0.092 | 0.139 | 0.204 | -4.943 | -0.452 | 0.958 | 0.652 | 0.917 |
| **Cer(d18:1_22:0)** | -0.026 | -0.318 | 0.130 | 0.190 | -5.587 | -1.672 | 0.843 | 0.099 | 0.431 |
| **Cer(d18:1_23:0)** | -0.020 | -0.290 | 0.130 | 0.190 | -5.754 | -1.526 | 0.876 | 0.131 | 0.483 |
| **Cer(d18:1_24:0)** | -0.023 | -0.283 | 0.136 | 0.200 | -4.764 | -1.415 | 0.865 | 0.161 | 0.537 |
| **Cer(d18:1_25:0)** | 0.085 | 0.069 | 0.147 | 0.215 | 3.575 | 0.318 | 0.564 | 0.751 | 0.944 |
| **Cer(d18:1_26:1)** | 0.009 | -0.283 | 0.139 | 0.204 | -4.544 | -1.388 | 0.947 | 0.169 | 0.553 |
| **Cer(d18:2_24:0)** | 0.152 | 0.073 | 0.139 | 0.203 | 4.445 | 0.360 | 0.277 | 0.720 | 0.927 |
| **Cer(d18:2_24:1)** | 0.127 | 0.138 | 0.141 | 0.207 | 3.766 | 0.665 | 0.373 | 0.508 | 0.874 |
| **Cer(d18:2_25:0)** | 0.118 | 0.072 | 0.138 | 0.203 | 4.506 | 0.357 | 0.397 | 0.722 | 0.927 |
| **Cer(d19:0_23:0)** | 0.105 | 0.075 | 0.145 | 0.212 | 3.888 | 0.353 | 0.470 | 0.725 | 0.929 |
| **Cer(d19:1_22:0)** | -0.079 | -0.151 | 0.138 | 0.203 | -4.634 | -0.743 | 0.569 | 0.460 | 0.848 |
| **Cer(d19:1_24:0)** | 0.123 | -0.045 | 0.140 | 0.205 | 4.361 | -0.217 | 0.385 | 0.829 | 0.951 |
| **Cer(d19:1_24:1)** | 0.215 | -0.117 | 0.142 | 0.209 | 3.042 | -0.563 | 0.135 | 0.575 | 0.890 |
| **Cer(d38:1)** | 0.098 | -0.122 | 0.139 | 0.204 | 4.470 | -0.598 | 0.483 | 0.552 | 0.874 |
| **Cer(m18:0_20:0)** | -0.119 | -0.146 | 0.140 | 0.205 | -4.331 | -0.710 | 0.397 | 0.480 | 0.855 |
| **Cer(m18:0_22:0)** | -0.132 | -0.155 | 0.135 | 0.198 | -4.773 | -0.782 | 0.333 | 0.437 | 0.844 |
| **Cer(m18:0_24:0)** | -0.172 | -0.124 | 0.143 | 0.210 | -3.434 | -0.593 | 0.232 | 0.555 | 0.876 |
| **Cer(m18:0_24:1)** | -0.304 | -0.031 | 0.133 | 0.195 | -4.369 | -0.157 | 0.025 | 0.876 | 0.962 |
| **Cer(m18:1_20:0)** | -0.165 | -0.144 | 0.129 | 0.189 | -5.426 | -0.765 | 0.204 | 0.447 | 0.844 |
| **Cer(m18:1_22:0)** | -0.097 | 0.401 | 0.140 | 0.205 | 3.251 | 1.954 | 0.489 | 0.055 | 0.367 |
| **Cer(m18:1_23:0)** | -0.066 | -0.190 | 0.143 | 0.209 | -3.966 | -0.907 | 0.644 | 0.368 | 0.791 |
| **Cer(m18:1_24:0)** | -0.061 | -0.054 | 0.151 | 0.221 | -2.628 | -0.244 | 0.689 | 0.808 | 0.949 |
| **Cer(m18:1_24:1)** | -0.025 | -0.258 | 0.136 | 0.199 | -4.924 | -1.298 | 0.852 | 0.198 | 0.599 |
| **Cer(t16:1_14:0)** | 0.268 | -0.510 | 0.141 | 0.207 | -3.958 | -2.461 | 0.062 | 0.016 | 0.279 |
| **ChE(16:0)** | 0.078 | 0.307 | 0.120 | 0.176 | 6.452 | 1.743 | 0.520 | 0.085 | 0.408 |
| **ChE(17:0)** | -0.161 | 0.082 | 0.143 | 0.209 | -4.082 | 0.394 | 0.265 | 0.695 | 0.927 |
| **ChE(18:0)** | 0.003 | -0.057 | 0.152 | 0.223 | -3.030 | -0.257 | 0.984 | 0.798 | 0.949 |
| **ChE(18:2)** | 0.066 | -0.372 | 0.146 | 0.213 | -3.227 | -1.742 | 0.654 | 0.086 | 0.408 |
| **ChE(18:3)** | -0.086 | -0.148 | 0.130 | 0.191 | -5.835 | -0.776 | 0.510 | 0.440 | 0.844 |
| **ChE(20:1)** | -0.047 | 0.372 | 0.148 | 0.216 | 2.998 | 1.722 | 0.750 | 0.089 | 0.409 |
| **ChE(20:2)** | 0.092 | -0.158 | 0.147 | 0.215 | -3.710 | -0.734 | 0.536 | 0.465 | 0.848 |
| **ChE(20:3)** | -0.154 | -0.063 | 0.133 | 0.194 | -5.311 | -0.324 | 0.251 | 0.747 | 0.942 |
| **ChE(22:3)** | 0.046 | 0.045 | 0.138 | 0.202 | 4.793 | 0.224 | 0.738 | 0.823 | 0.950 |
| **ChE(22:4)** | -0.063 | -0.123 | 0.138 | 0.202 | -4.871 | -0.608 | 0.650 | 0.545 | 0.874 |
| **ChE(22:5)** | -0.080 | 0.539 | 0.137 | 0.200 | 2.974 | 2.690 | 0.562 | 0.009 | 0.275 |
| **ChE(22:6)** | 0.170 | 0.325 | 0.122 | 0.179 | 4.143 | 1.821 | 0.166 | 0.073 | 0.377 |
| **ChE(23:1)** | -0.166 | 0.081 | 0.140 | 0.205 | -4.664 | 0.395 | 0.238 | 0.694 | 0.927 |
| **DG()** | 0.212 | -0.018 | 0.149 | 0.218 | 2.569 | -0.082 | 0.160 | 0.934 | 0.974 |
| **DG(16:0_16:0)** | 0.166 | -0.358 | 0.151 | 0.221 | -2.267 | -1.618 | 0.275 | 0.110 | 0.439 |
| **DG(16:0_16:1)** | -0.029 | 0.235 | 0.144 | 0.211 | 3.414 | 1.112 | 0.843 | 0.270 | 0.694 |
| **DG(16:0_18:1)** | -0.045 | -0.091 | 0.146 | 0.214 | -3.794 | -0.426 | 0.759 | 0.672 | 0.921 |
| **DG(16:0_18:3)** | 0.020 | 0.315 | 0.130 | 0.191 | 4.896 | 1.653 | 0.879 | 0.103 | 0.431 |
| **DG(16:0_20:4)** | 0.000 | 0.227 | 0.142 | 0.208 | 4.142 | 1.092 | 0.997 | 0.279 | 0.705 |
| **DG(16:1_18:1)** | 0.161 | 0.098 | 0.140 | 0.206 | 3.537 | 0.478 | 0.256 | 0.634 | 0.912 |
| **DG(16:1_18:2)** | 0.068 | 0.022 | 0.138 | 0.201 | 3.458 | 0.111 | 0.621 | 0.912 | 0.971 |
| **DG(17:0_18:1)** | 0.175 | 0.011 | 0.137 | 0.201 | 4.286 | 0.055 | 0.206 | 0.957 | 0.987 |
| **DG(17:1_18:1)** | 0.193 | -0.004 | 0.139 | 0.203 | 4.259 | -0.021 | 0.168 | 0.983 | 0.994 |
| **DG(18:0_18:0)** | 0.206 | -0.073 | 0.137 | 0.201 | 4.402 | -0.362 | 0.138 | 0.719 | 0.927 |
| **DG(18:0_18:1)** | 0.026 | -0.119 | 0.148 | 0.216 | -3.738 | -0.551 | 0.862 | 0.583 | 0.890 |
| **DG(18:1_14:0)** | -0.242 | 0.066 | 0.149 | 0.218 | -2.615 | 0.306 | 0.108 | 0.761 | 0.948 |
| **DG(18:1_18:1)** | 0.225 | 0.066 | 0.132 | 0.193 | 4.465 | 0.342 | 0.092 | 0.733 | 0.933 |
| **DG(18:1_18:3)** | 0.115 | 0.277 | 0.139 | 0.204 | 2.626 | 1.357 | 0.411 | 0.179 | 0.573 |
| **DG(18:1_20:3)** | 0.219 | -0.009 | 0.142 | 0.208 | 3.791 | -0.043 | 0.127 | 0.966 | 0.988 |
| **DG(18:1_20:4)** | 0.103 | -0.204 | 0.152 | 0.222 | -2.945 | -0.916 | 0.500 | 0.363 | 0.791 |
| **DG(18:1_22:5)** | 0.294 | -0.002 | 0.138 | 0.202 | 2.926 | -0.011 | 0.036 | 0.991 | 0.996 |
| **DG(18:2_18:2)** | 0.292 | -0.039 | 0.135 | 0.198 | 3.169 | -0.199 | 0.034 | 0.843 | 0.956 |
| **DG(20:0_18:1)** | 0.165 | 0.098 | 0.129 | 0.189 | 5.511 | 0.520 | 0.205 | 0.605 | 0.902 |
| **DG(20:0_18:2)** | 0.252 | 0.015 | 0.120 | 0.176 | 5.707 | 0.084 | 0.040 | 0.933 | 0.974 |
| **DG(20:1_18:2)** | 0.139 | 0.064 | 0.132 | 0.194 | 4.712 | 0.330 | 0.296 | 0.743 | 0.939 |
| **DG(20:5_18:2)** | 0.209 | 0.125 | 0.131 | 0.192 | 3.710 | 0.653 | 0.114 | 0.516 | 0.874 |
| **DG(22:4e)** | 0.243 | -0.061 | 0.142 | 0.209 | 3.096 | -0.292 | 0.092 | 0.771 | 0.949 |
| **DG(30:2e)** | 0.187 | -0.007 | 0.133 | 0.195 | 5.087 | -0.038 | 0.163 | 0.970 | 0.989 |
| **DG(32:2e)** | -0.042 | 0.181 | 0.135 | 0.198 | 5.194 | 0.914 | 0.757 | 0.364 | 0.791 |
| **DG(34:1e)** | 0.220 | -0.052 | 0.130 | 0.190 | 5.393 | -0.271 | 0.094 | 0.787 | 0.949 |
| **DG(34:4e)** | 0.305 | -0.084 | 0.135 | 0.198 | 4.375 | -0.423 | 0.027 | 0.673 | 0.921 |
| **DG(35:3e)** | 0.145 | -0.406 | 0.152 | 0.222 | -2.178 | -1.829 | 0.342 | 0.071 | 0.377 |
| **DG(36:1)** | 0.186 | 0.021 | 0.129 | 0.188 | 5.455 | 0.112 | 0.152 | 0.911 | 0.971 |
| **DG(36:3)** | 0.274 | -0.008 | 0.126 | 0.185 | 5.403 | -0.041 | 0.033 | 0.967 | 0.988 |
| **DG(36:4e)** | 0.214 | -0.037 | 0.130 | 0.191 | 5.343 | -0.192 | 0.105 | 0.848 | 0.956 |
| **DG(36:5e)** | 0.233 | -0.019 | 0.127 | 0.185 | 5.548 | -0.100 | 0.070 | 0.921 | 0.974 |
| **DG(40:6)** | 0.281 | -0.341 | 0.153 | 0.224 | -2.547 | -1.524 | 0.070 | 0.132 | 0.483 |
| **PC(14:1e_18:1)** | 0.112 | 0.175 | 0.149 | 0.219 | 2.203 | 0.800 | 0.457 | 0.426 | 0.837 |
| **PC(14:1e_20:4)** | -0.013 | 0.171 | 0.153 | 0.224 | 2.166 | 0.764 | 0.930 | 0.447 | 0.844 |
| **PC(15:0_18:2)** | -0.118 | -0.017 | 0.143 | 0.210 | -3.886 | -0.080 | 0.411 | 0.937 | 0.974 |
| **PC(15:0_20:2)** | -0.105 | -0.057 | 0.143 | 0.209 | -3.793 | -0.273 | 0.466 | 0.786 | 0.949 |
| **PC(16:0_20:3)** | 0.005 | 0.285 | 0.148 | 0.217 | 2.227 | 1.312 | 0.972 | 0.193 | 0.597 |
| **PC(16:0_20:4)** | -0.107 | 0.495 | 0.144 | 0.211 | 2.370 | 2.345 | 0.459 | 0.022 | 0.306 |
| **PC(16:0_20:5)** | 0.001 | 0.535 | 0.144 | 0.210 | 2.123 | 2.544 | 0.994 | 0.013 | 0.275 |
| **PC(16:0_22:6)** | 0.150 | 0.419 | 0.123 | 0.180 | 4.280 | 2.331 | 0.226 | 0.022 | 0.306 |
| **PC(16:2e_16:0)** | 0.089 | -0.428 | 0.145 | 0.212 | -3.169 | -2.018 | 0.543 | 0.047 | 0.361 |
| **PC(18:0_20:3)** | 0.131 | 0.433 | 0.135 | 0.198 | 2.272 | 2.187 | 0.334 | 0.032 | 0.335 |
| **PC(18:0_20:4)** | -0.034 | 0.282 | 0.147 | 0.215 | 2.700 | 1.308 | 0.817 | 0.195 | 0.597 |
| **PC(18:0_22:6)** | 0.138 | 0.399 | 0.138 | 0.202 | 2.568 | 1.969 | 0.323 | 0.053 | 0.363 |
| **PC(18:1_13:0)** | -0.104 | -0.017 | 0.148 | 0.218 | -3.136 | -0.076 | 0.488 | 0.940 | 0.975 |
| **PC(18:1_20:3)** | 0.025 | 0.441 | 0.143 | 0.209 | 2.727 | 2.108 | 0.863 | 0.038 | 0.335 |
| **PC(18:1_20:4)** | 0.096 | 0.329 | 0.139 | 0.203 | 2.494 | 1.618 | 0.489 | 0.110 | 0.439 |
| **PC(18:2_18:2)** | -0.005 | 0.415 | 0.138 | 0.202 | 3.283 | 2.053 | 0.969 | 0.044 | 0.352 |
| **PC(18:2_20:4)** | -0.150 | 0.244 | 0.151 | 0.221 | -2.738 | 1.107 | 0.322 | 0.272 | 0.694 |
| **PC(20:0_18:1)** | 0.021 | -0.051 | 0.148 | 0.217 | -3.771 | -0.235 | 0.889 | 0.815 | 0.949 |
| **PC(20:2_18:2)** | -0.206 | -0.022 | 0.130 | 0.191 | -5.467 | -0.117 | 0.118 | 0.907 | 0.971 |
| **PC(22:0_11:3)** | -0.052 | -0.303 | 0.151 | 0.222 | -2.223 | -1.368 | 0.730 | 0.176 | 0.567 |
| **PC(28:0)** | -0.059 | -0.171 | 0.153 | 0.223 | -2.512 | -0.765 | 0.701 | 0.447 | 0.844 |
| **PC(30:2)** | 0.251 | 0.089 | 0.143 | 0.209 | 2.228 | 0.423 | 0.084 | 0.673 | 0.921 |
| **PC(31:0)** | -0.058 | 0.019 | 0.150 | 0.219 | -3.346 | 0.085 | 0.699 | 0.933 | 0.974 |
| **PC(32:0)** | -0.097 | -0.024 | 0.151 | 0.221 | -2.986 | -0.109 | 0.522 | 0.913 | 0.971 |
| **PC(32:1)** | -0.076 | -0.197 | 0.139 | 0.204 | -4.430 | -0.965 | 0.587 | 0.338 | 0.757 |
| **PC(32:2)** | -0.078 | -0.140 | 0.151 | 0.221 | -2.780 | -0.630 | 0.610 | 0.530 | 0.874 |
| **PC(33:1)** | -0.193 | 0.046 | 0.144 | 0.212 | -3.686 | 0.220 | 0.186 | 0.827 | 0.951 |
| **PC(33:2)** | 0.089 | -0.392 | 0.146 | 0.213 | -3.159 | -1.840 | 0.542 | 0.070 | 0.377 |
| **PC(34:0)** | 0.057 | -0.510 | 0.142 | 0.208 | -3.182 | -2.447 | 0.688 | 0.017 | 0.279 |
| **PC(34:1)** | -0.246 | -0.044 | 0.129 | 0.189 | -5.484 | -0.231 | 0.061 | 0.818 | 0.949 |
| **PC(34:2)** | -0.158 | -0.033 | 0.127 | 0.186 | -6.094 | -0.178 | 0.217 | 0.859 | 0.956 |
| **PC(34:3)** | -0.267 | 0.051 | 0.149 | 0.219 | -2.088 | 0.233 | 0.078 | 0.816 | 0.949 |
| **PC(34:5)** | -0.077 | -0.132 | 0.150 | 0.220 | -2.770 | -0.598 | 0.610 | 0.552 | 0.874 |
| **PC(35:1)** | -0.193 | 0.100 | 0.139 | 0.203 | -4.281 | 0.493 | 0.169 | 0.624 | 0.906 |
| **PC(35:3)** | 0.031 | -0.133 | 0.143 | 0.209 | -4.310 | -0.634 | 0.827 | 0.528 | 0.874 |
| **PC(35:4)** | -0.219 | 0.074 | 0.133 | 0.194 | -5.344 | 0.383 | 0.103 | 0.703 | 0.927 |
| **PC(36:0)** | 0.049 | -0.166 | 0.139 | 0.204 | -4.816 | -0.812 | 0.724 | 0.419 | 0.835 |
| **PC(36:1)** | -0.195 | -0.085 | 0.133 | 0.194 | -5.110 | -0.435 | 0.145 | 0.665 | 0.921 |
| **PC(36:2)** | -0.112 | 0.107 | 0.137 | 0.200 | 4.574 | 0.534 | 0.416 | 0.595 | 0.899 |
| **PC(36:3)** | 0.079 | 0.148 | 0.144 | 0.211 | 2.319 | 0.702 | 0.583 | 0.485 | 0.855 |
| **PC(36:4)** | -0.126 | -0.061 | 0.142 | 0.208 | -4.151 | -0.293 | 0.379 | 0.770 | 0.949 |
| **PC(36:6)** | -0.089 | 0.080 | 0.146 | 0.214 | -3.968 | 0.373 | 0.546 | 0.711 | 0.927 |
| **PC(37:2)** | 0.285 | -0.488 | 0.147 | 0.215 | -2.954 | -2.267 | 0.056 | 0.026 | 0.308 |
| **PC(37:3)** | 0.218 | 0.304 | 0.142 | 0.207 | 2.001 | 1.468 | 0.128 | 0.146 | 0.513 |
| **PC(37:4)** | 0.013 | -0.089 | 0.152 | 0.222 | -2.884 | -0.403 | 0.933 | 0.688 | 0.927 |
| **PC(38:2)** | 0.087 | -0.139 | 0.152 | 0.223 | -2.922 | -0.622 | 0.570 | 0.536 | 0.874 |
| **PC(38:4)** | -0.245 | 0.153 | 0.148 | 0.217 | -3.188 | 0.708 | 0.102 | 0.481 | 0.855 |
| **PC(38:7)** | -0.133 | -0.112 | 0.149 | 0.218 | -2.612 | -0.515 | 0.373 | 0.608 | 0.902 |
| **PC(38:8)** | -0.099 | 0.038 | 0.147 | 0.216 | -3.553 | 0.175 | 0.501 | 0.861 | 0.956 |
| **PC(40:4)** | -0.082 | 0.053 | 0.147 | 0.215 | -3.265 | 0.248 | 0.579 | 0.805 | 0.949 |
| **PC(40:5)** | -0.023 | 0.173 | 0.147 | 0.215 | -3.257 | 0.804 | 0.877 | 0.424 | 0.837 |
| **PC(40:6)** | 0.099 | -0.171 | 0.139 | 0.203 | -5.067 | -0.839 | 0.480 | 0.404 | 0.825 |
| **PC(42:7)** | 0.022 | -0.257 | 0.146 | 0.214 | -3.135 | -1.196 | 0.882 | 0.235 | 0.651 |
| **PC(42:9)** | 0.121 | -0.265 | 0.151 | 0.221 | -3.080 | -1.200 | 0.425 | 0.234 | 0.651 |
| **PC(44:10)** | -0.102 | -0.094 | 0.151 | 0.222 | -2.560 | -0.426 | 0.504 | 0.671 | 0.921 |
| **PC(44:5)** | 0.031 | -0.240 | 0.145 | 0.213 | -3.751 | -1.127 | 0.834 | 0.263 | 0.694 |
| **PE(16:0_18:1)** | 0.028 | 0.180 | 0.149 | 0.218 | 2.300 | 0.825 | 0.852 | 0.412 | 0.832 |
| **PE(16:0_20:3)** | -0.059 | 0.445 | 0.144 | 0.210 | 2.399 | 2.117 | 0.681 | 0.038 | 0.335 |
| **PE(16:0_20:4)** | -0.218 | 0.164 | 0.151 | 0.221 | -2.618 | 0.742 | 0.154 | 0.461 | 0.848 |
| **PE(16:0p_18:1)** | -0.193 | 0.082 | 0.152 | 0.223 | -2.312 | 0.367 | 0.209 | 0.715 | 0.927 |
| **PE(16:0p_20:4)** | -0.054 | 0.001 | 0.148 | 0.217 | -3.568 | 0.006 | 0.719 | 0.995 | 0.996 |
| **PE(16:0p_22:6)** | -0.152 | 0.033 | 0.147 | 0.216 | -3.542 | 0.152 | 0.306 | 0.879 | 0.962 |
| **PE(18:0_18:1)** | -0.016 | 0.395 | 0.142 | 0.207 | 2.699 | 1.903 | 0.912 | 0.061 | 0.374 |
| **PE(18:0p_18:1)** | -0.203 | -0.050 | 0.150 | 0.220 | -2.486 | -0.225 | 0.180 | 0.823 | 0.950 |
| **PE(18:0p_18:2)** | -0.115 | 0.078 | 0.153 | 0.224 | -2.660 | 0.347 | 0.456 | 0.730 | 0.932 |
| **PE(18:0p_20:4)** | -0.112 | 0.087 | 0.156 | 0.228 | -2.264 | 0.382 | 0.475 | 0.703 | 0.927 |
| **PE(18:0p_22:4)** | -0.193 | 0.149 | 0.148 | 0.217 | -3.253 | 0.689 | 0.197 | 0.493 | 0.859 |
| **PE(18:1e)** | -0.077 | 0.371 | 0.144 | 0.211 | 3.183 | 1.760 | 0.594 | 0.083 | 0.408 |
| **PE(18:1p_18:1)** | 0.032 | 0.220 | 0.144 | 0.211 | 2.813 | 1.043 | 0.823 | 0.300 | 0.728 |
| **PE(20:0p_22:6)** | -0.255 | 0.249 | 0.151 | 0.221 | -2.824 | 1.131 | 0.095 | 0.262 | 0.694 |
| **PI(16:0_18:1)** | -0.120 | -0.194 | 0.149 | 0.219 | -2.597 | -0.887 | 0.423 | 0.378 | 0.794 |
| **PI(16:0_20:3)** | 0.160 | 0.055 | 0.138 | 0.202 | 3.820 | 0.274 | 0.248 | 0.785 | 0.949 |
| **PI(18:0_18:1)** | -0.166 | -0.168 | 0.140 | 0.206 | -3.768 | -0.817 | 0.240 | 0.417 | 0.835 |
| **PI(18:0_18:2)** | 0.026 | -0.421 | 0.123 | 0.180 | -6.395 | -2.346 | 0.836 | 0.022 | 0.306 |
| **PI(18:0_18:3)** | 0.011 | 0.120 | 0.150 | 0.219 | 2.645 | 0.549 | 0.942 | 0.585 | 0.890 |
| **PI(18:0_20:4)** | -0.034 | 0.208 | 0.142 | 0.208 | 4.282 | 0.998 | 0.813 | 0.322 | 0.754 |
| **PI(18:1_18:2)** | 0.083 | 0.025 | 0.146 | 0.214 | 2.483 | 0.116 | 0.572 | 0.908 | 0.971 |
| **PI(18:1_20:4)** | 0.038 | 0.171 | 0.140 | 0.205 | 3.910 | 0.834 | 0.790 | 0.407 | 0.826 |
| **PI(34:1)** | 0.027 | 0.408 | 0.148 | 0.216 | 2.214 | 1.886 | 0.856 | 0.063 | 0.377 |
| **PI(36:3)** | -0.163 | 0.603 | 0.148 | 0.217 | 1.893 | 2.784 | 0.273 | 0.007 | 0.275 |
| **SM(d16:1_20:0)** | -0.051 | -0.313 | 0.128 | 0.187 | -5.851 | -1.673 | 0.693 | 0.098 | 0.431 |
| **SM(d16:1_24:3)** | -0.036 | -0.367 | 0.130 | 0.190 | -5.339 | -1.930 | 0.783 | 0.057 | 0.367 |
| **SM(d17:1_13:0)** | 0.353 | -0.668 | 0.142 | 0.209 | -3.083 | -3.200 | 0.016 | 0.002 | 0.275 |
| **SM(d17:1_18:3)** | 0.041 | -0.405 | 0.134 | 0.196 | -5.026 | -2.064 | 0.763 | 0.043 | 0.350 |
| **SM(d18:0_16:1)** | -0.011 | -0.371 | 0.128 | 0.188 | -5.692 | -1.978 | 0.935 | 0.052 | 0.361 |
| **SM(d18:1_18:3)** | -0.041 | -0.382 | 0.135 | 0.198 | -4.436 | -1.929 | 0.761 | 0.058 | 0.367 |
| **SM(d18:1_21:0)** | 0.248 | -0.152 | 0.139 | 0.204 | 4.317 | -0.743 | 0.079 | 0.460 | 0.848 |
| **SM(d18:1_24:0)** | 0.178 | -0.198 | 0.148 | 0.216 | 3.303 | -0.915 | 0.233 | 0.363 | 0.791 |
| **SM(d18:1_24:3)** | -0.029 | -0.351 | 0.129 | 0.189 | -5.501 | -1.854 | 0.823 | 0.068 | 0.377 |
| **SM(d18:2_16:0)** | 0.147 | -0.028 | 0.136 | 0.199 | 5.004 | -0.140 | 0.282 | 0.889 | 0.969 |
| **SM(d18:2_18:3)** | 0.041 | -0.335 | 0.138 | 0.202 | -4.677 | -1.656 | 0.768 | 0.102 | 0.431 |
| **SM(d18:2_24:3)** | 0.240 | -0.470 | 0.145 | 0.213 | -3.585 | -2.207 | 0.103 | 0.030 | 0.327 |
| **SM(d28:1)** | -0.011 | -0.365 | 0.141 | 0.207 | -3.856 | -1.762 | 0.940 | 0.082 | 0.408 |
| **SM(d30:1)** | 0.219 | -0.230 | 0.142 | 0.208 | 4.017 | -1.105 | 0.127 | 0.273 | 0.694 |
| **SM(d31:1)** | 0.097 | -0.125 | 0.139 | 0.204 | 4.464 | -0.614 | 0.489 | 0.541 | 0.874 |
| **SM(d32:0)** | 0.052 | -0.358 | 0.141 | 0.206 | -4.269 | -1.736 | 0.714 | 0.087 | 0.408 |
| **SM(d32:4)** | 0.166 | -0.585 | 0.134 | 0.197 | -4.366 | -2.971 | 0.222 | 0.004 | 0.275 |
| **SM(d33:0)** | 0.110 | 0.150 | 0.145 | 0.213 | 2.741 | 0.705 | 0.452 | 0.483 | 0.855 |
| **SM(d33:1)** | 0.095 | -0.521 | 0.139 | 0.204 | -3.923 | -2.551 | 0.497 | 0.013 | 0.275 |
| **SM(d34:0)** | 0.016 | -0.327 | 0.138 | 0.202 | -4.347 | -1.614 | 0.906 | 0.111 | 0.439 |
| **SM(d34:1)** | 0.170 | -0.012 | 0.144 | 0.211 | 3.815 | -0.055 | 0.241 | 0.956 | 0.987 |
| **SM(d34:2)** | 0.132 | -0.416 | 0.141 | 0.206 | -4.237 | -2.014 | 0.354 | 0.048 | 0.361 |
| **SM(d34:4)** | -0.111 | -0.261 | 0.140 | 0.205 | -3.918 | -1.270 | 0.429 | 0.208 | 0.617 |
| **SM(d34:5)** | 0.067 | -0.400 | 0.149 | 0.218 | -2.674 | -1.837 | 0.654 | 0.070 | 0.377 |
| **SM(d35:1)** | 0.204 | -0.462 | 0.148 | 0.216 | -2.982 | -2.134 | 0.171 | 0.036 | 0.335 |
| **SM(d35:2)** | 0.002 | -0.333 | 0.139 | 0.203 | -4.396 | -1.635 | 0.988 | 0.106 | 0.437 |
| **SM(d35:4)** | 0.085 | -0.104 | 0.145 | 0.212 | 3.468 | -0.488 | 0.558 | 0.627 | 0.908 |
| **SM(d36:0)** | 0.195 | -0.229 | 0.152 | 0.223 | -2.414 | -1.028 | 0.203 | 0.307 | 0.729 |
| **SM(d36:1)** | 0.023 | -0.231 | 0.149 | 0.219 | -2.942 | -1.057 | 0.876 | 0.294 | 0.722 |
| **SM(d36:2)** | -0.081 | -0.344 | 0.133 | 0.194 | -4.800 | -1.773 | 0.545 | 0.080 | 0.407 |
| **SM(d36:3)** | 0.365 | -0.568 | 0.146 | 0.213 | -3.162 | -2.662 | 0.014 | 0.010 | 0.275 |
| **SM(d36:5)** | 0.155 | -0.033 | 0.134 | 0.196 | 5.167 | -0.170 | 0.251 | 0.865 | 0.956 |
| **SM(d37:1)** | 0.043 | -0.301 | 0.145 | 0.212 | -3.650 | -1.416 | 0.769 | 0.161 | 0.537 |
| **SM(d37:2)** | -0.012 | -0.429 | 0.136 | 0.199 | -4.339 | -2.161 | 0.929 | 0.034 | 0.335 |
| **SM(d38:1)** | 0.006 | -0.419 | 0.127 | 0.186 | -5.714 | -2.250 | 0.961 | 0.027 | 0.308 |
| **SM(d38:2)** | 0.027 | -0.458 | 0.137 | 0.200 | -4.254 | -2.285 | 0.844 | 0.025 | 0.308 |
| **SM(d38:3)** | -0.097 | -0.231 | 0.146 | 0.214 | -3.101 | -1.081 | 0.507 | 0.283 | 0.708 |
| **SM(d39:1)** | -0.031 | -0.413 | 0.131 | 0.192 | -5.037 | -2.153 | 0.811 | 0.035 | 0.335 |
| **SM(d39:2)** | 0.108 | -0.516 | 0.139 | 0.203 | -4.075 | -2.539 | 0.440 | 0.013 | 0.275 |
| **SM(d40:1)** | 0.038 | -0.453 | 0.135 | 0.198 | -4.600 | -2.294 | 0.778 | 0.025 | 0.308 |
| **SM(d40:2)** | 0.084 | -0.319 | 0.148 | 0.217 | -3.198 | -1.468 | 0.572 | 0.146 | 0.513 |
| **SM(d40:3)** | 0.118 | -0.458 | 0.149 | 0.218 | -2.516 | -2.098 | 0.433 | 0.039 | 0.335 |
| **SM(d40:4)** | 0.091 | -0.048 | 0.134 | 0.197 | 5.489 | -0.243 | 0.500 | 0.809 | 0.949 |
| **SM(d41:0)** | -0.007 | -0.278 | 0.142 | 0.209 | -4.042 | -1.331 | 0.960 | 0.187 | 0.591 |
| **SM(d41:1)** | -0.021 | -0.351 | 0.131 | 0.192 | -5.369 | -1.829 | 0.876 | 0.071 | 0.377 |
| **SM(d41:3)** | -0.070 | -0.245 | 0.141 | 0.206 | -4.097 | -1.189 | 0.622 | 0.238 | 0.654 |
| **SM(d41:4)** | -0.070 | -0.397 | 0.135 | 0.198 | -3.956 | -2.003 | 0.605 | 0.049 | 0.361 |
| **SM(d42:1)** | 0.103 | -0.435 | 0.138 | 0.202 | -4.402 | -2.152 | 0.458 | 0.035 | 0.335 |
| **SM(d42:2)** | 0.028 | -0.507 | 0.129 | 0.189 | -4.835 | -2.688 | 0.826 | 0.009 | 0.275 |
| **SM(d42:3)** | 0.041 | -0.494 | 0.140 | 0.205 | -3.677 | -2.409 | 0.768 | 0.018 | 0.288 |
| **SM(d42:4)** | 0.052 | -0.142 | 0.140 | 0.205 | 4.700 | -0.691 | 0.710 | 0.491 | 0.859 |
| **SM(d43:1)** | 0.106 | -0.500 | 0.147 | 0.215 | -2.652 | -2.328 | 0.473 | 0.023 | 0.306 |
| **SM(d43:2)** | 0.167 | -0.606 | 0.140 | 0.204 | -3.544 | -2.966 | 0.234 | 0.004 | 0.275 |
| **SM(d43:3)** | -0.023 | -0.339 | 0.138 | 0.203 | -4.225 | -1.669 | 0.871 | 0.099 | 0.431 |
| **SM(d43:4)** | 0.018 | -0.343 | 0.139 | 0.203 | -4.466 | -1.690 | 0.894 | 0.095 | 0.426 |
| **SM(d44:2)** | 0.109 | 0.019 | 0.145 | 0.212 | 2.591 | 0.090 | 0.452 | 0.929 | 0.974 |
| **SM(d44:3)** | 0.171 | -0.063 | 0.141 | 0.207 | 3.902 | -0.303 | 0.232 | 0.763 | 0.948 |
| **SM(d44:4)** | 0.019 | -0.421 | 0.136 | 0.200 | -4.132 | -2.107 | 0.887 | 0.038 | 0.335 |
| **SM(d44:5)** | 0.134 | -0.575 | 0.131 | 0.192 | -5.020 | -3.001 | 0.308 | 0.004 | 0.275 |
| **SM(d44:6)** | 0.054 | -0.427 | 0.139 | 0.204 | -4.231 | -2.092 | 0.699 | 0.040 | 0.335 |
| **SM(t18:0_16:1)** | 0.193 | -0.243 | 0.147 | 0.215 | -3.827 | -1.129 | 0.193 | 0.263 | 0.694 |
| **SM(t18:0_24:2)** | 0.163 | -0.509 | 0.143 | 0.209 | -3.595 | -2.436 | 0.256 | 0.017 | 0.279 |
| **SM(t32:1)** | 0.037 | -0.385 | 0.150 | 0.219 | -2.577 | -1.754 | 0.808 | 0.084 | 0.408 |
| **SM(t34:0)** | 0.023 | -0.388 | 0.143 | 0.210 | -3.580 | -1.848 | 0.870 | 0.069 | 0.377 |
| **SM(t34:1)** | 0.083 | -0.378 | 0.141 | 0.207 | -4.247 | -1.825 | 0.560 | 0.072 | 0.377 |
| **SM(t34:2)** | 0.149 | -0.336 | 0.143 | 0.209 | -4.119 | -1.608 | 0.299 | 0.112 | 0.440 |
| **SM(t36:1)** | -0.032 | -0.083 | 0.151 | 0.222 | -3.017 | -0.376 | 0.834 | 0.708 | 0.927 |
| **SM(t36:2)** | 0.051 | 0.188 | 0.143 | 0.209 | 3.147 | 0.900 | 0.720 | 0.371 | 0.791 |
| **SM(t38:3)** | 0.165 | -0.298 | 0.142 | 0.208 | 4.082 | -1.429 | 0.250 | 0.157 | 0.532 |
| **SM(t39:6)** | -0.061 | 0.211 | 0.149 | 0.218 | 2.644 | 0.965 | 0.682 | 0.337 | 0.757 |
| **SM(t42:1)** | 0.240 | -0.578 | 0.139 | 0.204 | -4.112 | -2.832 | 0.089 | 0.006 | 0.275 |
| **TG(11:0_9:0_9:0)** | 0.176 | -0.041 | 0.151 | 0.221 | 2.012 | -0.185 | 0.248 | 0.854 | 0.956 |
| **TG(12:0_12:0_14:0)** | -0.095 | -0.113 | 0.154 | 0.225 | -2.323 | -0.503 | 0.539 | 0.616 | 0.903 |
| **TG(12:0_17:1_18:2)** | -0.122 | 0.473 | 0.144 | 0.211 | 2.701 | 2.243 | 0.399 | 0.028 | 0.308 |
| **TG(12:0_18:2_18:2)** | -0.159 | 0.555 | 0.134 | 0.196 | 3.383 | 2.832 | 0.237 | 0.006 | 0.275 |
| **TG(14:0_14:3_18:2)** | -0.136 | 0.470 | 0.143 | 0.209 | 2.924 | 2.250 | 0.345 | 0.027 | 0.308 |
| **TG(14:0_18:2_20:5)** | 0.000 | 0.258 | 0.134 | 0.196 | 4.854 | 1.312 | 1.000 | 0.194 | 0.597 |
| **TG(14:0_18:3_18:3)** | -0.046 | 0.518 | 0.135 | 0.197 | 3.800 | 2.623 | 0.731 | 0.011 | 0.275 |
| **TG(14:0e_18:0_20:1)** | 0.057 | 0.162 | 0.149 | 0.219 | 2.411 | 0.740 | 0.705 | 0.462 | 0.848 |
| **TG(15:0_14:1_16:1)** | -0.048 | 0.463 | 0.129 | 0.189 | 3.770 | 2.453 | 0.709 | 0.017 | 0.279 |
| **TG(15:0_15:0_15:0)** | -0.193 | 0.055 | 0.154 | 0.225 | -2.196 | 0.243 | 0.214 | 0.809 | 0.949 |
| **TG(15:0_16:0_16:0)** | -0.239 | 0.028 | 0.152 | 0.222 | -1.933 | 0.125 | 0.119 | 0.901 | 0.971 |
| **TG(15:0_16:0_16:1)** | -0.263 | 0.022 | 0.149 | 0.218 | -2.112 | 0.098 | 0.082 | 0.922 | 0.974 |
| **TG(15:0_16:0_18:1)** | -0.249 | 0.038 | 0.147 | 0.216 | -2.934 | 0.175 | 0.095 | 0.861 | 0.956 |
| **TG(15:0_16:0_18:3)** | -0.193 | 0.211 | 0.150 | 0.220 | 2.791 | 0.959 | 0.203 | 0.341 | 0.757 |
| **TG(15:0_16:0_20:5)** | -0.063 | 0.480 | 0.133 | 0.195 | 3.896 | 2.464 | 0.637 | 0.016 | 0.279 |
| **TG(15:0_16:1_18:2)** | -0.234 | 0.087 | 0.147 | 0.215 | -3.064 | 0.407 | 0.115 | 0.685 | 0.927 |
| **TG(15:0_16:1_20:5)** | 0.059 | 0.223 | 0.137 | 0.200 | 2.947 | 1.115 | 0.670 | 0.268 | 0.694 |
| **TG(15:0_18:1_20:4)** | -0.173 | -0.132 | 0.124 | 0.181 | -6.344 | -0.728 | 0.166 | 0.469 | 0.848 |
| **TG(15:0_18:1_22:6)** | 0.129 | -0.203 | 0.143 | 0.210 | -4.230 | -0.968 | 0.372 | 0.336 | 0.757 |
| **TG(15:0_18:2_18:2)** | -0.008 | 0.146 | 0.141 | 0.206 | 3.370 | 0.708 | 0.956 | 0.481 | 0.855 |
| **TG(15:0_18:2_20:5)** | -0.095 | -0.078 | 0.148 | 0.216 | -3.275 | -0.362 | 0.522 | 0.719 | 0.927 |
| **TG(16:0_11:1_18:1)** | 0.025 | -0.089 | 0.149 | 0.218 | -3.349 | -0.409 | 0.865 | 0.684 | 0.927 |
| **TG(16:0_12:0_17:1)** | -0.178 | 0.049 | 0.142 | 0.208 | -4.099 | 0.234 | 0.213 | 0.816 | 0.949 |
| **TG(16:0_12:1_18:1)** | -0.008 | 0.308 | 0.139 | 0.204 | 2.959 | 1.506 | 0.957 | 0.136 | 0.490 |
| **TG(16:0_13:0_16:1)** | -0.092 | 0.008 | 0.148 | 0.216 | -3.641 | 0.035 | 0.536 | 0.972 | 0.989 |
| **TG(16:0_14:0_14:0)** | -0.185 | 0.128 | 0.151 | 0.221 | -2.794 | 0.581 | 0.224 | 0.563 | 0.886 |
| **TG(16:0_14:0_16:0)** | -0.195 | -0.018 | 0.144 | 0.212 | -3.607 | -0.085 | 0.180 | 0.932 | 0.974 |
| **TG(16:0_14:0_18:1)** | -0.125 | -0.164 | 0.138 | 0.202 | -4.519 | -0.815 | 0.366 | 0.418 | 0.835 |
| **TG(16:0_14:0_18:3)** | -0.142 | 0.468 | 0.138 | 0.202 | 3.966 | 2.317 | 0.305 | 0.023 | 0.306 |
| **TG(16:0_14:1_16:1)** | -0.198 | 0.579 | 0.146 | 0.213 | 2.444 | 2.711 | 0.178 | 0.008 | 0.275 |
| **TG(16:0_14:1_18:2)** | 0.066 | 0.142 | 0.147 | 0.215 | 2.724 | 0.662 | 0.656 | 0.510 | 0.874 |
| **TG(16:0_16:0_16:0)** | -0.188 | 0.057 | 0.146 | 0.215 | -3.588 | 0.264 | 0.204 | 0.793 | 0.949 |
| **TG(16:0_16:0_17:0)** | -0.220 | 0.164 | 0.151 | 0.222 | -2.546 | 0.738 | 0.151 | 0.463 | 0.848 |
| **TG(16:0_16:0_18:1)** | -0.168 | 0.045 | 0.146 | 0.214 | -3.662 | 0.209 | 0.254 | 0.835 | 0.953 |
| **TG(16:0_16:0_18:2)** | -0.044 | 0.086 | 0.151 | 0.221 | 2.067 | 0.392 | 0.772 | 0.696 | 0.927 |
| **TG(16:0_16:0_18:3)** | -0.191 | -0.103 | 0.139 | 0.203 | -3.892 | -0.505 | 0.172 | 0.615 | 0.903 |
| **TG(16:0_16:0_20:4)** | -0.081 | 0.256 | 0.137 | 0.200 | 4.670 | 1.278 | 0.555 | 0.205 | 0.616 |
| **TG(16:0_16:0_20:5)** | -0.098 | 0.417 | 0.143 | 0.209 | 3.048 | 1.990 | 0.494 | 0.050 | 0.361 |
| **TG(16:0_16:0_23:0)** | -0.240 | 0.135 | 0.149 | 0.219 | -2.947 | 0.618 | 0.112 | 0.539 | 0.874 |
| **TG(16:0_16:0_24:0)** | -0.113 | 0.339 | 0.146 | 0.214 | 3.298 | 1.582 | 0.443 | 0.118 | 0.455 |
| **TG(16:0_16:0_24:1)** | 0.016 | 0.320 | 0.149 | 0.219 | 2.608 | 1.462 | 0.916 | 0.148 | 0.514 |
| **TG(16:0_17:0_18:1)** | -0.195 | -0.025 | 0.149 | 0.218 | -2.750 | -0.117 | 0.192 | 0.908 | 0.971 |
| **TG(16:0_17:0_20:4)** | -0.141 | -0.132 | 0.133 | 0.195 | -5.186 | -0.676 | 0.293 | 0.501 | 0.867 |
| **TG(16:0_17:1_18:1)** | -0.300 | 0.041 | 0.132 | 0.194 | -4.875 | 0.212 | 0.026 | 0.833 | 0.953 |
| **TG(16:0_17:1_18:3)** | -0.199 | 0.375 | 0.133 | 0.195 | 5.424 | 1.917 | 0.141 | 0.059 | 0.370 |
| **TG(16:0_17:1_20:5)** | 0.117 | 0.383 | 0.135 | 0.198 | 2.548 | 1.936 | 0.389 | 0.057 | 0.367 |
| **TG(16:0_18:1_18:2)** | -0.092 | -0.056 | 0.144 | 0.211 | -4.039 | -0.263 | 0.527 | 0.793 | 0.949 |
| **TG(16:0_18:1_18:3)** | -0.076 | -0.180 | 0.140 | 0.205 | -4.427 | -0.877 | 0.588 | 0.383 | 0.795 |
| **TG(16:0_18:1_19:0)** | 0.078 | 0.155 | 0.126 | 0.184 | 6.001 | 0.838 | 0.539 | 0.405 | 0.825 |
| **TG(16:0_18:1_20:3)** | 0.106 | 0.171 | 0.129 | 0.189 | 4.845 | 0.903 | 0.413 | 0.370 | 0.791 |
| **TG(16:0_18:1_22:1)** | 0.091 | 0.247 | 0.136 | 0.199 | 4.300 | 1.242 | 0.507 | 0.218 | 0.632 |
| **TG(16:0_18:1_22:6)** | 0.173 | -0.251 | 0.148 | 0.217 | -3.689 | -1.158 | 0.246 | 0.251 | 0.675 |
| **TG(16:0_18:1_24:0)** | -0.053 | 0.351 | 0.151 | 0.222 | 2.338 | 1.581 | 0.728 | 0.118 | 0.455 |
| **TG(16:0_18:1_24:1)** | 0.058 | 0.257 | 0.146 | 0.214 | 2.780 | 1.199 | 0.692 | 0.234 | 0.651 |
| **TG(16:0_18:2_18:3)** | 0.034 | 0.216 | 0.126 | 0.184 | 6.054 | 1.173 | 0.787 | 0.245 | 0.667 |
| **TG(16:0_18:3_18:3)** | -0.072 | 0.385 | 0.133 | 0.194 | 5.263 | 1.980 | 0.587 | 0.051 | 0.361 |
| **TG(16:0_18:3_20:5)** | 0.177 | 0.105 | 0.144 | 0.211 | 2.522 | 0.500 | 0.224 | 0.619 | 0.903 |
| **TG(16:0_20:4_22:6)** | 0.063 | -0.147 | 0.147 | 0.216 | -3.619 | -0.682 | 0.671 | 0.498 | 0.864 |
| **TG(16:0_22:6_24:0)** | 0.335 | 0.010 | 0.140 | 0.206 | 2.804 | 0.047 | 0.020 | 0.962 | 0.988 |
| **TG(16:0_6:0_11:1)** | 0.081 | 0.060 | 0.152 | 0.223 | 2.420 | 0.270 | 0.594 | 0.788 | 0.949 |
| **TG(16:0_8:0_14:0)** | -0.092 | 0.269 | 0.152 | 0.223 | 2.603 | 1.210 | 0.546 | 0.230 | 0.651 |
| **TG(16:0e_16:0_18:2)** | 0.104 | -0.139 | 0.153 | 0.224 | -3.043 | -0.621 | 0.497 | 0.537 | 0.874 |
| **TG(16:0e_18:0_20:1)** | 0.137 | 0.034 | 0.152 | 0.223 | 2.092 | 0.153 | 0.372 | 0.879 | 0.962 |
| **TG(16:1_12:0_18:1)** | 0.013 | 0.103 | 0.151 | 0.222 | 2.248 | 0.466 | 0.933 | 0.643 | 0.917 |
| **TG(16:1_12:0_18:2)** | -0.046 | 0.421 | 0.142 | 0.208 | 3.490 | 2.023 | 0.747 | 0.047 | 0.361 |
| **TG(16:1_14:0_18:3)** | -0.049 | 0.341 | 0.144 | 0.211 | 2.012 | 1.614 | 0.734 | 0.111 | 0.439 |
| **TG(16:1_16:1_18:3)** | 0.130 | -0.125 | 0.151 | 0.221 | -3.146 | -0.565 | 0.389 | 0.574 | 0.890 |
| **TG(16:1_17:1_18:1)** | -0.143 | -0.098 | 0.130 | 0.190 | -5.778 | -0.517 | 0.274 | 0.607 | 0.902 |
| **TG(16:1_17:1_18:2)** | 0.116 | 0.145 | 0.135 | 0.198 | 3.623 | 0.730 | 0.393 | 0.467 | 0.848 |
| **TG(16:1_18:1_18:2)** | -0.003 | 0.341 | 0.124 | 0.181 | 5.084 | 1.881 | 0.978 | 0.064 | 0.377 |
| **TG(16:1_18:1_20:4)** | -0.006 | -0.059 | 0.154 | 0.226 | -2.025 | -0.262 | 0.971 | 0.794 | 0.949 |
| **TG(16:1_18:2_18:3)** | 0.088 | 0.294 | 0.143 | 0.209 | 2.570 | 1.407 | 0.537 | 0.164 | 0.541 |
| **TG(16:1_20:1_20:1)** | 0.156 | -0.092 | 0.142 | 0.209 | 3.938 | -0.441 | 0.277 | 0.660 | 0.921 |
| **TG(16:1_20:1_22:4)** | 0.106 | 0.231 | 0.136 | 0.200 | 3.646 | 1.158 | 0.439 | 0.251 | 0.675 |
| **TG(16:1_20:5_20:5)** | 0.088 | 0.503 | 0.126 | 0.185 | 3.204 | 2.724 | 0.488 | 0.008 | 0.275 |
| **TG(16:1e_16:0_16:0)** | 0.032 | -0.188 | 0.145 | 0.212 | -3.813 | -0.888 | 0.825 | 0.378 | 0.794 |
| **TG(17:0_17:1_17:1)** | -0.103 | 0.285 | 0.147 | 0.215 | 2.442 | 1.326 | 0.484 | 0.189 | 0.592 |
| **TG(17:0_17:1_19:0)** | -0.118 | -0.095 | 0.141 | 0.206 | -4.358 | -0.460 | 0.402 | 0.647 | 0.917 |
| **TG(17:0_18:1_18:2)** | -0.104 | -0.175 | 0.122 | 0.179 | -6.765 | -0.977 | 0.399 | 0.332 | 0.757 |
| **TG(17:0_18:1_20:3)** | 0.187 | -0.001 | 0.118 | 0.172 | 6.455 | -0.006 | 0.115 | 0.996 | 0.996 |
| **TG(17:0_18:1_20:4)** | -0.129 | -0.101 | 0.138 | 0.202 | -4.603 | -0.499 | 0.352 | 0.619 | 0.903 |
| **TG(17:0_18:1_20:5)** | 0.176 | -0.005 | 0.129 | 0.189 | 5.529 | -0.025 | 0.176 | 0.981 | 0.994 |
| **TG(17:0_18:1_22:4)** | -0.071 | -0.149 | 0.146 | 0.214 | -3.246 | -0.699 | 0.629 | 0.487 | 0.855 |
| **TG(17:0_18:1_22:5)** | -0.021 | -0.183 | 0.130 | 0.190 | -6.026 | -0.960 | 0.870 | 0.340 | 0.757 |
| **TG(17:0_18:2_20:3)** | -0.008 | -0.228 | 0.140 | 0.205 | -4.504 | -1.113 | 0.955 | 0.270 | 0.694 |
| **TG(17:0_18:2_22:6)** | -0.029 | -0.020 | 0.155 | 0.227 | -2.543 | -0.090 | 0.851 | 0.928 | 0.974 |
| **TG(18:0_12:1_16:0)** | -0.150 | -0.056 | 0.149 | 0.218 | -2.838 | -0.258 | 0.317 | 0.797 | 0.949 |
| **TG(18:0_16:0_16:1)** | -0.278 | 0.085 | 0.129 | 0.189 | -5.567 | 0.452 | 0.035 | 0.653 | 0.917 |
| **TG(18:0_16:0_17:1)** | 0.070 | 0.040 | 0.150 | 0.219 | 2.983 | 0.181 | 0.641 | 0.857 | 0.956 |
| **TG(18:0_16:0_19:0)** | -0.014 | 0.213 | 0.142 | 0.209 | 3.673 | 1.021 | 0.920 | 0.310 | 0.733 |
| **TG(18:0_16:0_21:0)** | 0.084 | 0.027 | 0.145 | 0.212 | 3.481 | 0.128 | 0.565 | 0.899 | 0.971 |
| **TG(18:0_16:0_23:0)** | -0.080 | 0.367 | 0.134 | 0.196 | 4.818 | 1.868 | 0.551 | 0.066 | 0.377 |
| **TG(18:0_16:0_24:0)** | -0.040 | 0.394 | 0.138 | 0.202 | 4.190 | 1.949 | 0.775 | 0.055 | 0.367 |
| **TG(18:0_16:1_18:0)** | -0.014 | 0.211 | 0.136 | 0.200 | 4.872 | 1.058 | 0.921 | 0.294 | 0.722 |
| **TG(18:0_16:1_24:0)** | 0.027 | 0.346 | 0.142 | 0.208 | 3.480 | 1.663 | 0.851 | 0.101 | 0.431 |
| **TG(18:0_18:0_18:0)** | -0.060 | 0.315 | 0.141 | 0.207 | 3.890 | 1.521 | 0.674 | 0.132 | 0.483 |
| **TG(18:0_18:0_20:0)** | -0.014 | 0.305 | 0.141 | 0.206 | 3.867 | 1.484 | 0.920 | 0.142 | 0.506 |
| **TG(18:0_18:1_20:0)** | 0.064 | 0.207 | 0.143 | 0.209 | 3.699 | 0.988 | 0.658 | 0.326 | 0.757 |
| **TG(18:0_18:1_22:4)** | 0.113 | 0.113 | 0.141 | 0.207 | 3.727 | 0.545 | 0.428 | 0.587 | 0.891 |
| **TG(18:0_18:1_22:6)** | 0.132 | -0.244 | 0.139 | 0.204 | -4.914 | -1.196 | 0.344 | 0.235 | 0.651 |
| **TG(18:0_18:1_24:0)** | -0.031 | 0.294 | 0.154 | 0.226 | 2.048 | 1.299 | 0.844 | 0.198 | 0.599 |
| **TG(18:0_20:4_22:5)** | -0.024 | -0.026 | 0.155 | 0.227 | -2.298 | -0.114 | 0.875 | 0.910 | 0.971 |
| **TG(18:0e_16:0_18:3)** | -0.057 | -0.063 | 0.154 | 0.225 | -2.521 | -0.278 | 0.710 | 0.782 | 0.949 |
| **TG(18:1_11:1_18:1)** | -0.091 | 0.124 | 0.154 | 0.226 | -2.602 | 0.551 | 0.558 | 0.584 | 0.890 |
| **TG(18:1_12:0_14:0)** | -0.100 | -0.109 | 0.145 | 0.212 | -3.819 | -0.513 | 0.492 | 0.610 | 0.902 |
| **TG(18:1_12:0_20:4)** | -0.065 | 0.253 | 0.143 | 0.209 | 3.285 | 1.210 | 0.653 | 0.230 | 0.651 |
| **TG(18:1_14:0_18:2)** | 0.044 | 0.168 | 0.143 | 0.210 | 3.103 | 0.801 | 0.757 | 0.426 | 0.837 |
| **TG(18:1_17:1_18:1)** | -0.006 | -0.102 | 0.154 | 0.226 | -2.433 | -0.452 | 0.968 | 0.653 | 0.917 |
| **TG(18:1_17:1_18:2)** | 0.155 | 0.115 | 0.124 | 0.182 | 5.428 | 0.631 | 0.215 | 0.530 | 0.874 |
| **TG(18:1_17:1_18:3)** | -0.109 | 0.134 | 0.146 | 0.214 | 3.750 | 0.623 | 0.460 | 0.535 | 0.874 |
| **TG(18:1_17:1_20:4)** | -0.124 | -0.112 | 0.144 | 0.211 | -3.748 | -0.528 | 0.393 | 0.599 | 0.901 |
| **TG(18:1_18:1_21:0)** | -0.113 | 0.219 | 0.142 | 0.209 | 4.129 | 1.050 | 0.431 | 0.297 | 0.726 |
| **TG(18:1_18:1_21:1)** | 0.172 | -0.040 | 0.145 | 0.212 | 3.371 | -0.190 | 0.239 | 0.850 | 0.956 |
| **TG(18:1_18:1_22:0)** | 0.149 | 0.185 | 0.128 | 0.188 | 5.374 | 0.983 | 0.251 | 0.329 | 0.757 |
| **TG(18:1_18:1_22:1)** | 0.131 | 0.061 | 0.143 | 0.209 | 3.487 | 0.290 | 0.361 | 0.773 | 0.949 |
| **TG(18:1_18:1_22:4)** | -0.021 | -0.150 | 0.135 | 0.198 | -5.280 | -0.759 | 0.874 | 0.450 | 0.844 |
| **TG(18:1_18:1_22:5)** | 0.032 | 0.361 | 0.135 | 0.197 | 3.373 | 1.831 | 0.813 | 0.071 | 0.377 |
| **TG(18:1_18:1_22:6)** | 0.218 | -0.323 | 0.152 | 0.223 | -2.554 | -1.444 | 0.156 | 0.153 | 0.524 |
| **TG(18:1_18:1_24:0)** | 0.082 | 0.236 | 0.149 | 0.218 | 2.714 | 1.086 | 0.582 | 0.281 | 0.707 |
| **TG(18:1_18:1_24:1)** | 0.165 | 0.121 | 0.143 | 0.210 | 3.380 | 0.576 | 0.251 | 0.566 | 0.887 |
| **TG(18:1_18:2_18:2)** | 0.028 | 0.176 | 0.136 | 0.199 | 4.785 | 0.887 | 0.839 | 0.378 | 0.794 |
| **TG(18:1_18:2_20:3)** | -0.006 | 0.083 | 0.152 | 0.223 | -2.945 | 0.372 | 0.970 | 0.711 | 0.927 |
| **TG(18:1_18:2_22:0)** | 0.165 | 0.040 | 0.138 | 0.202 | 4.203 | 0.199 | 0.235 | 0.843 | 0.956 |
| **TG(18:1_18:2_22:5)** | 0.105 | 0.160 | 0.139 | 0.204 | 3.383 | 0.783 | 0.453 | 0.436 | 0.844 |
| **TG(18:1_18:2_23:0)** | 0.068 | 0.305 | 0.135 | 0.198 | 4.006 | 1.541 | 0.614 | 0.128 | 0.483 |
| **TG(18:1_18:2_23:1)** | 0.138 | 0.039 | 0.143 | 0.210 | 3.149 | 0.186 | 0.337 | 0.853 | 0.956 |
| **TG(18:1_18:2_24:0)** | 0.165 | 0.126 | 0.134 | 0.196 | 4.609 | 0.643 | 0.221 | 0.523 | 0.874 |
| **TG(18:1_18:2_24:1)** | 0.203 | 0.011 | 0.140 | 0.205 | 3.741 | 0.052 | 0.151 | 0.959 | 0.987 |
| **TG(18:1_18:3_20:4)** | -0.231 | 0.129 | 0.144 | 0.211 | -3.790 | 0.612 | 0.112 | 0.543 | 0.874 |
| **TG(18:1_18:3_20:5)** | 0.161 | 0.073 | 0.148 | 0.216 | 2.192 | 0.337 | 0.279 | 0.737 | 0.935 |
| **TG(18:1_20:2_22:5)** | -0.044 | 0.364 | 0.144 | 0.210 | 2.300 | 1.732 | 0.762 | 0.088 | 0.408 |
| **TG(18:1_20:3_20:3)** | -0.097 | 0.062 | 0.152 | 0.222 | -2.334 | 0.277 | 0.523 | 0.782 | 0.949 |
| **TG(18:1_20:4_22:5)** | 0.072 | 0.120 | 0.149 | 0.218 | -3.006 | 0.549 | 0.631 | 0.585 | 0.890 |
| **TG(18:1_22:5_22:6)** | 0.050 | -0.116 | 0.151 | 0.221 | -3.134 | -0.525 | 0.740 | 0.601 | 0.901 |
| **TG(18:1e_16:0_18:2)** | 0.121 | -0.068 | 0.149 | 0.218 | -3.538 | -0.313 | 0.417 | 0.755 | 0.944 |
| **TG(18:2_20:4_22:6)** | 0.187 | 0.003 | 0.146 | 0.213 | 2.614 | 0.016 | 0.202 | 0.987 | 0.994 |
| **TG(18:3_14:1_18:2)** | -0.045 | 0.412 | 0.141 | 0.206 | 2.335 | 1.999 | 0.751 | 0.049 | 0.361 |
| **TG(18:3_14:1_18:3)** | 0.187 | 0.207 | 0.136 | 0.199 | 2.832 | 1.038 | 0.173 | 0.303 | 0.728 |
| **TG(18:3_18:2_20:5)** | 0.112 | -0.179 | 0.153 | 0.225 | -2.423 | -0.796 | 0.466 | 0.429 | 0.837 |
| **TG(18:3_18:2_22:5)** | 0.269 | -0.032 | 0.132 | 0.194 | 3.793 | -0.164 | 0.045 | 0.870 | 0.959 |
| **TG(18:4_14:0_16:1)** | -0.155 | 0.616 | 0.143 | 0.209 | 2.825 | 2.944 | 0.283 | 0.004 | 0.275 |
| **TG(18:4_16:0_16:1)** | -0.118 | 0.404 | 0.145 | 0.213 | 2.871 | 1.899 | 0.420 | 0.062 | 0.374 |
| **TG(18:4_16:0_20:4)** | -0.081 | 0.525 | 0.141 | 0.207 | 2.409 | 2.533 | 0.570 | 0.013 | 0.275 |
| **TG(18:4_16:1_18:2)** | 0.065 | 0.127 | 0.135 | 0.198 | 4.719 | 0.641 | 0.632 | 0.523 | 0.874 |
| **TG(18:4_16:1_18:3)** | -0.019 | -0.303 | 0.136 | 0.200 | -3.902 | -1.518 | 0.887 | 0.133 | 0.483 |
| **TG(18:4_18:1_20:4)** | 0.220 | 0.119 | 0.132 | 0.193 | 3.800 | 0.615 | 0.099 | 0.541 | 0.874 |
| **TG(19:0_18:1_18:1)** | 0.185 | 0.066 | 0.124 | 0.182 | 5.955 | 0.363 | 0.140 | 0.718 | 0.927 |
| **TG(19:0_18:2_18:2)** | -0.069 | -0.262 | 0.144 | 0.211 | -3.359 | -1.243 | 0.634 | 0.218 | 0.632 |
| **TG(19:1_18:0_18:1)** | -0.006 | 0.341 | 0.141 | 0.207 | 3.133 | 1.645 | 0.969 | 0.104 | 0.434 |
| **TG(19:1_18:1_18:2)** | 0.059 | -0.371 | 0.140 | 0.206 | -4.115 | -1.804 | 0.674 | 0.075 | 0.386 |
| **TG(19:1_18:1_20:4)** | -0.098 | -0.125 | 0.149 | 0.218 | -3.121 | -0.572 | 0.511 | 0.569 | 0.888 |
| **TG(20:0_16:0_18:1)** | 0.009 | 0.274 | 0.135 | 0.197 | 4.546 | 1.386 | 0.945 | 0.170 | 0.553 |
| **TG(20:0_16:0_24:1)** | 0.052 | 0.297 | 0.141 | 0.206 | 3.715 | 1.442 | 0.711 | 0.153 | 0.524 |
| **TG(20:0_18:1_18:1)** | 0.149 | 0.056 | 0.132 | 0.194 | 5.239 | 0.287 | 0.265 | 0.775 | 0.949 |
| **TG(20:0_18:1_20:4)** | 0.109 | -0.085 | 0.150 | 0.219 | 2.315 | -0.386 | 0.467 | 0.701 | 0.927 |
| **TG(20:0_18:2_18:2)** | 0.151 | 0.090 | 0.132 | 0.193 | 5.230 | 0.466 | 0.256 | 0.642 | 0.917 |
| **TG(20:0_18:2_22:6)** | 0.192 | 0.129 | 0.145 | 0.212 | 2.401 | 0.609 | 0.189 | 0.545 | 0.874 |
| **TG(20:1_14:1_22:4)** | 0.052 | -0.170 | 0.152 | 0.223 | -2.755 | -0.765 | 0.734 | 0.447 | 0.844 |
| **TG(20:1_18:1_22:6)** | -0.051 | -0.104 | 0.147 | 0.215 | -3.423 | -0.485 | 0.729 | 0.629 | 0.908 |
| **TG(20:1_20:4_20:4)** | 0.304 | -0.531 | 0.144 | 0.210 | -3.286 | -2.524 | 0.038 | 0.014 | 0.275 |
| **TG(20:1_20:4_20:5)** | 0.142 | 0.189 | 0.143 | 0.209 | 2.448 | 0.905 | 0.324 | 0.369 | 0.791 |
| **TG(20:3_18:2_18:2)** | 0.007 | -0.131 | 0.147 | 0.215 | -3.278 | -0.609 | 0.961 | 0.545 | 0.874 |
| **TG(20:5_14:1_18:2)** | 0.070 | 0.201 | 0.142 | 0.209 | 2.969 | 0.964 | 0.623 | 0.338 | 0.757 |
| **TG(22:2_14:1_14:1)** | -0.109 | -0.119 | 0.146 | 0.214 | -3.178 | -0.557 | 0.459 | 0.580 | 0.890 |
| **TG(22:5_18:2_18:2)** | -0.078 | 0.454 | 0.147 | 0.215 | 2.051 | 2.110 | 0.598 | 0.038 | 0.335 |
| **TG(25:0_16:0_16:0)** | -0.160 | 0.094 | 0.149 | 0.219 | -3.186 | 0.428 | 0.286 | 0.670 | 0.921 |
| **TG(26:0_16:0_16:0)** | -0.071 | 0.279 | 0.150 | 0.220 | 2.895 | 1.268 | 0.639 | 0.209 | 0.617 |
| **TG(26:0_18:1_18:1)** | 0.041 | 0.190 | 0.152 | 0.222 | 2.739 | 0.857 | 0.789 | 0.394 | 0.812 |
| **TG(26:0_18:1_18:2)** | 0.115 | 0.209 | 0.138 | 0.202 | 4.039 | 1.033 | 0.408 | 0.305 | 0.728 |
| **TG(26:1_18:1_18:1)** | 0.122 | 0.185 | 0.144 | 0.211 | 3.162 | 0.876 | 0.401 | 0.384 | 0.795 |
| **TG(26:1_18:1_18:2)** | 0.209 | 0.036 | 0.142 | 0.209 | 3.338 | 0.173 | 0.147 | 0.863 | 0.956 |
| **TG(28:0_16:0_18:1)** | -0.075 | 0.349 | 0.152 | 0.222 | 2.362 | 1.571 | 0.623 | 0.120 | 0.460 |
| **TG(28:0_18:1_18:1)** | 0.013 | 0.361 | 0.144 | 0.211 | 3.144 | 1.714 | 0.928 | 0.091 | 0.409 |
| **TG(29:0_16:0_16:0)** | -0.014 | 0.164 | 0.147 | 0.215 | 2.680 | 0.759 | 0.925 | 0.450 | 0.844 |
| **TG(29:0_18:1_18:1)** | 0.162 | 0.025 | 0.144 | 0.210 | 2.348 | 0.117 | 0.262 | 0.907 | 0.971 |
| **TG(30:0_16:0_18:1)** | 0.020 | 0.218 | 0.153 | 0.224 | 2.293 | 0.973 | 0.895 | 0.334 | 0.757 |
| **TG(33:4e)** | -0.160 | 0.651 | 0.137 | 0.201 | 3.490 | 3.241 | 0.247 | 0.002 | 0.275 |
| **TG(51:3)** | 0.124 | 0.064 | 0.139 | 0.203 | 4.028 | 0.315 | 0.373 | 0.753 | 0.944 |
| **TG(53:4)** | 0.119 | 0.191 | 0.126 | 0.185 | 5.017 | 1.034 | 0.348 | 0.305 | 0.728 |
| **TG(55:5)** | -0.096 | -0.096 | 0.148 | 0.216 | -3.498 | -0.442 | 0.518 | 0.659 | 0.921 |
| **TG(56:7)** | 0.040 | 0.310 | 0.139 | 0.204 | 3.022 | 1.517 | 0.775 | 0.134 | 0.483 |
| **TG(6:0_11:1_18:3)** | -0.048 | 0.187 | 0.140 | 0.206 | 4.034 | 0.910 | 0.732 | 0.366 | 0.791 |
| **TG(6:0_11:2_18:3)** | -0.024 | 0.278 | 0.150 | 0.220 | 2.490 | 1.265 | 0.874 | 0.210 | 0.617 |
| **TG(60:4e)** | 0.063 | 0.157 | 0.152 | 0.222 | 2.208 | 0.707 | 0.679 | 0.482 | 0.855 |
| **TG(60:6)** | 0.006 | 0.129 | 0.146 | 0.213 | 2.928 | 0.604 | 0.969 | 0.548 | 0.874 |
| **TG(67:2)** | 0.143 | 0.129 | 0.136 | 0.199 | 3.139 | 0.647 | 0.296 | 0.519 | 0.874 |

Beta.PRS/SE.PRS/ t.PRS/ Pval.PRS: Beta coefficient/standard error/t statistics/p value of association between PRS scores and individual lipids

Beta.PRS_CC/SE.PRS_CC/t.PRS_CC/Pval.PRS_CC: Beta coefficient/standard error/t statistics/p value of differential association of PRS with lipids between AD and controls control

Cer: ceramides; SM: sphingomyelins; ChE: Cholesteryl esters; DG: diglycerides; TG: triglycerides; PC: phosphatidylcholines; LPC: lyso- phosphatidylcholines; PE: phosphatidylethanolamines; PI: phosphatidylinositols
