# Supplementary material for: Plasma lipidome is dysregulated in Alzheimer’s disease and is associated with disease risk genes
Source: Transl Psychiatry. 2021 Jun 7;11:344. doi: 10.1038/s41398-021-01362-2 (PMC8180517; doi:10.1038/s41398-021-01362-2)
Supplement: Supplementary file 5 — Supplementary table 5. Significantly differential association of the AD risk variants and the SNPs with lipids between AD and controls [file 41398_2021_1362_MOESM5_ESM.docx]

**Supplementary table 5. Significantly differential association of the AD risk variants and the SNPs with lipids between AD and controls**

| **SNPname** | **Gene** | **Lipid** | **Beta.SNP** | **Beta.SNP_CC** | **SE.SNP** | **SE.SNP_CC** | **tvalue.SNP** | **tvalue.SNP_CC** | **Pval.SNP** | **Pval.SNP_CC** | **Adjusted Pval.SNP_CC** |
| --- | --- | --- | --- | --- | --- | --- | --- | --- | --- | --- | --- |
| rs10498633 | SLC24A4 | TG(19:1_18:1_20:4) | 0.389 | -0.768 | 0.170 | 0.212 | 2.294 | -3.623 | 0.025 | 0.001 | 0.950 |
| rs17125944 | FERMT2 | PI(16:0_18:1) | 0.403 | -0.762 | 0.190 | 0.228 | 2.126 | -3.347 | 0.037 | 0.001 | 0.950 |
| rs744373 | BIN1 | TG(18:1_17:1_18:2) | 0.272 | -0.583 | 0.121 | 0.176 | 2.258 | -3.319 | 0.027 | 0.001 | 0.950 |
| rs3865444 | CD33 | TG(15:0_16:0_18:3) | -0.175 | 0.684 | 0.133 | 0.207 | -1.310 | 3.299 | 0.194 | 0.001 | 0.950 |
| rs6733839 | BIN1 | TG(18:1_17:1_18:2) | 0.239 | -0.583 | 0.116 | 0.177 | 2.059 | -3.283 | 0.043 | 0.002 | 0.950 |
| rs983392 | MS4A6A | PI(16:0_20:3) | -0.060 | -0.580 | 0.120 | 0.178 | -0.499 | -3.259 | 0.619 | 0.002 | 0.950 |
| rs983392 | MS4A6A | SM(d36:1) | -0.447 | 0.661 | 0.137 | 0.203 | -3.266 | 3.258 | 0.002 | 0.002 | 0.950 |
| rs35349669 | INPP5D | PI(36:3) | -0.271 | 0.690 | 0.153 | 0.213 | -1.776 | 3.233 | 0.080 | 0.002 | 0.950 |
| rs9331896 | CLU | TG(18:4_16:0_16:1) | 0.301 | -0.661 | 0.136 | 0.206 | 2.220 | -3.215 | 0.029 | 0.002 | 0.950 |
| rs3752246 | ABCA7 | PI(18:1_18:2) | 0.200 | -0.634 | 0.137 | 0.198 | 1.459 | -3.202 | 0.149 | 0.002 | 0.950 |
| rs4147929 | ABCA7 | PI(18:1_18:2) | 0.200 | -0.634 | 0.137 | 0.198 | 1.459 | -3.202 | 0.149 | 0.002 | 0.950 |
| rs983392 | MS4A6A | PC(28:0) | -0.222 | 0.664 | 0.142 | 0.210 | -1.563 | 3.156 | 0.122 | 0.002 | 0.950 |
| rs744373 | BIN1 | TG(16:0_18:1_20:3) | 0.229 | -0.575 | 0.125 | 0.182 | 1.827 | -3.154 | 0.072 | 0.002 | 0.950 |
| rs11136000 | CLU | TG(18:4_16:0_16:1) | 0.304 | -0.640 | 0.137 | 0.205 | 2.212 | -3.117 | 0.030 | 0.003 | 0.950 |
| rs1532278 | CLU | TG(18:4_16:0_16:1) | 0.304 | -0.640 | 0.137 | 0.205 | 2.212 | -3.117 | 0.030 | 0.003 | 0.950 |
| rs3865444 | CD33 | TG(16:0_17:1_18:3) | -0.318 | 0.587 | 0.121 | 0.189 | -2.616 | 3.111 | 0.011 | 0.003 | 0.950 |
| rs3752246 | ABCA7 | TG(17:0_18:1_20:5) | 0.267 | -0.558 | 0.125 | 0.180 | 2.144 | -3.106 | 0.035 | 0.003 | 0.950 |
| rs4147929 | ABCA7 | TG(17:0_18:1_20:5) | 0.267 | -0.558 | 0.125 | 0.180 | 2.144 | -3.106 | 0.035 | 0.003 | 0.950 |
| rs744373 | BIN1 | TG(15:0_15:0_15:0) | -0.168 | 0.653 | 0.146 | 0.212 | -1.154 | 3.077 | 0.252 | 0.003 | 0.950 |
| rs2718058 | NME8 | TG(14:0_18:3_18:3) | 0.058 | -0.605 | 0.125 | 0.198 | 0.463 | -3.054 | 0.645 | 0.003 | 0.950 |
| rs10498633 | SLC24A4 | TG(17:0_18:1_20:4) | 0.369 | -0.618 | 0.162 | 0.203 | 2.274 | -3.052 | 0.026 | 0.003 | 0.950 |
| rs3818361 | CR1 | PE(16:0_18:1) | 0.316 | -0.628 | 0.151 | 0.206 | 2.087 | -3.043 | 0.040 | 0.003 | 0.950 |
| rs6701713 | CR1 | PE(16:0_18:1) | 0.316 | -0.628 | 0.151 | 0.206 | 2.087 | -3.043 | 0.040 | 0.003 | 0.950 |
| rs28834970 | PTK2B | DG(16:0_18:1) | 0.288 | -0.627 | 0.146 | 0.206 | 1.974 | -3.043 | 0.052 | 0.003 | 0.950 |
| rs983392 | MS4A6A | TG(16:0_12:0_17:1) | -0.197 | 0.599 | 0.133 | 0.197 | -1.485 | 3.042 | 0.142 | 0.003 | 0.950 |
| rs744373 | BIN1 | TG(18:1_17:1_18:1) | 0.246 | -0.643 | 0.146 | 0.213 | 1.683 | -3.023 | 0.097 | 0.003 | 0.950 |
| rs6733839 | BIN1 | TG(17:0_18:1_20:5) | 0.145 | -0.551 | 0.119 | 0.182 | 1.215 | -3.020 | 0.228 | 0.003 | 0.950 |
| rs3752246 | ABCA7 | TG(16:1_18:1_18:2) | 0.254 | -0.529 | 0.122 | 0.176 | 2.077 | -2.997 | 0.041 | 0.004 | 0.950 |
| rs4147929 | ABCA7 | TG(16:1_18:1_18:2) | 0.254 | -0.529 | 0.122 | 0.176 | 2.077 | -2.997 | 0.041 | 0.004 | 0.950 |
| rs1131497 | SORL1 | PC(18:2_20:4) | 0.233 | -0.629 | 0.137 | 0.211 | 1.703 | -2.983 | 0.093 | 0.004 | 0.950 |
| rs610932 | MS4A6A | TG(16:0_12:0_17:1) | -0.206 | 0.583 | 0.138 | 0.197 | -1.492 | 2.964 | 0.140 | 0.004 | 0.950 |
| rs3752246 | ABCA7 | TG(18:1_18:2_24:1) | 0.348 | -0.580 | 0.136 | 0.196 | 2.555 | -2.951 | 0.013 | 0.004 | 0.950 |
| rs4147929 | ABCA7 | TG(18:1_18:2_24:1) | 0.348 | -0.580 | 0.136 | 0.196 | 2.555 | -2.951 | 0.013 | 0.004 | 0.950 |
| rs744373 | BIN1 | TG(17:0_18:1_20:5) | 0.199 | -0.534 | 0.125 | 0.182 | 1.588 | -2.935 | 0.116 | 0.004 | 0.950 |
| rs10498633 | SLC24A4 | TG(18:1_17:1_18:1) | 0.355 | -0.653 | 0.179 | 0.223 | 1.989 | -2.932 | 0.050 | 0.004 | 0.950 |
| rs983392 | MS4A6A | TG(16:0_14:0_18:1) | -0.140 | 0.566 | 0.131 | 0.194 | -1.071 | 2.924 | 0.288 | 0.005 | 0.950 |
| rs190982 | MEF2C | TG(18:1_20:2_22:5) | -0.018 | 0.583 | 0.133 | 0.199 | -0.137 | 2.923 | 0.892 | 0.005 | 0.950 |
| rs6733839 | BIN1 | TG(15:0_15:0_15:0) | -0.194 | 0.632 | 0.142 | 0.217 | -1.365 | 2.916 | 0.176 | 0.005 | 0.950 |
| rs744373 | BIN1 | TG(20:0_18:2_18:2) | 0.210 | -0.544 | 0.129 | 0.187 | 1.632 | -2.911 | 0.107 | 0.005 | 0.950 |
| rs1476679 | ZCWPW1 | ChE(20:2) | 0.175 | -0.593 | 0.134 | 0.204 | 1.304 | -2.905 | 0.196 | 0.005 | 0.950 |
| rs10792832 | PICALM | Cer(d18:2_24:1) | -0.064 | 0.565 | 0.131 | 0.195 | -0.488 | 2.897 | 0.627 | 0.005 | 0.950 |
| rs3851179 | PICALM | Cer(d18:2_24:1) | -0.064 | 0.565 | 0.131 | 0.195 | -0.488 | 2.897 | 0.627 | 0.005 | 0.950 |
| rs610932 | MS4A6A | TG(18:4_16:1_18:2) | 0.233 | -0.544 | 0.132 | 0.188 | 1.762 | -2.891 | 0.082 | 0.005 | 0.950 |
| rs9331896 | CLU | TG(19:1_18:0_18:1) | 0.099 | -0.579 | 0.133 | 0.201 | 0.748 | -2.885 | 0.457 | 0.005 | 0.950 |
| rs3818361 | CR1 | PE(16:0_20:3) | 0.340 | -0.592 | 0.152 | 0.207 | 2.236 | -2.861 | 0.028 | 0.005 | 0.950 |
| rs6701713 | CR1 | PE(16:0_20:3) | 0.340 | -0.592 | 0.152 | 0.207 | 2.236 | -2.861 | 0.028 | 0.005 | 0.950 |
| rs983392 | MS4A6A | Cer(d18:1_18:0) | -0.166 | 0.548 | 0.130 | 0.192 | -1.276 | 2.851 | 0.206 | 0.006 | 0.950 |
| rs610932 | MS4A6A | TG(16:0_14:0_18:1) | -0.140 | 0.550 | 0.136 | 0.193 | -1.030 | 2.848 | 0.306 | 0.006 | 0.950 |
| rs983392 | MS4A6A | TG(20:5_14:1_18:2) | 0.176 | -0.570 | 0.135 | 0.200 | 1.302 | -2.844 | 0.197 | 0.006 | 0.950 |
| rs2718058 | NME8 | TG(16:0_16:0_20:5) | 0.036 | -0.579 | 0.128 | 0.204 | 0.282 | -2.843 | 0.779 | 0.006 | 0.950 |
| rs610932 | MS4A6A | TG(16:0_11:1_18:1) | -0.257 | 0.582 | 0.145 | 0.205 | -1.776 | 2.833 | 0.080 | 0.006 | 0.950 |
| rs610932 | MS4A6A | SM(d36:1) | -0.465 | 0.573 | 0.144 | 0.204 | -3.238 | 2.806 | 0.002 | 0.006 | 0.950 |
| rs6656401 | CR1 | PE(16:0_18:1) | 0.301 | -0.582 | 0.146 | 0.207 | 2.069 | -2.805 | 0.042 | 0.006 | 0.950 |
| rs744373 | BIN1 | TG(19:0_18:1_18:1) | 0.196 | -0.502 | 0.123 | 0.179 | 1.596 | -2.803 | 0.115 | 0.006 | 0.950 |
| rs10498633 | SLC24A4 | TG(17:0_18:1_22:4) | 0.457 | -0.600 | 0.172 | 0.214 | 2.662 | -2.801 | 0.010 | 0.006 | 0.950 |
| rs10792832 | PICALM | SM(d44:2) | -0.098 | 0.556 | 0.134 | 0.199 | -0.732 | 2.793 | 0.466 | 0.007 | 0.950 |
| rs3851179 | PICALM | SM(d44:2) | -0.098 | 0.556 | 0.134 | 0.199 | -0.732 | 2.793 | 0.466 | 0.007 | 0.950 |
| rs17125944 | FERMT2 | SM(t38:3) | -0.450 | 0.614 | 0.183 | 0.220 | -2.460 | 2.793 | 0.016 | 0.007 | 0.950 |
| rs2718058 | NME8 | TG(18:4_16:0_16:1) | 0.039 | -0.575 | 0.130 | 0.207 | 0.298 | -2.781 | 0.766 | 0.007 | 0.950 |
| rs17125944 | FERMT2 | PC(18:2_18:2) | -0.384 | 0.614 | 0.184 | 0.221 | -2.084 | 2.774 | 0.041 | 0.007 | 0.950 |
| rs9331896 | CLU | SM(d18:1_18:3) | -0.299 | 0.554 | 0.132 | 0.200 | -2.269 | 2.773 | 0.026 | 0.007 | 0.950 |
| rs6656401 | CR1 | Cer(d18:0_23:0) | 0.264 | -0.535 | 0.135 | 0.193 | 1.948 | -2.772 | 0.055 | 0.007 | 0.950 |
| rs11136000 | CLU | SM(d18:1_18:3) | -0.302 | 0.550 | 0.133 | 0.199 | -2.268 | 2.767 | 0.026 | 0.007 | 0.950 |
| rs1532278 | CLU | SM(d18:1_18:3) | -0.302 | 0.550 | 0.133 | 0.199 | -2.268 | 2.767 | 0.026 | 0.007 | 0.950 |
| rs610932 | MS4A6A | PC(28:0) | -0.227 | 0.588 | 0.150 | 0.213 | -1.513 | 2.762 | 0.135 | 0.007 | 0.950 |
| rs744373 | BIN1 | TG(18:1_18:2_22:0) | 0.241 | -0.542 | 0.135 | 0.196 | 1.790 | -2.760 | 0.078 | 0.007 | 0.950 |
| rs190982 | MEF2C | TG(18:0_16:0_17:1) | 0.083 | -0.573 | 0.138 | 0.208 | 0.602 | -2.759 | 0.549 | 0.007 | 0.950 |
| rs11767557 | EPHA1 | TG(18:1_17:1_18:3) | -0.348 | 0.570 | 0.127 | 0.207 | -2.733 | 2.758 | 0.008 | 0.007 | 0.950 |
| rs561655 | PICALM | SM(d44:2) | -0.101 | 0.550 | 0.136 | 0.200 | -0.745 | 2.757 | 0.458 | 0.007 | 0.950 |
| rs17125944 | FERMT2 | TG(18:1_18:2_23:0) | -0.436 | 0.593 | 0.179 | 0.215 | -2.434 | 2.755 | 0.017 | 0.007 | 0.950 |
| rs610932 | MS4A6A | PC(40:6) | -0.287 | 0.528 | 0.135 | 0.192 | -2.122 | 2.744 | 0.037 | 0.008 | 0.950 |
| rs983392 | MS4A6A | PI(18:1_18:2) | 0.071 | -0.547 | 0.135 | 0.199 | 0.527 | -2.742 | 0.600 | 0.008 | 0.950 |
| rs9331896 | CLU | TG(16:0_16:0_20:5) | 0.171 | -0.565 | 0.136 | 0.206 | 1.259 | -2.742 | 0.212 | 0.008 | 0.950 |
| rs7561528 | BIN1 | TG(16:0_8:0_14:0) | -0.345 | 0.583 | 0.152 | 0.213 | -2.267 | 2.738 | 0.026 | 0.008 | 0.950 |
| rs983392 | MS4A6A | TG(18:4_16:1_18:2) | 0.203 | -0.518 | 0.128 | 0.190 | 1.588 | -2.727 | 0.117 | 0.008 | 0.950 |
| rs983392 | MS4A6A | SM(d32:4) | -0.122 | 0.536 | 0.133 | 0.197 | -0.919 | 2.726 | 0.361 | 0.008 | 0.950 |
| rs17125944 | FERMT2 | TG(19:1_18:0_18:1) | -0.462 | 0.607 | 0.186 | 0.223 | -2.491 | 2.723 | 0.015 | 0.008 | 0.950 |
| rs6656401 | CR1 | Cer(d19:0_23:0) | 0.308 | -0.551 | 0.142 | 0.203 | 2.160 | -2.717 | 0.034 | 0.008 | 0.950 |
| rs17125944 | FERMT2 | PI(18:1_18:2) | -0.438 | 0.609 | 0.187 | 0.224 | -2.347 | 2.715 | 0.022 | 0.008 | 0.950 |
| rs7274581 | CASS4 | SM(d36:1) | -0.371 | 0.570 | 0.141 | 0.210 | -2.624 | 2.715 | 0.011 | 0.008 | 0.950 |
| rs6733839 | BIN1 | TG(16:0_16:0_23:0) | -0.291 | 0.580 | 0.140 | 0.214 | -2.078 | 2.712 | 0.041 | 0.008 | 0.950 |
| rs9331896 | CLU | PE(18:0p_18:1) | -0.206 | 0.585 | 0.143 | 0.217 | -1.439 | 2.702 | 0.154 | 0.009 | 0.950 |
| rs10498633 | SLC24A4 | Cer(d18:0_24:0) | -0.204 | 0.567 | 0.168 | 0.210 | -1.211 | 2.702 | 0.230 | 0.009 | 0.950 |
| rs3818361 | CR1 | PE(18:1p_18:1) | 0.270 | -0.551 | 0.150 | 0.204 | 1.805 | -2.701 | 0.075 | 0.009 | 0.950 |
| rs6701713 | CR1 | PE(18:1p_18:1) | 0.270 | -0.551 | 0.150 | 0.204 | 1.805 | -2.701 | 0.075 | 0.009 | 0.950 |
| rs983392 | MS4A6A | TG(6:0_11:2_18:3) | 0.311 | -0.570 | 0.143 | 0.211 | 2.183 | -2.698 | 0.032 | 0.009 | 0.950 |
| rs744373 | BIN1 | TG(18:1_18:2_22:5) | 0.197 | -0.536 | 0.137 | 0.199 | 1.446 | -2.697 | 0.153 | 0.009 | 0.950 |
| rs983392 | MS4A6A | TG(16:0_11:1_18:1) | -0.216 | 0.558 | 0.140 | 0.207 | -1.548 | 2.696 | 0.126 | 0.009 | 0.950 |
| rs2718058 | NME8 | TG(16:1_14:0_18:3) | 0.083 | -0.557 | 0.130 | 0.207 | 0.634 | -2.685 | 0.528 | 0.009 | 0.950 |
| rs11136000 | CLU | Cer(d18:1_16:0) | -0.300 | 0.518 | 0.130 | 0.194 | -2.312 | 2.670 | 0.024 | 0.009 | 0.950 |
| rs1532278 | CLU | Cer(d18:1_16:0) | -0.300 | 0.518 | 0.130 | 0.194 | -2.312 | 2.670 | 0.024 | 0.009 | 0.950 |
| rs3865444 | CD33 | DG(35:3e) | 0.283 | -0.582 | 0.140 | 0.218 | 2.015 | -2.669 | 0.048 | 0.009 | 0.950 |
| rs11136000 | CLU | TG(19:1_18:0_18:1) | 0.100 | -0.537 | 0.135 | 0.202 | 0.741 | -2.662 | 0.461 | 0.010 | 0.950 |
| rs1532278 | CLU | TG(19:1_18:0_18:1) | 0.100 | -0.537 | 0.135 | 0.202 | 0.741 | -2.662 | 0.461 | 0.010 | 0.950 |
| rs6733839 | BIN1 | TG(20:0_18:2_18:2) | 0.195 | -0.507 | 0.125 | 0.191 | 1.561 | -2.661 | 0.123 | 0.010 | 0.950 |
| rs1476679 | ZCWPW1 | SM(t36:1) | 0.226 | -0.566 | 0.140 | 0.213 | 1.619 | -2.660 | 0.110 | 0.010 | 0.950 |
| rs610932 | MS4A6A | PC(20:2_18:2) | -0.178 | 0.493 | 0.131 | 0.186 | -1.358 | 2.651 | 0.179 | 0.010 | 0.950 |
| rs7274581 | CASS4 | PC(36:3) | 0.087 | -0.535 | 0.136 | 0.203 | 0.635 | -2.643 | 0.528 | 0.010 | 0.950 |
| rs3865444 | CD33 | TG(15:0_16:0_16:1) | -0.110 | 0.570 | 0.139 | 0.216 | -0.793 | 2.643 | 0.430 | 0.010 | 0.950 |
| rs6733839 | BIN1 | TG(19:0_18:1_18:1) | 0.147 | -0.479 | 0.119 | 0.181 | 1.234 | -2.642 | 0.221 | 0.010 | 0.950 |
| rs610932 | MS4A6A | TG(18:0_12:1_16:0) | -0.144 | 0.549 | 0.146 | 0.208 | -0.985 | 2.642 | 0.328 | 0.010 | 0.950 |
| rs7561528 | BIN1 | PE(20:0p_22:6) | 0.288 | -0.564 | 0.153 | 0.214 | 1.885 | -2.639 | 0.063 | 0.010 | 0.950 |
| rs10792832 | PICALM | TG(29:0_18:1_18:1) | -0.210 | 0.535 | 0.137 | 0.204 | -1.535 | 2.627 | 0.129 | 0.010 | 0.950 |
| rs3851179 | PICALM | TG(29:0_18:1_18:1) | -0.210 | 0.535 | 0.137 | 0.204 | -1.535 | 2.627 | 0.129 | 0.010 | 0.950 |
| rs983392 | MS4A6A | TG(16:0_14:0_16:0) | -0.175 | 0.539 | 0.139 | 0.205 | -1.266 | 2.625 | 0.210 | 0.011 | 0.950 |
| rs9331896 | CLU | TG(18:4_16:0_20:4) | 0.127 | -0.548 | 0.138 | 0.209 | 0.920 | -2.622 | 0.361 | 0.011 | 0.950 |
| rs9331896 | CLU | Cer(d18:1_16:0) | -0.298 | 0.512 | 0.129 | 0.195 | -2.310 | 2.622 | 0.024 | 0.011 | 0.950 |
| rs744373 | BIN1 | TG(20:5_14:1_18:2) | 0.220 | -0.533 | 0.140 | 0.203 | 1.576 | -2.621 | 0.119 | 0.011 | 0.950 |
| rs983392 | MS4A6A | PI(18:0_20:4) | 0.184 | -0.521 | 0.134 | 0.199 | 1.369 | -2.616 | 0.175 | 0.011 | 0.950 |
| rs7561528 | BIN1 | PI(16:0_18:1) | -0.496 | 0.546 | 0.149 | 0.209 | -3.325 | 2.615 | 0.001 | 0.011 | 0.950 |
| rs7274581 | CASS4 | TG(18:1_20:2_22:5) | 0.100 | -0.537 | 0.138 | 0.205 | 0.723 | -2.615 | 0.472 | 0.011 | 0.950 |
| rs9331896 | CLU | TG(16:0_14:0_18:3) | 0.026 | -0.514 | 0.130 | 0.197 | 0.197 | -2.613 | 0.845 | 0.011 | 0.950 |
| rs983392 | MS4A6A | TG(33:4e) | 0.278 | -0.538 | 0.139 | 0.206 | 1.994 | -2.609 | 0.050 | 0.011 | 0.950 |
| rs10498633 | SLC24A4 | TG(18:0_20:4_22:5) | 0.333 | -0.589 | 0.181 | 0.226 | 1.839 | -2.606 | 0.070 | 0.011 | 0.950 |
| rs561655 | PICALM | PC(22:0_11:3) | 0.273 | -0.568 | 0.148 | 0.218 | 1.840 | -2.606 | 0.070 | 0.011 | 0.950 |
| rs3818361 | CR1 | Cer(d18:0_23:0) | 0.277 | -0.507 | 0.143 | 0.195 | 1.937 | -2.603 | 0.057 | 0.011 | 0.950 |
| rs6701713 | CR1 | Cer(d18:0_23:0) | 0.277 | -0.507 | 0.143 | 0.195 | 1.937 | -2.603 | 0.057 | 0.011 | 0.950 |
| rs561655 | PICALM | TG(18:0_16:0_19:0) | -0.407 | 0.513 | 0.134 | 0.197 | -3.035 | 2.602 | 0.003 | 0.011 | 0.950 |
| rs6733839 | BIN1 | DG(22:4e) | 0.125 | -0.533 | 0.135 | 0.205 | 0.930 | -2.596 | 0.355 | 0.011 | 0.950 |
| rs983392 | MS4A6A | TG(18:1_12:0_14:0) | -0.168 | 0.529 | 0.138 | 0.204 | -1.218 | 2.595 | 0.227 | 0.011 | 0.950 |
| rs11136000 | CLU | PE(18:0p_18:1) | -0.207 | 0.561 | 0.145 | 0.216 | -1.434 | 2.595 | 0.156 | 0.011 | 0.950 |
| rs1532278 | CLU | PE(18:0p_18:1) | -0.207 | 0.561 | 0.145 | 0.216 | -1.434 | 2.595 | 0.156 | 0.011 | 0.950 |
| rs983392 | MS4A6A | SM(d42:2) | -0.059 | 0.492 | 0.128 | 0.190 | -0.462 | 2.591 | 0.646 | 0.012 | 0.950 |
| rs3818361 | CR1 | PI(18:1_20:4) | 0.136 | -0.506 | 0.144 | 0.196 | 0.945 | -2.580 | 0.348 | 0.012 | 0.950 |
| rs6701713 | CR1 | PI(18:1_20:4) | 0.136 | -0.506 | 0.144 | 0.196 | 0.945 | -2.580 | 0.348 | 0.012 | 0.950 |
| rs10498633 | SLC24A4 | TG(29:0_16:0_16:0) | 0.339 | -0.558 | 0.173 | 0.216 | 1.956 | -2.580 | 0.054 | 0.012 | 0.950 |
| rs3818361 | CR1 | Cer(d19:0_23:0) | 0.323 | -0.527 | 0.150 | 0.205 | 2.150 | -2.574 | 0.035 | 0.012 | 0.950 |
| rs6701713 | CR1 | Cer(d19:0_23:0) | 0.323 | -0.527 | 0.150 | 0.205 | 2.150 | -2.574 | 0.035 | 0.012 | 0.950 |
| rs983392 | MS4A6A | TG(18:0_12:1_16:0) | -0.121 | 0.538 | 0.141 | 0.209 | -0.854 | 2.574 | 0.396 | 0.012 | 0.950 |
| rs983392 | MS4A6A | SM(d35:1) | -0.218 | 0.546 | 0.143 | 0.212 | -1.523 | 2.573 | 0.132 | 0.012 | 0.950 |
| rs744373 | BIN1 | TG(20:0_18:1_18:1) | 0.216 | -0.486 | 0.130 | 0.189 | 1.659 | -2.570 | 0.101 | 0.012 | 0.950 |
| rs744373 | BIN1 | TG(18:1_18:1_21:1) | 0.129 | -0.524 | 0.140 | 0.204 | 0.924 | -2.568 | 0.359 | 0.012 | 0.950 |
| rs610932 | MS4A6A | TG(18:1_12:0_14:0) | -0.188 | 0.522 | 0.143 | 0.203 | -1.315 | 2.567 | 0.192 | 0.012 | 0.950 |
| rs11218343 | SORL1 | PC(18:2_20:4) | -0.374 | 0.570 | 0.178 | 0.222 | -2.100 | 2.567 | 0.039 | 0.012 | 0.950 |
| rs7274581 | CASS4 | DG(36:5e) | 0.266 | -0.474 | 0.124 | 0.185 | 2.142 | -2.566 | 0.036 | 0.012 | 0.950 |
| rs11218343 | SORL1 | TG(15:0_18:2_18:2) | -0.257 | 0.528 | 0.165 | 0.206 | -1.553 | 2.566 | 0.125 | 0.012 | 0.950 |
| rs3752246 | ABCA7 | TG(16:1_20:1_20:1) | 0.280 | -0.512 | 0.138 | 0.199 | 2.027 | -2.565 | 0.046 | 0.012 | 0.950 |
| rs4147929 | ABCA7 | TG(16:1_20:1_20:1) | 0.280 | -0.512 | 0.138 | 0.199 | 2.027 | -2.565 | 0.046 | 0.012 | 0.950 |
| rs11136000 | CLU | SM(d18:0_16:1) | -0.233 | 0.484 | 0.126 | 0.189 | -1.847 | 2.564 | 0.069 | 0.012 | 0.950 |
| rs1532278 | CLU | SM(d18:0_16:1) | -0.233 | 0.484 | 0.126 | 0.189 | -1.847 | 2.564 | 0.069 | 0.012 | 0.950 |
| rs983392 | MS4A6A | PC(31:0) | -0.267 | 0.535 | 0.141 | 0.209 | -1.896 | 2.564 | 0.062 | 0.012 | 0.950 |
| rs983392 | MS4A6A | SM(d42:1) | -0.144 | 0.508 | 0.134 | 0.198 | -1.073 | 2.560 | 0.287 | 0.013 | 0.950 |
| rs2718058 | NME8 | TG(18:1_20:4_22:5) | 0.185 | -0.548 | 0.135 | 0.215 | 1.369 | -2.552 | 0.175 | 0.013 | 0.950 |
| rs744373 | BIN1 | TG(16:1_20:1_22:4) | 0.130 | -0.502 | 0.135 | 0.197 | 0.959 | -2.548 | 0.341 | 0.013 | 0.950 |
| rs744373 | BIN1 | DG(22:4e) | 0.173 | -0.520 | 0.141 | 0.205 | 1.230 | -2.540 | 0.223 | 0.013 | 0.950 |
| rs744373 | BIN1 | TG(18:1_20:2_22:5) | 0.117 | -0.520 | 0.141 | 0.205 | 0.830 | -2.537 | 0.409 | 0.013 | 0.950 |
| rs983392 | MS4A6A | Cer(m18:0_24:1) | 0.019 | 0.485 | 0.129 | 0.191 | 0.145 | 2.535 | 0.885 | 0.013 | 0.950 |
| rs2718058 | NME8 | TG(12:0_17:1_18:2) | 0.304 | -0.538 | 0.134 | 0.213 | 2.270 | -2.532 | 0.026 | 0.013 | 0.950 |
| rs610932 | MS4A6A | PC(42:7) | -0.307 | 0.522 | 0.145 | 0.206 | -2.111 | 2.531 | 0.038 | 0.013 | 0.950 |
| rs610932 | MS4A6A | PI(18:0_20:4) | 0.148 | -0.501 | 0.140 | 0.198 | 1.060 | -2.529 | 0.292 | 0.014 | 0.950 |
| rs1131497 | SORL1 | SM(d17:1_13:0) | -0.162 | 0.541 | 0.139 | 0.214 | -1.163 | 2.528 | 0.248 | 0.014 | 0.950 |
| rs17125944 | FERMT2 | Cer(m18:1_22:0) | -0.280 | 0.557 | 0.184 | 0.221 | -1.525 | 2.524 | 0.132 | 0.014 | 0.950 |
| rs11136000 | CLU | TG(18:1_14:0_18:2) | 0.139 | -0.512 | 0.136 | 0.203 | 1.027 | -2.523 | 0.308 | 0.014 | 0.950 |
| rs1532278 | CLU | TG(18:1_14:0_18:2) | 0.139 | -0.512 | 0.136 | 0.203 | 1.027 | -2.523 | 0.308 | 0.014 | 0.950 |
| rs11136000 | CLU | TG(16:0_16:0_20:5) | 0.173 | -0.522 | 0.138 | 0.207 | 1.248 | -2.522 | 0.216 | 0.014 | 0.950 |
| rs1532278 | CLU | TG(16:0_16:0_20:5) | 0.173 | -0.522 | 0.138 | 0.207 | 1.248 | -2.522 | 0.216 | 0.014 | 0.950 |
| rs610932 | MS4A6A | Cer(d18:1_18:0) | -0.081 | 0.483 | 0.135 | 0.191 | -0.598 | 2.522 | 0.551 | 0.014 | 0.950 |
| rs3752246 | ABCA7 | PE(18:1e) | 0.141 | -0.514 | 0.142 | 0.204 | 0.997 | -2.520 | 0.322 | 0.014 | 0.950 |
| rs4147929 | ABCA7 | PE(18:1e) | 0.141 | -0.514 | 0.142 | 0.204 | 0.997 | -2.520 | 0.322 | 0.014 | 0.950 |
| rs10498633 | SLC24A4 | TG(18:1_20:3_20:3) | 0.300 | -0.560 | 0.178 | 0.222 | 1.681 | -2.519 | 0.097 | 0.014 | 0.950 |
| rs2718058 | NME8 | SM(d28:1) | -0.399 | 0.516 | 0.129 | 0.205 | -3.095 | 2.517 | 0.003 | 0.014 | 0.950 |
| rs9349407 | CD2AP | SM(d37:2) | 0.254 | -0.508 | 0.144 | 0.202 | 1.763 | -2.516 | 0.082 | 0.014 | 0.950 |
| rs10948363 | CD2AP | SM(d37:2) | 0.254 | -0.508 | 0.144 | 0.202 | 1.763 | -2.516 | 0.082 | 0.014 | 0.950 |
| rs610932 | MS4A6A | PI(16:0_20:3) | -0.098 | -0.458 | 0.128 | 0.182 | -0.762 | -2.513 | 0.448 | 0.014 | 0.950 |
| rs11218343 | SORL1 | ChE(22:5) | -0.244 | 0.533 | 0.171 | 0.213 | -1.427 | 2.505 | 0.158 | 0.014 | 0.950 |
| rs9331896 | CLU | SM(d18:0_16:1) | -0.231 | 0.475 | 0.125 | 0.190 | -1.844 | 2.502 | 0.069 | 0.015 | 0.950 |
| rs983392 | MS4A6A | PC(36:1) | -0.114 | 0.476 | 0.129 | 0.191 | -0.882 | 2.488 | 0.381 | 0.015 | 0.950 |
| rs3865444 | CD33 | TG(14:0_18:2_20:5) | 0.247 | -0.481 | 0.124 | 0.193 | 1.982 | -2.488 | 0.051 | 0.015 | 0.950 |
| rs983392 | MS4A6A | TG(17:0_18:1_20:3) | 0.077 | -0.417 | 0.113 | 0.168 | 0.680 | -2.485 | 0.498 | 0.015 | 0.950 |
| rs3865444 | CD33 | TG(15:0_15:0_15:0) | -0.019 | 0.535 | 0.139 | 0.216 | -0.135 | 2.481 | 0.893 | 0.015 | 0.950 |
| rs11136000 | CLU | SM(d42:2) | -0.139 | 0.482 | 0.130 | 0.195 | -1.065 | 2.478 | 0.290 | 0.015 | 0.950 |
| rs1532278 | CLU | SM(d42:2) | -0.139 | 0.482 | 0.130 | 0.195 | -1.065 | 2.478 | 0.290 | 0.015 | 0.950 |
| rs11136000 | CLU | SM(d44:4) | -0.230 | 0.498 | 0.135 | 0.201 | -1.709 | 2.474 | 0.092 | 0.016 | 0.950 |
| rs1532278 | CLU | SM(d44:4) | -0.230 | 0.498 | 0.135 | 0.201 | -1.709 | 2.474 | 0.092 | 0.016 | 0.950 |
| rs3752246 | ABCA7 | TG(51:3) | 0.169 | -0.484 | 0.136 | 0.196 | 1.248 | -2.474 | 0.216 | 0.016 | 0.950 |
| rs4147929 | ABCA7 | TG(51:3) | 0.169 | -0.484 | 0.136 | 0.196 | 1.248 | -2.474 | 0.216 | 0.016 | 0.950 |
| rs3752246 | ABCA7 | TG(18:1_18:2_23:1) | 0.187 | -0.500 | 0.140 | 0.202 | 1.331 | -2.472 | 0.187 | 0.016 | 0.950 |
| rs4147929 | ABCA7 | TG(18:1_18:2_23:1) | 0.187 | -0.500 | 0.140 | 0.202 | 1.331 | -2.472 | 0.187 | 0.016 | 0.950 |
| rs744373 | BIN1 | TG(18:1_18:1_22:5) | 0.239 | -0.490 | 0.136 | 0.198 | 1.751 | -2.469 | 0.084 | 0.016 | 0.950 |
| rs1476679 | ZCWPW1 | DG(20:0_18:1) | -0.278 | 0.462 | 0.123 | 0.187 | -2.262 | 2.468 | 0.027 | 0.016 | 0.950 |
| rs10498633 | SLC24A4 | ChE(22:5) | -0.138 | 0.516 | 0.168 | 0.209 | -0.822 | 2.466 | 0.414 | 0.016 | 0.950 |
| rs10792832 | PICALM | TG(16:0e_18:0_20:1) | -0.274 | 0.532 | 0.145 | 0.216 | -1.892 | 2.464 | 0.062 | 0.016 | 0.950 |
| rs3851179 | PICALM | TG(16:0e_18:0_20:1) | -0.274 | 0.532 | 0.145 | 0.216 | -1.892 | 2.464 | 0.062 | 0.016 | 0.950 |
| rs1476679 | ZCWPW1 | DG(20:0_18:2) | -0.202 | 0.441 | 0.118 | 0.179 | -1.719 | 2.464 | 0.090 | 0.016 | 0.950 |
| rs17125944 | FERMT2 | ChE(22:4) | 0.254 | -0.525 | 0.177 | 0.213 | 1.429 | -2.463 | 0.157 | 0.016 | 0.950 |
| rs561655 | PICALM | Cer(d18:2_24:1) | -0.077 | 0.491 | 0.136 | 0.200 | -0.563 | 2.459 | 0.575 | 0.016 | 0.950 |
| rs744373 | BIN1 | TG(16:0_16:0_16:0) | -0.104 | 0.508 | 0.142 | 0.207 | -0.732 | 2.459 | 0.466 | 0.016 | 0.950 |
| rs9331896 | CLU | SM(d44:4) | -0.228 | 0.498 | 0.134 | 0.203 | -1.708 | 2.459 | 0.092 | 0.016 | 0.950 |
| rs610932 | MS4A6A | TG(16:0_17:0_20:4) | -0.088 | 0.463 | 0.133 | 0.189 | -0.658 | 2.452 | 0.512 | 0.017 | 0.950 |
| rs9331896 | CLU | SM(d42:2) | -0.137 | 0.480 | 0.129 | 0.196 | -1.063 | 2.451 | 0.291 | 0.017 | 0.950 |
| rs1476679 | ZCWPW1 | DG(36:3) | -0.187 | 0.460 | 0.123 | 0.188 | -1.518 | 2.449 | 0.133 | 0.017 | 0.950 |
| rs610932 | MS4A6A | SM(d32:4) | -0.140 | 0.485 | 0.140 | 0.198 | -1.003 | 2.446 | 0.319 | 0.017 | 0.950 |
| rs11136000 | CLU | Cer(d16:1_22:0) | -0.213 | 0.476 | 0.130 | 0.195 | -1.635 | 2.445 | 0.106 | 0.017 | 0.950 |
| rs1532278 | CLU | Cer(d16:1_22:0) | -0.213 | 0.476 | 0.130 | 0.195 | -1.635 | 2.445 | 0.106 | 0.017 | 0.950 |
| rs11218343 | SORL1 | PC(38:7) | -0.468 | 0.541 | 0.178 | 0.221 | -2.632 | 2.442 | 0.010 | 0.017 | 0.950 |
| rs9331896 | CLU | TG(18:1_14:0_18:2) | 0.138 | -0.500 | 0.135 | 0.205 | 1.023 | -2.440 | 0.309 | 0.017 | 0.950 |
| rs3865444 | CD33 | TG(20:0_18:1_20:4) | -0.033 | 0.509 | 0.135 | 0.209 | -0.246 | 2.435 | 0.807 | 0.017 | 0.950 |
| rs2718058 | NME8 | TG(22:5_18:2_18:2) | 0.208 | -0.532 | 0.137 | 0.218 | 1.516 | -2.435 | 0.134 | 0.017 | 0.950 |
| rs10792832 | PICALM | TG(28:0_18:1_18:1) | -0.289 | 0.508 | 0.140 | 0.209 | -2.057 | 2.431 | 0.043 | 0.017 | 0.950 |
| rs3851179 | PICALM | TG(28:0_18:1_18:1) | -0.289 | 0.508 | 0.140 | 0.209 | -2.057 | 2.431 | 0.043 | 0.017 | 0.950 |
| rs6656401 | CR1 | PE(16:0_20:3) | 0.324 | -0.506 | 0.146 | 0.208 | 2.211 | -2.426 | 0.030 | 0.018 | 0.950 |
| rs983392 | MS4A6A | ChE(18:3) | -0.107 | 0.448 | 0.125 | 0.185 | -0.861 | 2.424 | 0.392 | 0.018 | 0.950 |
| rs11218343 | SORL1 | TG(16:0_14:1_16:1) | -0.258 | 0.543 | 0.180 | 0.224 | -1.434 | 2.423 | 0.156 | 0.018 | 0.950 |
| rs17125944 | FERMT2 | PE(18:1e) | -0.283 | 0.549 | 0.189 | 0.227 | -1.497 | 2.421 | 0.139 | 0.018 | 0.950 |
| rs610932 | MS4A6A | PC(36:1) | -0.109 | 0.461 | 0.134 | 0.191 | -0.816 | 2.418 | 0.417 | 0.018 | 0.950 |
| rs561655 | PICALM | Cer(d18:2_24:0) | -0.110 | 0.476 | 0.134 | 0.197 | -0.818 | 2.415 | 0.416 | 0.018 | 0.950 |
| rs1476679 | ZCWPW1 | TG(16:0_16:0_18:3) | 0.352 | -0.484 | 0.132 | 0.201 | 2.669 | -2.413 | 0.009 | 0.018 | 0.950 |
| rs3865444 | CD33 | TG(15:0_16:0_16:0) | -0.134 | 0.530 | 0.142 | 0.220 | -0.948 | 2.410 | 0.346 | 0.018 | 0.950 |
| rs3752246 | ABCA7 | DG(20:0_18:2) | 0.246 | -0.426 | 0.123 | 0.177 | 2.004 | -2.408 | 0.049 | 0.019 | 0.950 |
| rs4147929 | ABCA7 | DG(20:0_18:2) | 0.246 | -0.426 | 0.123 | 0.177 | 2.004 | -2.408 | 0.049 | 0.019 | 0.950 |
| rs11136000 | CLU | Cer(d18:1_22:0) | -0.228 | 0.458 | 0.127 | 0.190 | -1.792 | 2.407 | 0.077 | 0.019 | 0.950 |
| rs1532278 | CLU | Cer(d18:1_22:0) | -0.228 | 0.458 | 0.127 | 0.190 | -1.792 | 2.407 | 0.077 | 0.019 | 0.950 |
| rs3818361 | CR1 | PE(18:0_18:1) | 0.293 | -0.496 | 0.152 | 0.207 | 1.928 | -2.401 | 0.058 | 0.019 | 0.950 |
| rs6701713 | CR1 | PE(18:0_18:1) | 0.293 | -0.496 | 0.152 | 0.207 | 1.928 | -2.401 | 0.058 | 0.019 | 0.950 |
| rs7274581 | CASS4 | SM(t36:1) | -0.235 | 0.516 | 0.145 | 0.215 | -1.622 | 2.400 | 0.109 | 0.019 | 0.950 |
| rs7274581 | CASS4 | PC(18:2_18:2) | 0.167 | -0.492 | 0.138 | 0.205 | 1.213 | -2.399 | 0.229 | 0.019 | 0.950 |
| rs11218343 | SORL1 | PE(18:0p_20:4) | -0.393 | 0.551 | 0.184 | 0.230 | -2.131 | 2.398 | 0.036 | 0.019 | 0.950 |
| rs610932 | MS4A6A | TG(16:0_14:0_16:0) | -0.143 | 0.492 | 0.145 | 0.205 | -0.986 | 2.396 | 0.327 | 0.019 | 0.950 |
| rs10792832 | PICALM | SM(d35:4) | 0.013 | 0.472 | 0.132 | 0.197 | 0.100 | 2.395 | 0.921 | 0.019 | 0.950 |
| rs3851179 | PICALM | SM(d35:4) | 0.013 | 0.472 | 0.132 | 0.197 | 0.100 | 2.395 | 0.921 | 0.019 | 0.950 |
| rs983392 | MS4A6A | TG(16:0_17:0_20:4) | -0.053 | 0.453 | 0.128 | 0.189 | -0.414 | 2.393 | 0.680 | 0.019 | 0.950 |
| rs11218343 | SORL1 | TG(16:0_14:0_18:3) | -0.343 | 0.504 | 0.169 | 0.211 | -2.030 | 2.392 | 0.046 | 0.019 | 0.950 |
| rs3752246 | ABCA7 | DG(36:3) | 0.230 | -0.445 | 0.129 | 0.186 | 1.788 | -2.392 | 0.078 | 0.019 | 0.950 |
| rs4147929 | ABCA7 | DG(36:3) | 0.230 | -0.445 | 0.129 | 0.186 | 1.788 | -2.392 | 0.078 | 0.019 | 0.950 |
| rs1131497 | SORL1 | TG(16:0_20:4_22:6) | 0.401 | -0.490 | 0.133 | 0.205 | 3.016 | -2.392 | 0.004 | 0.019 | 0.950 |
| rs983392 | MS4A6A | PC(40:6) | -0.224 | 0.467 | 0.132 | 0.195 | -1.699 | 2.392 | 0.094 | 0.019 | 0.950 |
| rs561655 | PICALM | TG(16:0e_18:0_20:1) | -0.363 | 0.513 | 0.146 | 0.215 | -2.482 | 2.391 | 0.015 | 0.019 | 0.950 |
| rs11136000 | CLU | SM(d16:1_20:0) | -0.189 | 0.450 | 0.126 | 0.189 | -1.499 | 2.388 | 0.138 | 0.019 | 0.950 |
| rs1532278 | CLU | SM(d16:1_20:0) | -0.189 | 0.450 | 0.126 | 0.189 | -1.499 | 2.388 | 0.138 | 0.019 | 0.950 |
| rs1476679 | ZCWPW1 | SM(t39:6) | 0.187 | -0.505 | 0.139 | 0.212 | 1.342 | -2.385 | 0.184 | 0.020 | 0.950 |
| rs744373 | BIN1 | TG(18:1_14:0_18:2) | 0.160 | -0.486 | 0.140 | 0.204 | 1.140 | -2.384 | 0.258 | 0.020 | 0.950 |
| rs3865444 | CD33 | TG(17:0_17:1_17:1) | -0.055 | 0.495 | 0.134 | 0.208 | -0.408 | 2.382 | 0.685 | 0.020 | 0.950 |
| rs9331896 | CLU | TG(18:1_22:5_22:6) | 0.310 | -0.508 | 0.141 | 0.213 | 2.205 | -2.380 | 0.031 | 0.020 | 0.950 |
| rs9331896 | CLU | TG(16:1_20:1_22:4) | 0.186 | -0.478 | 0.132 | 0.201 | 1.406 | -2.379 | 0.164 | 0.020 | 0.950 |
| rs983392 | MS4A6A | PI(18:1_20:4) | 0.023 | -0.463 | 0.131 | 0.195 | 0.175 | -2.376 | 0.862 | 0.020 | 0.950 |
| rs9349407 | CD2AP | TG(17:0_18:1_22:4) | 0.299 | -0.495 | 0.149 | 0.208 | 2.010 | -2.375 | 0.048 | 0.020 | 0.950 |
| rs10948363 | CD2AP | TG(17:0_18:1_22:4) | 0.299 | -0.495 | 0.149 | 0.208 | 2.010 | -2.375 | 0.048 | 0.020 | 0.950 |
| rs10498633 | SLC24A4 | SM(t34:1) | -0.370 | 0.506 | 0.171 | 0.213 | -2.168 | 2.374 | 0.033 | 0.020 | 0.950 |
| rs6733839 | BIN1 | TG(18:1_18:1_22:5) | 0.142 | -0.475 | 0.131 | 0.200 | 1.084 | -2.369 | 0.282 | 0.020 | 0.950 |
| rs11218343 | SORL1 | TG(17:0_18:2_22:6) | -0.392 | 0.539 | 0.183 | 0.228 | -2.143 | 2.366 | 0.035 | 0.021 | 0.950 |
| rs10498633 | SLC24A4 | TG(16:0_18:1_18:2) | 0.198 | -0.501 | 0.170 | 0.212 | 1.165 | -2.362 | 0.248 | 0.021 | 0.950 |
| rs610932 | MS4A6A | TG(20:5_14:1_18:2) | 0.148 | -0.478 | 0.143 | 0.203 | 1.039 | -2.358 | 0.302 | 0.021 | 0.950 |
| rs983392 | MS4A6A | TG(15:0_16:0_16:0) | -0.162 | 0.514 | 0.147 | 0.218 | -1.105 | 2.358 | 0.273 | 0.021 | 0.950 |
| rs9331896 | CLU | Cer(d16:1_22:0) | -0.211 | 0.463 | 0.130 | 0.196 | -1.631 | 2.356 | 0.107 | 0.021 | 0.950 |
| rs10792832 | PICALM | Cer(d18:1_25:0) | -0.013 | 0.479 | 0.136 | 0.203 | -0.099 | 2.356 | 0.922 | 0.021 | 0.950 |
| rs3851179 | PICALM | Cer(d18:1_25:0) | -0.013 | 0.479 | 0.136 | 0.203 | -0.099 | 2.356 | 0.922 | 0.021 | 0.950 |
| rs561655 | PICALM | SM(d18:2_16:0) | -0.031 | 0.445 | 0.128 | 0.189 | -0.244 | 2.356 | 0.808 | 0.021 | 0.950 |
| rs17125944 | FERMT2 | TG(60:4e) | -0.414 | 0.559 | 0.198 | 0.237 | -2.097 | 2.356 | 0.039 | 0.021 | 0.950 |
| rs11136000 | CLU | SM(d33:1) | -0.171 | 0.489 | 0.139 | 0.208 | -1.235 | 2.356 | 0.221 | 0.021 | 0.950 |
| rs1532278 | CLU | SM(d33:1) | -0.171 | 0.489 | 0.139 | 0.208 | -1.235 | 2.356 | 0.221 | 0.021 | 0.950 |
| rs11136000 | CLU | SM(d40:1) | -0.122 | 0.471 | 0.134 | 0.200 | -0.910 | 2.355 | 0.366 | 0.021 | 0.950 |
| rs1532278 | CLU | SM(d40:1) | -0.122 | 0.471 | 0.134 | 0.200 | -0.910 | 2.355 | 0.366 | 0.021 | 0.950 |
| rs11771145 | EPHA1 | TG(33:4e) | 0.120 | -0.485 | 0.144 | 0.206 | 0.830 | -2.354 | 0.409 | 0.021 | 0.950 |
| rs17125944 | FERMT2 | TG(14:0_18:3_18:3) | -0.264 | 0.522 | 0.185 | 0.222 | -1.428 | 2.354 | 0.158 | 0.021 | 0.950 |
| rs10792832 | PICALM | Cer(m18:1_22:0) | -0.101 | 0.474 | 0.135 | 0.201 | -0.749 | 2.353 | 0.456 | 0.021 | 0.950 |
| rs3851179 | PICALM | Cer(m18:1_22:0) | -0.101 | 0.474 | 0.135 | 0.201 | -0.749 | 2.353 | 0.456 | 0.021 | 0.950 |
| rs983392 | MS4A6A | TG(17:0_17:1_19:0) | -0.153 | 0.471 | 0.135 | 0.200 | -1.128 | 2.351 | 0.263 | 0.021 | 0.950 |
| rs1476679 | ZCWPW1 | DG(35:3e) | -0.448 | 0.500 | 0.140 | 0.213 | -3.204 | 2.349 | 0.002 | 0.021 | 0.950 |
| rs983392 | MS4A6A | TG(16:1_20:1_20:1) | 0.137 | -0.472 | 0.136 | 0.201 | 1.011 | -2.349 | 0.315 | 0.021 | 0.950 |
| rs610932 | MS4A6A | TG(16:0_16:0_17:0) | -0.082 | 0.497 | 0.149 | 0.212 | -0.550 | 2.346 | 0.584 | 0.022 | 0.950 |
| rs10498633 | SLC24A4 | TG(18:1_18:2_20:3) | 0.306 | -0.526 | 0.180 | 0.224 | 1.702 | -2.345 | 0.093 | 0.022 | 0.950 |
| rs1476679 | ZCWPW1 | SM(d18:1_24:0) | 0.123 | -0.492 | 0.138 | 0.210 | 0.891 | -2.343 | 0.376 | 0.022 | 0.950 |
| rs11136000 | CLU | SM(d38:2) | -0.149 | 0.477 | 0.136 | 0.204 | -1.093 | 2.340 | 0.278 | 0.022 | 0.950 |
| rs1532278 | CLU | SM(d38:2) | -0.149 | 0.477 | 0.136 | 0.204 | -1.093 | 2.340 | 0.278 | 0.022 | 0.950 |
| rs6733839 | BIN1 | TG(15:0_16:0_18:3) | -0.233 | 0.505 | 0.141 | 0.216 | -1.646 | 2.339 | 0.104 | 0.022 | 0.950 |
| rs983392 | MS4A6A | SM(t18:0_24:2) | -0.273 | 0.487 | 0.141 | 0.208 | -1.942 | 2.339 | 0.056 | 0.022 | 0.950 |
| rs190982 | MEF2C | TG(18:1_22:5_22:6) | -0.229 | 0.503 | 0.143 | 0.215 | -1.598 | 2.338 | 0.114 | 0.022 | 0.950 |
| rs3752246 | ABCA7 | DG(16:1_18:2) | 0.186 | -0.451 | 0.134 | 0.193 | 1.393 | -2.338 | 0.168 | 0.022 | 0.950 |
| rs4147929 | ABCA7 | DG(16:1_18:2) | 0.186 | -0.451 | 0.134 | 0.193 | 1.393 | -2.338 | 0.168 | 0.022 | 0.950 |
| rs983392 | MS4A6A | DG(22:4e) | 0.030 | -0.471 | 0.136 | 0.201 | 0.221 | -2.336 | 0.826 | 0.022 | 0.950 |
| rs7274581 | CASS4 | SM(d42:1) | -0.350 | 0.468 | 0.135 | 0.200 | -2.600 | 2.336 | 0.011 | 0.022 | 0.950 |
| rs3752246 | ABCA7 | TG(29:0_18:1_18:1) | 0.220 | -0.477 | 0.142 | 0.205 | 1.553 | -2.332 | 0.125 | 0.022 | 0.950 |
| rs4147929 | ABCA7 | TG(29:0_18:1_18:1) | 0.220 | -0.477 | 0.142 | 0.205 | 1.553 | -2.332 | 0.125 | 0.022 | 0.950 |
| rs744373 | BIN1 | TG(18:1_18:2_18:2) | 0.168 | -0.451 | 0.133 | 0.193 | 1.265 | -2.331 | 0.210 | 0.022 | 0.950 |
| rs610932 | MS4A6A | TG(16:0_16:0_16:0) | -0.102 | 0.480 | 0.145 | 0.206 | -0.706 | 2.329 | 0.482 | 0.023 | 0.950 |
| rs7274581 | CASS4 | TG(60:6) | 0.125 | -0.481 | 0.139 | 0.207 | 0.902 | -2.329 | 0.370 | 0.023 | 0.950 |
| rs35349669 | INPP5D | TG(18:1_11:1_18:1) | -0.276 | 0.508 | 0.156 | 0.218 | -1.772 | 2.327 | 0.081 | 0.023 | 0.950 |
| rs10498633 | SLC24A4 | TG(30:0_16:0_18:1) | 0.479 | -0.526 | 0.181 | 0.226 | 2.647 | -2.324 | 0.010 | 0.023 | 0.950 |
| rs6656401 | CR1 | PE(18:1p_18:1) | 0.257 | -0.477 | 0.144 | 0.205 | 1.784 | -2.323 | 0.079 | 0.023 | 0.950 |
| rs6733839 | BIN1 | TG(16:0_16:0_16:0) | -0.270 | 0.491 | 0.138 | 0.211 | -1.953 | 2.323 | 0.055 | 0.023 | 0.950 |
| rs1131497 | SORL1 | TG(17:0_18:2_22:6) | 0.348 | -0.506 | 0.141 | 0.218 | 2.462 | -2.322 | 0.016 | 0.023 | 0.950 |
| rs610932 | MS4A6A | TG(17:0_17:1_19:0) | -0.173 | 0.464 | 0.141 | 0.200 | -1.227 | 2.321 | 0.224 | 0.023 | 0.950 |
| rs6733839 | BIN1 | TG(16:1_20:1_22:4) | 0.141 | -0.467 | 0.132 | 0.201 | 1.071 | -2.321 | 0.288 | 0.023 | 0.950 |
| rs983392 | MS4A6A | SM(t42:1) | -0.241 | 0.478 | 0.139 | 0.206 | -1.735 | 2.321 | 0.087 | 0.023 | 0.950 |
| rs17125944 | FERMT2 | TG(53:4) | -0.427 | 0.469 | 0.168 | 0.202 | -2.538 | 2.321 | 0.013 | 0.023 | 0.950 |
| rs10498633 | SLC24A4 | TG(18:1_17:1_20:4) | 0.325 | -0.504 | 0.174 | 0.217 | 1.870 | -2.319 | 0.066 | 0.023 | 0.950 |
| rs983392 | MS4A6A | SM(d28:1) | -0.060 | 0.472 | 0.137 | 0.204 | -0.437 | 2.318 | 0.663 | 0.023 | 0.950 |
| rs3865444 | CD33 | TG(16:0_14:0_18:3) | -0.133 | 0.472 | 0.131 | 0.203 | -1.015 | 2.318 | 0.313 | 0.023 | 0.950 |
| rs190982 | MEF2C | TG(18:0_20:4_22:5) | -0.120 | 0.508 | 0.146 | 0.219 | -0.820 | 2.318 | 0.415 | 0.023 | 0.950 |
| rs610932 | MS4A6A | SM(t42:1) | -0.248 | 0.476 | 0.145 | 0.206 | -1.714 | 2.316 | 0.091 | 0.023 | 0.950 |
| rs190982 | MEF2C | TG(18:1_18:1_22:6) | -0.266 | 0.510 | 0.147 | 0.220 | -1.813 | 2.315 | 0.074 | 0.023 | 0.950 |
| rs9331896 | CLU | SM(d16:1_20:0) | -0.188 | 0.440 | 0.125 | 0.190 | -1.495 | 2.313 | 0.139 | 0.024 | 0.950 |
| rs6733839 | BIN1 | TG(18:1_18:2_22:0) | 0.163 | -0.464 | 0.131 | 0.201 | 1.238 | -2.311 | 0.220 | 0.024 | 0.950 |
| rs9331896 | CLU | Cer(d18:1_22:0) | -0.226 | 0.443 | 0.127 | 0.192 | -1.787 | 2.306 | 0.078 | 0.024 | 0.950 |
| rs28834970 | PTK2B | DG(16:0_16:0) | 0.365 | -0.506 | 0.155 | 0.219 | 2.351 | -2.304 | 0.021 | 0.024 | 0.950 |
| rs1476679 | ZCWPW1 | PC(32:1) | 0.287 | -0.462 | 0.132 | 0.201 | 2.171 | -2.298 | 0.033 | 0.024 | 0.950 |
| rs3752246 | ABCA7 | TG(18:1_18:1_22:1) | 0.281 | -0.465 | 0.141 | 0.203 | 1.997 | -2.295 | 0.050 | 0.025 | 0.950 |
| rs4147929 | ABCA7 | TG(18:1_18:1_22:1) | 0.281 | -0.465 | 0.141 | 0.203 | 1.997 | -2.295 | 0.050 | 0.025 | 0.950 |
| rs9349407 | CD2AP | TG(17:0_18:2_20:3) | 0.325 | -0.459 | 0.143 | 0.200 | 2.281 | -2.295 | 0.025 | 0.025 | 0.950 |
| rs10948363 | CD2AP | TG(17:0_18:2_20:3) | 0.325 | -0.459 | 0.143 | 0.200 | 2.281 | -2.295 | 0.025 | 0.025 | 0.950 |
| rs9331896 | CLU | TG(18:0_18:0_18:0) | 0.019 | -0.460 | 0.132 | 0.201 | 0.146 | -2.294 | 0.885 | 0.025 | 0.950 |
| rs11218343 | SORL1 | ChE(17:0) | -0.252 | 0.488 | 0.171 | 0.213 | -1.479 | 2.294 | 0.143 | 0.025 | 0.950 |
| rs1476679 | ZCWPW1 | SM(d40:2) | 0.233 | -0.490 | 0.140 | 0.214 | 1.664 | -2.294 | 0.100 | 0.025 | 0.950 |
| rs983392 | MS4A6A | Cer(d18:1_23:0) | -0.040 | 0.425 | 0.125 | 0.185 | -0.322 | 2.294 | 0.748 | 0.025 | 0.950 |
| rs7274581 | CASS4 | DG(22:4e) | 0.165 | -0.476 | 0.140 | 0.208 | 1.182 | -2.291 | 0.241 | 0.025 | 0.950 |
| rs10838725 | CELF1 | SM(t39:6) | -0.088 | 0.480 | 0.141 | 0.210 | -0.620 | 2.289 | 0.537 | 0.025 | 0.950 |
| rs9349407 | CD2AP | SM(d36:2) | 0.219 | -0.455 | 0.142 | 0.199 | 1.546 | -2.288 | 0.126 | 0.025 | 0.950 |
| rs10948363 | CD2AP | SM(d36:2) | 0.219 | -0.455 | 0.142 | 0.199 | 1.546 | -2.288 | 0.126 | 0.025 | 0.950 |
| rs1476679 | ZCWPW1 | Cer(m18:0_24:0) | 0.245 | -0.479 | 0.138 | 0.209 | 1.783 | -2.288 | 0.079 | 0.025 | 0.950 |
| rs1476679 | ZCWPW1 | TG(15:0_18:2_20:5) | 0.061 | -0.476 | 0.137 | 0.209 | 0.442 | -2.279 | 0.660 | 0.026 | 0.950 |
| rs6733839 | BIN1 | TG(16:0_18:1_20:3) | 0.115 | -0.430 | 0.124 | 0.189 | 0.927 | -2.276 | 0.357 | 0.026 | 0.950 |
| rs983392 | MS4A6A | TG(14:0e_18:0_20:1) | 0.175 | -0.483 | 0.143 | 0.212 | 1.219 | -2.275 | 0.227 | 0.026 | 0.950 |
| rs6656401 | CR1 | Cer(d18:0_24:0) | 0.130 | -0.464 | 0.143 | 0.204 | 0.907 | -2.274 | 0.367 | 0.026 | 0.950 |
| rs561655 | PICALM | Cer(d18:1_25:0) | -0.151 | 0.473 | 0.142 | 0.208 | -1.063 | 2.273 | 0.291 | 0.026 | 0.950 |
| rs3752246 | ABCA7 | TG(18:1_18:2_18:2) | 0.246 | -0.437 | 0.133 | 0.192 | 1.841 | -2.271 | 0.070 | 0.026 | 0.950 |
| rs4147929 | ABCA7 | TG(18:1_18:2_18:2) | 0.246 | -0.437 | 0.133 | 0.192 | 1.841 | -2.271 | 0.070 | 0.026 | 0.950 |
| rs983392 | MS4A6A | SM(d32:0) | -0.152 | 0.461 | 0.137 | 0.203 | -1.106 | 2.269 | 0.272 | 0.026 | 0.950 |
| rs11136000 | CLU | SM(d44:5) | -0.149 | 0.449 | 0.132 | 0.198 | -1.126 | 2.268 | 0.264 | 0.026 | 0.950 |
| rs1532278 | CLU | SM(d44:5) | -0.149 | 0.449 | 0.132 | 0.198 | -1.126 | 2.268 | 0.264 | 0.026 | 0.950 |
| rs561655 | PICALM | Cer(d18:2_25:0) | -0.030 | 0.440 | 0.132 | 0.194 | -0.228 | 2.266 | 0.820 | 0.026 | 0.950 |
| rs561655 | PICALM | TG(29:0_18:1_18:1) | -0.276 | 0.466 | 0.140 | 0.206 | -1.976 | 2.265 | 0.052 | 0.026 | 0.950 |
| rs12034383 | CR1 | SM(t36:1) | -0.234 | 0.485 | 0.151 | 0.214 | -1.547 | 2.263 | 0.126 | 0.027 | 0.950 |
| rs9331896 | CLU | SM(d38:2) | -0.148 | 0.465 | 0.136 | 0.205 | -1.090 | 2.262 | 0.279 | 0.027 | 0.950 |
| rs6733839 | BIN1 | TG(20:0_18:1_18:1) | 0.164 | -0.435 | 0.126 | 0.193 | 1.301 | -2.258 | 0.197 | 0.027 | 0.950 |
| rs7274581 | CASS4 | PC(18:0_22:6) | 0.140 | -0.482 | 0.144 | 0.214 | 0.974 | -2.256 | 0.333 | 0.027 | 0.950 |
| rs744373 | BIN1 | DG(18:1_18:1) | 0.173 | -0.440 | 0.134 | 0.195 | 1.290 | -2.256 | 0.201 | 0.027 | 0.950 |
| rs11136000 | CLU | SM(d37:1) | -0.228 | 0.471 | 0.140 | 0.209 | -1.633 | 2.255 | 0.107 | 0.027 | 0.950 |
| rs1532278 | CLU | SM(d37:1) | -0.228 | 0.471 | 0.140 | 0.209 | -1.633 | 2.255 | 0.107 | 0.027 | 0.950 |
| rs9331896 | CLU | SM(d33:1) | -0.170 | 0.472 | 0.138 | 0.209 | -1.230 | 2.255 | 0.222 | 0.027 | 0.950 |
| rs610932 | MS4A6A | TG(16:0_18:1_18:3) | -0.063 | 0.445 | 0.139 | 0.197 | -0.457 | 2.254 | 0.649 | 0.027 | 0.950 |
| rs744373 | BIN1 | TG(17:0_18:1_22:4) | 0.132 | -0.469 | 0.143 | 0.208 | 0.923 | -2.253 | 0.359 | 0.027 | 0.950 |
| rs983392 | MS4A6A | PC(18:0_20:3) | 0.045 | -0.465 | 0.139 | 0.206 | 0.320 | -2.253 | 0.750 | 0.027 | 0.950 |
| rs11136000 | CLU | SM(d39:2) | -0.151 | 0.466 | 0.138 | 0.207 | -1.093 | 2.253 | 0.278 | 0.027 | 0.950 |
| rs1532278 | CLU | SM(d39:2) | -0.151 | 0.466 | 0.138 | 0.207 | -1.093 | 2.253 | 0.278 | 0.027 | 0.950 |
| rs12034383 | CR1 | PI(18:0_18:1) | -0.360 | 0.461 | 0.144 | 0.205 | -2.498 | 2.252 | 0.015 | 0.027 | 0.950 |
| rs11136000 | CLU | SM(d39:1) | -0.157 | 0.444 | 0.132 | 0.197 | -1.191 | 2.251 | 0.237 | 0.027 | 0.950 |
| rs1532278 | CLU | SM(d39:1) | -0.157 | 0.444 | 0.132 | 0.197 | -1.191 | 2.251 | 0.237 | 0.027 | 0.950 |
| rs12034383 | CR1 | PC(30:2) | -0.333 | 0.476 | 0.149 | 0.211 | -2.234 | 2.249 | 0.028 | 0.027 | 0.950 |
| rs9331896 | CLU | SM(d40:1) | -0.121 | 0.454 | 0.133 | 0.202 | -0.906 | 2.249 | 0.368 | 0.027 | 0.950 |
| rs561655 | PICALM | ChE(22:6) | -0.033 | 0.419 | 0.127 | 0.186 | -0.259 | 2.248 | 0.797 | 0.028 | 0.950 |
| rs1131497 | SORL1 | TG(18:1_18:1_22:0) | -0.219 | 0.429 | 0.124 | 0.191 | -1.763 | 2.245 | 0.082 | 0.028 | 0.950 |
| rs561655 | PICALM | ChE(22:5) | -0.081 | 0.457 | 0.138 | 0.203 | -0.588 | 2.245 | 0.558 | 0.028 | 0.950 |
| rs2718058 | NME8 | TG(20:5_14:1_18:2) | 0.222 | -0.470 | 0.132 | 0.209 | 1.688 | -2.243 | 0.096 | 0.028 | 0.950 |
| rs2718058 | NME8 | SM(d42:2) | -0.189 | 0.448 | 0.126 | 0.200 | -1.507 | 2.243 | 0.136 | 0.028 | 0.950 |
| rs3752246 | ABCA7 | TG(16:1_17:1_18:2) | 0.129 | -0.435 | 0.135 | 0.194 | 0.956 | -2.242 | 0.342 | 0.028 | 0.950 |
| rs4147929 | ABCA7 | TG(16:1_17:1_18:2) | 0.129 | -0.435 | 0.135 | 0.194 | 0.956 | -2.242 | 0.342 | 0.028 | 0.950 |
| rs17125944 | FERMT2 | TG(20:1_18:1_22:6) | 0.153 | -0.503 | 0.187 | 0.224 | 0.817 | -2.242 | 0.416 | 0.028 | 0.950 |
| rs11767557 | EPHA1 | DG(32:2e) | -0.336 | 0.432 | 0.119 | 0.193 | -2.828 | 2.241 | 0.006 | 0.028 | 0.950 |
| rs10498633 | SLC24A4 | TG(26:0_18:1_18:1) | 0.433 | -0.505 | 0.181 | 0.226 | 2.396 | -2.240 | 0.019 | 0.028 | 0.950 |
| rs9349407 | CD2AP | SM(d33:1) | 0.230 | -0.466 | 0.149 | 0.208 | 1.544 | -2.239 | 0.127 | 0.028 | 0.950 |
| rs10948363 | CD2AP | SM(d33:1) | 0.230 | -0.466 | 0.149 | 0.208 | 1.544 | -2.239 | 0.127 | 0.028 | 0.950 |
| rs983392 | MS4A6A | TG(18:1_18:2_23:0) | 0.072 | -0.439 | 0.133 | 0.196 | 0.541 | -2.237 | 0.590 | 0.028 | 0.950 |
| rs983392 | MS4A6A | SM(d41:4) | 0.018 | 0.442 | 0.133 | 0.198 | 0.135 | 2.237 | 0.893 | 0.028 | 0.950 |
| rs983392 | MS4A6A | TG(16:0_18:1_18:3) | -0.044 | 0.442 | 0.134 | 0.198 | -0.327 | 2.236 | 0.745 | 0.028 | 0.950 |
| rs6733839 | BIN1 | TG(25:0_16:0_16:0) | -0.257 | 0.480 | 0.141 | 0.215 | -1.828 | 2.235 | 0.072 | 0.028 | 0.950 |
| rs11136000 | CLU | SM(d16:1_24:3) | -0.173 | 0.434 | 0.130 | 0.194 | -1.329 | 2.235 | 0.188 | 0.028 | 0.950 |
| rs1532278 | CLU | SM(d16:1_24:3) | -0.173 | 0.434 | 0.130 | 0.194 | -1.329 | 2.235 | 0.188 | 0.028 | 0.950 |
| rs10792832 | PICALM | Cer(d18:2_24:0) | -0.050 | 0.440 | 0.132 | 0.197 | -0.380 | 2.232 | 0.705 | 0.029 | 0.950 |
| rs3851179 | PICALM | Cer(d18:2_24:0) | -0.050 | 0.440 | 0.132 | 0.197 | -0.380 | 2.232 | 0.705 | 0.029 | 0.950 |
| rs9349407 | CD2AP | TG(17:0_18:1_22:5) | 0.178 | -0.416 | 0.133 | 0.186 | 1.338 | -2.230 | 0.185 | 0.029 | 0.950 |
| rs10948363 | CD2AP | TG(17:0_18:1_22:5) | 0.178 | -0.416 | 0.133 | 0.186 | 1.338 | -2.230 | 0.185 | 0.029 | 0.950 |
| rs12034383 | CR1 | PC(28:0) | -0.365 | 0.483 | 0.153 | 0.217 | -2.390 | 2.228 | 0.019 | 0.029 | 0.950 |
| rs6733839 | BIN1 | TG(18:1_18:2_22:5) | 0.153 | -0.454 | 0.133 | 0.204 | 1.145 | -2.228 | 0.256 | 0.029 | 0.950 |
| rs28834970 | PTK2B | SM(d37:1) | 0.081 | -0.467 | 0.149 | 0.210 | 0.548 | -2.226 | 0.585 | 0.029 | 0.950 |
| rs610932 | MS4A6A | TG(16:1_20:1_20:1) | 0.139 | -0.447 | 0.141 | 0.201 | 0.983 | -2.225 | 0.329 | 0.029 | 0.950 |
| rs983392 | MS4A6A | TG(16:1_18:2_18:3) | -0.069 | -0.445 | 0.135 | 0.200 | -0.507 | -2.224 | 0.613 | 0.029 | 0.950 |
| rs3818361 | CR1 | SM(d44:3) | 0.222 | -0.449 | 0.148 | 0.202 | 1.496 | -2.223 | 0.139 | 0.029 | 0.950 |
| rs6701713 | CR1 | SM(d44:3) | 0.222 | -0.449 | 0.148 | 0.202 | 1.496 | -2.223 | 0.139 | 0.029 | 0.950 |
| rs17125944 | FERMT2 | TG(26:1_18:1_18:2) | -0.427 | 0.505 | 0.189 | 0.227 | -2.260 | 2.222 | 0.027 | 0.029 | 0.950 |
| rs17125944 | FERMT2 | TG(18:1_22:5_22:6) | 0.270 | -0.519 | 0.194 | 0.234 | 1.390 | -2.219 | 0.169 | 0.030 | 0.950 |
| rs983392 | MS4A6A | PC(34:1) | -0.033 | 0.417 | 0.127 | 0.188 | -0.258 | 2.219 | 0.797 | 0.030 | 0.950 |
| rs9331896 | CLU | SM(d39:2) | -0.150 | 0.461 | 0.137 | 0.208 | -1.092 | 2.217 | 0.278 | 0.030 | 0.950 |
| rs11136000 | CLU | TG(16:0_17:1_18:1) | -0.144 | 0.437 | 0.132 | 0.198 | -1.089 | 2.211 | 0.280 | 0.030 | 0.950 |
| rs1532278 | CLU | TG(16:0_17:1_18:1) | -0.144 | 0.437 | 0.132 | 0.198 | -1.089 | 2.211 | 0.280 | 0.030 | 0.950 |
| rs3865444 | CD33 | TG(18:4_16:0_16:1) | -0.174 | 0.471 | 0.137 | 0.213 | -1.268 | 2.211 | 0.209 | 0.030 | 0.950 |
| rs1476679 | ZCWPW1 | DG() | -0.288 | 0.476 | 0.142 | 0.216 | -2.032 | 2.209 | 0.046 | 0.030 | 0.950 |
| rs983392 | MS4A6A | TG(15:0_16:0_18:1) | -0.055 | 0.467 | 0.143 | 0.211 | -0.388 | 2.209 | 0.699 | 0.030 | 0.950 |
| rs9331896 | CLU | PE(18:0_18:1) | -0.004 | -0.453 | 0.135 | 0.205 | -0.033 | -2.208 | 0.974 | 0.030 | 0.950 |
| rs9349407 | CD2AP | SM(d43:1) | 0.267 | -0.479 | 0.155 | 0.217 | 1.724 | -2.206 | 0.089 | 0.030 | 0.950 |
| rs10948363 | CD2AP | SM(d43:1) | 0.267 | -0.479 | 0.155 | 0.217 | 1.724 | -2.206 | 0.089 | 0.030 | 0.950 |
| rs744373 | BIN1 | TG(19:1_18:0_18:1) | 0.254 | -0.455 | 0.142 | 0.206 | 1.791 | -2.206 | 0.077 | 0.030 | 0.950 |
| rs983392 | MS4A6A | TG(18:1_18:2_24:1) | -0.001 | -0.435 | 0.133 | 0.197 | -0.007 | -2.205 | 0.994 | 0.031 | 0.950 |
| rs744373 | BIN1 | TG(17:0_18:1_20:3) | 0.105 | -0.377 | 0.117 | 0.171 | 0.898 | -2.205 | 0.372 | 0.031 | 0.950 |
| rs9331896 | CLU | SM(d16:1_24:3) | -0.171 | 0.430 | 0.129 | 0.195 | -1.327 | 2.204 | 0.189 | 0.031 | 0.950 |
| rs190982 | MEF2C | TG(20:3_18:2_18:2) | -0.319 | 0.459 | 0.139 | 0.209 | -2.296 | 2.202 | 0.025 | 0.031 | 0.950 |
| rs610932 | MS4A6A | TG(15:0_18:1_22:6) | -0.225 | 0.445 | 0.143 | 0.203 | -1.579 | 2.199 | 0.119 | 0.031 | 0.950 |
| rs6733839 | BIN1 | DG(36:5e) | 0.123 | -0.412 | 0.123 | 0.187 | 1.004 | -2.195 | 0.319 | 0.031 | 0.950 |
| rs7274581 | CASS4 | SM(d43:2) | -0.294 | 0.462 | 0.142 | 0.211 | -2.073 | 2.192 | 0.042 | 0.032 | 0.950 |
| rs3752246 | ABCA7 | TG(18:1_18:2_24:0) | 0.288 | -0.425 | 0.135 | 0.194 | 2.138 | -2.190 | 0.036 | 0.032 | 0.950 |
| rs4147929 | ABCA7 | TG(18:1_18:2_24:0) | 0.288 | -0.425 | 0.135 | 0.194 | 2.138 | -2.190 | 0.036 | 0.032 | 0.950 |
| rs2718058 | NME8 | TG(15:0_14:1_16:1) | 0.038 | -0.426 | 0.122 | 0.195 | 0.310 | -2.189 | 0.758 | 0.032 | 0.950 |
| rs610932 | MS4A6A | SM(d28:1) | -0.072 | 0.446 | 0.143 | 0.204 | -0.505 | 2.188 | 0.615 | 0.032 | 0.950 |
| rs6656401 | CR1 | Cer(d18:1_25:0) | 0.250 | -0.456 | 0.146 | 0.208 | 1.705 | -2.186 | 0.092 | 0.032 | 0.950 |
| rs10498633 | SLC24A4 | PC(18:1_13:0) | -0.455 | 0.477 | 0.175 | 0.219 | -2.598 | 2.185 | 0.011 | 0.032 | 0.950 |
| rs9331896 | CLU | SM(d28:1) | -0.284 | 0.456 | 0.138 | 0.209 | -2.062 | 2.185 | 0.043 | 0.032 | 0.950 |
| rs983392 | MS4A6A | TG(16:0_16:0_16:0) | -0.078 | 0.454 | 0.140 | 0.208 | -0.553 | 2.184 | 0.582 | 0.032 | 0.950 |
| rs12034383 | CR1 | PC(36:4) | -0.213 | 0.445 | 0.144 | 0.204 | -1.483 | 2.183 | 0.142 | 0.032 | 0.950 |
| rs3752246 | ABCA7 | DG(17:0_18:1) | 0.228 | -0.430 | 0.137 | 0.197 | 1.668 | -2.182 | 0.100 | 0.032 | 0.950 |
| rs4147929 | ABCA7 | DG(17:0_18:1) | 0.228 | -0.430 | 0.137 | 0.197 | 1.668 | -2.182 | 0.100 | 0.032 | 0.950 |
| rs11136000 | CLU | Cer(d18:1_23:0) | -0.201 | 0.415 | 0.127 | 0.190 | -1.577 | 2.182 | 0.119 | 0.032 | 0.950 |
| rs1532278 | CLU | Cer(d18:1_23:0) | -0.201 | 0.415 | 0.127 | 0.190 | -1.577 | 2.182 | 0.119 | 0.032 | 0.950 |
| rs9331896 | CLU | SM(d44:5) | -0.148 | 0.435 | 0.132 | 0.200 | -1.123 | 2.182 | 0.265 | 0.032 | 0.950 |
| rs10792832 | PICALM | SM(d18:2_16:0) | -0.014 | 0.414 | 0.127 | 0.190 | -0.112 | 2.181 | 0.911 | 0.032 | 0.950 |
| rs3851179 | PICALM | SM(d18:2_16:0) | -0.014 | 0.414 | 0.127 | 0.190 | -0.112 | 2.181 | 0.911 | 0.032 | 0.950 |
| rs1476679 | ZCWPW1 | DG(18:0_18:1) | -0.421 | 0.444 | 0.134 | 0.204 | -3.150 | 2.181 | 0.002 | 0.032 | 0.950 |
| rs17125944 | FERMT2 | PE(18:1p_18:1) | -0.186 | 0.491 | 0.187 | 0.225 | -0.995 | 2.181 | 0.323 | 0.032 | 0.950 |
| rs28834970 | PTK2B | DG(18:0_18:1) | 0.290 | -0.464 | 0.151 | 0.213 | 1.921 | -2.181 | 0.059 | 0.032 | 0.950 |
| rs610932 | MS4A6A | TG(15:0_16:0_16:0) | -0.109 | 0.473 | 0.153 | 0.217 | -0.714 | 2.180 | 0.478 | 0.032 | 0.950 |
| rs3818361 | CR1 | PC(37:3) | 0.177 | -0.474 | 0.160 | 0.218 | 1.108 | -2.177 | 0.272 | 0.033 | 0.950 |
| rs6701713 | CR1 | PC(37:3) | 0.177 | -0.474 | 0.160 | 0.218 | 1.108 | -2.177 | 0.272 | 0.033 | 0.950 |
| rs744373 | BIN1 | TG(60:6) | 0.204 | -0.451 | 0.143 | 0.207 | 1.433 | -2.176 | 0.156 | 0.033 | 0.950 |
| rs11136000 | CLU | TG(18:1_22:5_22:6) | 0.313 | -0.464 | 0.143 | 0.213 | 2.196 | -2.175 | 0.031 | 0.033 | 0.950 |
| rs1532278 | CLU | TG(18:1_22:5_22:6) | 0.313 | -0.464 | 0.143 | 0.213 | 2.196 | -2.175 | 0.031 | 0.033 | 0.950 |
| rs1131497 | SORL1 | PC(16:0_20:5) | 0.036 | -0.473 | 0.141 | 0.218 | 0.257 | -2.174 | 0.798 | 0.033 | 0.950 |
| rs983392 | MS4A6A | PE(16:0p_22:6) | 0.020 | 0.443 | 0.138 | 0.204 | 0.145 | 2.174 | 0.885 | 0.033 | 0.950 |
| rs17125944 | FERMT2 | TG(19:0_18:1_18:1) | -0.309 | 0.436 | 0.167 | 0.201 | -1.847 | 2.173 | 0.069 | 0.033 | 0.950 |
| rs983392 | MS4A6A | DG(18:1_18:1) | 0.077 | -0.419 | 0.130 | 0.193 | 0.590 | -2.172 | 0.557 | 0.033 | 0.950 |
| rs561655 | PICALM | TG(18:1_18:1_21:1) | -0.311 | 0.447 | 0.140 | 0.206 | -2.215 | 2.171 | 0.030 | 0.033 | 0.950 |
| rs3752246 | ABCA7 | TG(26:0_18:1_18:2) | 0.307 | -0.434 | 0.138 | 0.200 | 2.216 | -2.171 | 0.030 | 0.033 | 0.950 |
| rs4147929 | ABCA7 | TG(26:0_18:1_18:2) | 0.307 | -0.434 | 0.138 | 0.200 | 2.216 | -2.171 | 0.030 | 0.033 | 0.950 |
| rs744373 | BIN1 | TG(15:0_16:0_18:3) | -0.157 | 0.465 | 0.147 | 0.214 | -1.062 | 2.168 | 0.292 | 0.033 | 0.950 |
| rs744373 | BIN1 | PI(16:0_18:1) | -0.412 | 0.462 | 0.147 | 0.213 | -2.813 | 2.167 | 0.006 | 0.033 | 0.950 |
| rs983392 | MS4A6A | Cer(m18:1_20:0) | 0.038 | 0.397 | 0.124 | 0.183 | 0.308 | 2.167 | 0.759 | 0.033 | 0.950 |
| rs610932 | MS4A6A | TG(33:4e) | 0.322 | -0.449 | 0.146 | 0.207 | 2.206 | -2.165 | 0.030 | 0.034 | 0.950 |
| rs6733839 | BIN1 | TG(15:0_16:0_16:1) | -0.128 | 0.477 | 0.144 | 0.220 | -0.885 | 2.165 | 0.379 | 0.034 | 0.950 |
| rs11136000 | CLU | PE(16:0p_22:6) | -0.273 | 0.456 | 0.141 | 0.211 | -1.940 | 2.164 | 0.056 | 0.034 | 0.950 |
| rs1532278 | CLU | PE(16:0p_22:6) | -0.273 | 0.456 | 0.141 | 0.211 | -1.940 | 2.164 | 0.056 | 0.034 | 0.950 |
| rs3865444 | CD33 | PC(16:0_20:5) | 0.054 | -0.474 | 0.141 | 0.219 | 0.381 | -2.164 | 0.704 | 0.034 | 0.950 |
| rs744373 | BIN1 | TG(56:7) | 0.144 | -0.442 | 0.140 | 0.204 | 1.028 | -2.164 | 0.307 | 0.034 | 0.950 |
| rs11218343 | SORL1 | TG(15:0_16:0_18:3) | -0.356 | 0.486 | 0.180 | 0.224 | -1.974 | 2.163 | 0.052 | 0.034 | 0.950 |
| rs9349407 | CD2AP | SM(d35:2) | 0.255 | -0.440 | 0.145 | 0.203 | 1.755 | -2.163 | 0.083 | 0.034 | 0.950 |
| rs10948363 | CD2AP | SM(d35:2) | 0.255 | -0.440 | 0.145 | 0.203 | 1.755 | -2.163 | 0.083 | 0.034 | 0.950 |
| rs2718058 | NME8 | SM(d41:0) | -0.103 | 0.453 | 0.132 | 0.210 | -0.778 | 2.161 | 0.439 | 0.034 | 0.950 |
| rs983392 | MS4A6A | PC(32:1) | -0.124 | 0.433 | 0.135 | 0.201 | -0.916 | 2.160 | 0.363 | 0.034 | 0.950 |
| rs983392 | MS4A6A | TG(16:0_18:1_20:3) | 0.157 | -0.405 | 0.127 | 0.188 | 1.243 | -2.159 | 0.218 | 0.034 | 0.950 |
| rs6656401 | CR1 | PI(18:1_20:4) | 0.130 | -0.430 | 0.140 | 0.199 | 0.926 | -2.159 | 0.357 | 0.034 | 0.950 |
| rs3865444 | CD33 | SM(d37:1) | 0.194 | -0.455 | 0.136 | 0.211 | 1.433 | -2.157 | 0.156 | 0.034 | 0.950 |
| rs7274581 | CASS4 | SM(t42:1) | -0.322 | 0.448 | 0.140 | 0.208 | -2.302 | 2.157 | 0.024 | 0.034 | 0.950 |
| rs11136000 | CLU | SM(t36:1) | -0.177 | 0.465 | 0.144 | 0.216 | -1.228 | 2.156 | 0.223 | 0.034 | 0.950 |
| rs1532278 | CLU | SM(t36:1) | -0.177 | 0.465 | 0.144 | 0.216 | -1.228 | 2.156 | 0.223 | 0.034 | 0.950 |
| rs7561528 | BIN1 | SM(d32:4) | 0.352 | -0.432 | 0.143 | 0.200 | 2.458 | -2.156 | 0.016 | 0.034 | 0.950 |
| rs10498633 | SLC24A4 | TG(19:1_18:1_18:2) | 0.242 | -0.461 | 0.171 | 0.214 | 1.413 | -2.154 | 0.162 | 0.034 | 0.950 |
| rs3818361 | CR1 | Cer(d18:1_25:0) | 0.262 | -0.452 | 0.154 | 0.210 | 1.703 | -2.154 | 0.093 | 0.034 | 0.950 |
| rs6701713 | CR1 | Cer(d18:1_25:0) | 0.262 | -0.452 | 0.154 | 0.210 | 1.703 | -2.154 | 0.093 | 0.034 | 0.950 |
| rs3752246 | ABCA7 | TG(18:3_14:1_18:2) | 0.412 | -0.432 | 0.139 | 0.201 | 2.960 | -2.153 | 0.004 | 0.035 | 0.950 |
| rs4147929 | ABCA7 | TG(18:3_14:1_18:2) | 0.412 | -0.432 | 0.139 | 0.201 | 2.960 | -2.153 | 0.004 | 0.035 | 0.950 |
| rs11218343 | SORL1 | PC(44:5) | -0.278 | 0.469 | 0.175 | 0.218 | -1.588 | 2.150 | 0.117 | 0.035 | 0.950 |
| rs3865444 | CD33 | PC(16:0_20:3) | 0.074 | -0.463 | 0.138 | 0.215 | 0.537 | -2.150 | 0.593 | 0.035 | 0.950 |
| rs10498633 | SLC24A4 | PC(20:0_18:1) | -0.301 | 0.471 | 0.176 | 0.219 | -1.716 | 2.148 | 0.090 | 0.035 | 0.950 |
| rs983392 | MS4A6A | Cer(m18:0_22:0) | 0.073 | 0.405 | 0.127 | 0.189 | 0.574 | 2.147 | 0.567 | 0.035 | 0.950 |
| rs7274581 | CASS4 | SM(d40:1) | -0.173 | 0.436 | 0.137 | 0.203 | -1.264 | 2.147 | 0.210 | 0.035 | 0.950 |
| rs9349407 | CD2AP | SM(d37:1) | 0.200 | -0.449 | 0.149 | 0.209 | 1.341 | -2.146 | 0.184 | 0.035 | 0.950 |
| rs10948363 | CD2AP | SM(d37:1) | 0.200 | -0.449 | 0.149 | 0.209 | 1.341 | -2.146 | 0.184 | 0.035 | 0.950 |
| rs1476679 | ZCWPW1 | PC(20:0_18:1) | 0.304 | -0.448 | 0.137 | 0.209 | 2.212 | -2.144 | 0.030 | 0.035 | 0.950 |
| rs9349407 | CD2AP | SM(t34:0) | 0.255 | -0.453 | 0.151 | 0.211 | 1.694 | -2.144 | 0.094 | 0.035 | 0.950 |
| rs10948363 | CD2AP | SM(t34:0) | 0.255 | -0.453 | 0.151 | 0.211 | 1.694 | -2.144 | 0.094 | 0.035 | 0.950 |
| rs17125944 | FERMT2 | TG(18:0_16:0_21:0) | -0.317 | 0.485 | 0.188 | 0.226 | -1.682 | 2.144 | 0.097 | 0.035 | 0.950 |
| rs9331896 | CLU | SM(d39:1) | -0.156 | 0.427 | 0.131 | 0.199 | -1.187 | 2.143 | 0.239 | 0.035 | 0.950 |
| rs6656401 | CR1 | SM(d18:1_24:0) | 0.107 | -0.446 | 0.146 | 0.208 | 0.732 | -2.142 | 0.466 | 0.035 | 0.950 |
| rs3865444 | CD33 | DG(16:1_18:2) | 0.061 | -0.419 | 0.126 | 0.196 | 0.485 | -2.138 | 0.629 | 0.036 | 0.950 |
| rs11136000 | CLU | TG(15:0_18:1_20:4) | -0.205 | 0.393 | 0.123 | 0.184 | -1.667 | 2.138 | 0.100 | 0.036 | 0.950 |
| rs1532278 | CLU | TG(15:0_18:1_20:4) | -0.205 | 0.393 | 0.123 | 0.184 | -1.667 | 2.138 | 0.100 | 0.036 | 0.950 |
| rs10792832 | PICALM | TG(18:0_16:0_19:0) | -0.315 | 0.431 | 0.135 | 0.202 | -2.330 | 2.137 | 0.023 | 0.036 | 0.950 |
| rs3851179 | PICALM | TG(18:0_16:0_19:0) | -0.315 | 0.431 | 0.135 | 0.202 | -2.330 | 2.137 | 0.023 | 0.036 | 0.950 |
| rs3752246 | ABCA7 | TG(16:0e_18:0_20:1) | 0.223 | -0.464 | 0.150 | 0.217 | 1.481 | -2.137 | 0.143 | 0.036 | 0.950 |
| rs4147929 | ABCA7 | TG(16:0e_18:0_20:1) | 0.223 | -0.464 | 0.150 | 0.217 | 1.481 | -2.137 | 0.143 | 0.036 | 0.950 |
| rs28834970 | PTK2B | SM(d44:5) | 0.231 | -0.432 | 0.143 | 0.202 | 1.611 | -2.136 | 0.111 | 0.036 | 0.950 |
| rs11136000 | CLU | PC(33:2) | -0.203 | 0.453 | 0.142 | 0.212 | -1.431 | 2.134 | 0.157 | 0.036 | 0.950 |
| rs1532278 | CLU | PC(33:2) | -0.203 | 0.453 | 0.142 | 0.212 | -1.431 | 2.134 | 0.157 | 0.036 | 0.950 |
| rs1131497 | SORL1 | TG(17:0_18:1_20:5) | -0.305 | 0.396 | 0.120 | 0.185 | -2.531 | 2.134 | 0.013 | 0.036 | 0.950 |
| rs7561528 | BIN1 | TG(14:0_18:3_18:3) | -0.012 | -0.421 | 0.141 | 0.198 | -0.085 | -2.133 | 0.933 | 0.036 | 0.950 |
| rs6733839 | BIN1 | TG(17:0_17:1_17:1) | -0.074 | 0.449 | 0.138 | 0.211 | -0.537 | 2.133 | 0.593 | 0.036 | 0.950 |
| rs7561528 | BIN1 | DG(18:1_18:1) | 0.099 | -0.413 | 0.138 | 0.194 | 0.715 | -2.132 | 0.477 | 0.036 | 0.950 |
| rs610932 | MS4A6A | PC(31:0) | -0.197 | 0.449 | 0.148 | 0.211 | -1.326 | 2.132 | 0.189 | 0.036 | 0.950 |
| rs983392 | MS4A6A | TG(18:1_18:2_23:1) | 0.081 | -0.434 | 0.138 | 0.204 | 0.590 | -2.130 | 0.557 | 0.036 | 0.950 |
| rs983392 | MS4A6A | TG(53:4) | 0.061 | -0.391 | 0.124 | 0.184 | 0.493 | -2.130 | 0.624 | 0.037 | 0.950 |
| rs3752246 | ABCA7 | DG(36:5e) | 0.166 | -0.394 | 0.128 | 0.185 | 1.294 | -2.129 | 0.200 | 0.037 | 0.950 |
| rs4147929 | ABCA7 | DG(36:5e) | 0.166 | -0.394 | 0.128 | 0.185 | 1.294 | -2.129 | 0.200 | 0.037 | 0.950 |
| rs3752246 | ABCA7 | TG(14:0_18:2_20:5) | 0.186 | -0.410 | 0.133 | 0.193 | 1.397 | -2.129 | 0.167 | 0.037 | 0.950 |
| rs4147929 | ABCA7 | TG(14:0_18:2_20:5) | 0.186 | -0.410 | 0.133 | 0.193 | 1.397 | -2.129 | 0.167 | 0.037 | 0.950 |
| rs1131497 | SORL1 | TG(20:0_18:1_18:1) | -0.245 | 0.409 | 0.125 | 0.192 | -1.965 | 2.129 | 0.053 | 0.037 | 0.950 |
| rs11136000 | CLU | SM(d18:1_24:3) | -0.220 | 0.410 | 0.129 | 0.193 | -1.706 | 2.126 | 0.092 | 0.037 | 0.950 |
| rs1532278 | CLU | SM(d18:1_24:3) | -0.220 | 0.410 | 0.129 | 0.193 | -1.706 | 2.126 | 0.092 | 0.037 | 0.950 |
| rs9331896 | CLU | PE(16:0p_22:6) | -0.271 | 0.450 | 0.140 | 0.212 | -1.939 | 2.125 | 0.056 | 0.037 | 0.950 |
| rs35349669 | INPP5D | SM(d30:1) | 0.054 | -0.428 | 0.144 | 0.201 | 0.377 | -2.124 | 0.708 | 0.037 | 0.950 |
| rs6656401 | CR1 | Cer(m18:1_22:0) | 0.208 | -0.432 | 0.143 | 0.203 | 1.455 | -2.124 | 0.150 | 0.037 | 0.950 |
| rs744373 | BIN1 | TG(15:0_16:0_16:1) | -0.116 | 0.462 | 0.150 | 0.218 | -0.778 | 2.123 | 0.439 | 0.037 | 0.950 |
| rs6656401 | CR1 | Cer(d18:0_18:0) | 0.198 | -0.436 | 0.144 | 0.205 | 1.370 | -2.122 | 0.175 | 0.037 | 0.950 |
| rs28834970 | PTK2B | SM(d40:1) | 0.249 | -0.436 | 0.146 | 0.205 | 1.715 | -2.122 | 0.091 | 0.037 | 0.950 |
| rs9331896 | CLU | SM(t36:1) | -0.176 | 0.460 | 0.143 | 0.217 | -1.226 | 2.122 | 0.224 | 0.037 | 0.950 |
| rs9349407 | CD2AP | PC(36:3) | 0.038 | 0.424 | 0.142 | 0.200 | 0.268 | 2.122 | 0.790 | 0.037 | 0.950 |
| rs10948363 | CD2AP | PC(36:3) | 0.038 | 0.424 | 0.142 | 0.200 | 0.268 | 2.122 | 0.790 | 0.037 | 0.950 |
| rs6733839 | BIN1 | TG(14:0_14:3_18:2) | -0.181 | 0.449 | 0.139 | 0.212 | -1.303 | 2.122 | 0.197 | 0.037 | 0.950 |
| rs744373 | BIN1 | TG(19:1_18:1_20:4) | 0.183 | -0.455 | 0.148 | 0.215 | 1.237 | -2.122 | 0.220 | 0.037 | 0.950 |
| rs983392 | MS4A6A | Cer(d18:1_26:1) | -0.062 | 0.422 | 0.134 | 0.199 | -0.459 | 2.121 | 0.648 | 0.037 | 0.950 |
| rs17125944 | FERMT2 | TG(16:0_18:1_22:6) | 0.187 | -0.487 | 0.191 | 0.230 | 0.978 | -2.120 | 0.331 | 0.037 | 0.950 |
| rs610932 | MS4A6A | ChE(18:3) | -0.067 | 0.392 | 0.130 | 0.185 | -0.518 | 2.118 | 0.606 | 0.037 | 0.950 |
| rs744373 | BIN1 | PC(37:4) | 0.304 | -0.453 | 0.147 | 0.214 | 2.066 | -2.117 | 0.042 | 0.038 | 0.950 |
| rs3865444 | CD33 | PC(36:4) | 0.219 | -0.436 | 0.133 | 0.206 | 1.654 | -2.116 | 0.102 | 0.038 | 0.950 |
| rs983392 | MS4A6A | Cer(m18:1_24:1) | -0.001 | 0.406 | 0.130 | 0.192 | -0.010 | 2.115 | 0.992 | 0.038 | 0.950 |
| rs7561528 | BIN1 | TG(19:1_18:0_18:1) | 0.199 | -0.436 | 0.148 | 0.206 | 1.351 | -2.114 | 0.181 | 0.038 | 0.950 |
| rs28834970 | PTK2B | PI(16:0_20:3) | -0.187 | 0.429 | 0.144 | 0.203 | -1.297 | 2.110 | 0.199 | 0.038 | 0.950 |
| rs11136000 | CLU | TG(16:0_14:0_18:3) | 0.026 | -0.423 | 0.134 | 0.200 | 0.192 | -2.109 | 0.848 | 0.038 | 0.950 |
| rs1532278 | CLU | TG(16:0_14:0_18:3) | 0.026 | -0.423 | 0.134 | 0.200 | 0.192 | -2.109 | 0.848 | 0.038 | 0.950 |
| rs3865444 | CD33 | PC(18:2_20:4) | 0.056 | -0.454 | 0.139 | 0.215 | 0.404 | -2.107 | 0.687 | 0.038 | 0.950 |
| rs10498633 | SLC24A4 | ChE(20:2) | -0.267 | 0.461 | 0.175 | 0.219 | -1.524 | 2.107 | 0.132 | 0.038 | 0.950 |
| rs10838725 | CELF1 | Cer(d16:1_16:0) | -0.182 | 0.450 | 0.144 | 0.214 | -1.267 | 2.107 | 0.209 | 0.039 | 0.950 |
| rs11767557 | EPHA1 | TG(16:1_17:1_18:1) | 0.299 | -0.402 | 0.118 | 0.191 | 2.540 | -2.107 | 0.013 | 0.039 | 0.950 |
| rs10498633 | SLC24A4 | TG(29:0_18:1_18:1) | 0.263 | -0.457 | 0.174 | 0.217 | 1.513 | -2.106 | 0.135 | 0.039 | 0.950 |
| rs9349407 | CD2AP | SM(d18:1_24:3) | 0.222 | -0.406 | 0.138 | 0.193 | 1.613 | -2.106 | 0.111 | 0.039 | 0.950 |
| rs10948363 | CD2AP | SM(d18:1_24:3) | 0.222 | -0.406 | 0.138 | 0.193 | 1.613 | -2.106 | 0.111 | 0.039 | 0.950 |
| rs190982 | MEF2C | TG(18:1_20:4_22:5) | -0.261 | 0.453 | 0.143 | 0.215 | -1.821 | 2.105 | 0.073 | 0.039 | 0.950 |
| rs610932 | MS4A6A | TG(16:0_14:0_14:0) | -0.158 | 0.450 | 0.150 | 0.214 | -1.048 | 2.104 | 0.298 | 0.039 | 0.950 |
| rs17125944 | FERMT2 | PC(38:2) | 0.153 | -0.490 | 0.194 | 0.233 | 0.790 | -2.104 | 0.432 | 0.039 | 0.950 |
| rs1476679 | ZCWPW1 | Cer(m18:1_22:0) | 0.165 | -0.433 | 0.135 | 0.206 | 1.224 | -2.104 | 0.225 | 0.039 | 0.950 |
| rs1131497 | SORL1 | TG(20:0_18:1_20:4) | -0.160 | 0.451 | 0.139 | 0.214 | -1.148 | 2.103 | 0.255 | 0.039 | 0.950 |
| rs1131497 | SORL1 | PC(38:8) | 0.171 | -0.443 | 0.137 | 0.211 | 1.247 | -2.102 | 0.216 | 0.039 | 0.950 |
| rs9331896 | CLU | TG(16:0_17:1_18:1) | -0.143 | 0.419 | 0.132 | 0.199 | -1.085 | 2.102 | 0.281 | 0.039 | 0.950 |
| rs17125944 | FERMT2 | TG(18:1_20:4_22:5) | 0.268 | -0.492 | 0.195 | 0.234 | 1.376 | -2.102 | 0.173 | 0.039 | 0.950 |
| rs9331896 | CLU | TG(18:0_16:1_24:0) | -0.014 | -0.432 | 0.136 | 0.206 | -0.106 | -2.101 | 0.916 | 0.039 | 0.950 |
| rs11136000 | CLU | SM(d37:2) | -0.159 | 0.429 | 0.137 | 0.204 | -1.166 | 2.097 | 0.247 | 0.039 | 0.950 |
| rs1532278 | CLU | SM(d37:2) | -0.159 | 0.429 | 0.137 | 0.204 | -1.166 | 2.097 | 0.247 | 0.039 | 0.950 |
| rs3752246 | ABCA7 | DG(18:1_22:5) | 0.283 | -0.429 | 0.142 | 0.205 | 1.998 | -2.097 | 0.049 | 0.039 | 0.950 |
| rs4147929 | ABCA7 | DG(18:1_22:5) | 0.283 | -0.429 | 0.142 | 0.205 | 1.998 | -2.097 | 0.049 | 0.039 | 0.950 |
| rs11136000 | CLU | TG(18:4_16:0_20:4) | 0.128 | -0.445 | 0.142 | 0.212 | 0.901 | -2.097 | 0.370 | 0.039 | 0.950 |
| rs1532278 | CLU | TG(18:4_16:0_20:4) | 0.128 | -0.445 | 0.142 | 0.212 | 0.901 | -2.097 | 0.370 | 0.039 | 0.950 |
| rs983392 | MS4A6A | TG(28:0_18:1_18:1) | 0.072 | -0.438 | 0.141 | 0.209 | 0.512 | -2.096 | 0.610 | 0.040 | 0.950 |
| rs11136000 | CLU | TG(18:0_16:1_24:0) | -0.014 | -0.429 | 0.137 | 0.205 | -0.106 | -2.095 | 0.916 | 0.040 | 0.950 |
| rs1532278 | CLU | TG(18:0_16:1_24:0) | -0.014 | -0.429 | 0.137 | 0.205 | -0.106 | -2.095 | 0.916 | 0.040 | 0.950 |
| rs9331896 | CLU | SM(d34:5) | -0.129 | 0.458 | 0.144 | 0.219 | -0.897 | 2.095 | 0.373 | 0.040 | 0.950 |
| rs3752246 | ABCA7 | TG(26:1_18:1_18:2) | 0.319 | -0.430 | 0.142 | 0.205 | 2.242 | -2.094 | 0.028 | 0.040 | 0.950 |
| rs4147929 | ABCA7 | TG(26:1_18:1_18:2) | 0.319 | -0.430 | 0.142 | 0.205 | 2.242 | -2.094 | 0.028 | 0.040 | 0.950 |
| rs9331896 | CLU | TG(15:0_18:1_20:4) | -0.203 | 0.387 | 0.122 | 0.185 | -1.665 | 2.094 | 0.100 | 0.040 | 0.950 |
| rs610932 | MS4A6A | SM(t18:0_24:2) | -0.277 | 0.438 | 0.147 | 0.209 | -1.881 | 2.093 | 0.064 | 0.040 | 0.950 |
| rs11136000 | CLU | SM(d17:1_18:3) | -0.185 | 0.415 | 0.133 | 0.199 | -1.393 | 2.092 | 0.168 | 0.040 | 0.950 |
| rs1532278 | CLU | SM(d17:1_18:3) | -0.185 | 0.415 | 0.133 | 0.199 | -1.393 | 2.092 | 0.168 | 0.040 | 0.950 |
| rs7274581 | CASS4 | TG(18:1_18:1_22:5) | 0.157 | -0.422 | 0.136 | 0.202 | 1.159 | -2.092 | 0.250 | 0.040 | 0.950 |
| rs610932 | MS4A6A | Cer(m18:0_24:1) | 0.030 | 0.406 | 0.136 | 0.194 | 0.218 | 2.091 | 0.828 | 0.040 | 0.950 |
| rs9331896 | CLU | TG(18:0_18:0_20:0) | 0.013 | -0.423 | 0.133 | 0.202 | 0.100 | -2.091 | 0.921 | 0.040 | 0.950 |
| rs9331896 | CLU | SM(d18:1_24:3) | -0.218 | 0.405 | 0.128 | 0.194 | -1.705 | 2.089 | 0.092 | 0.040 | 0.950 |
| rs744373 | BIN1 | PC(36:3) | 0.043 | -0.427 | 0.141 | 0.205 | 0.309 | -2.089 | 0.758 | 0.040 | 0.950 |
| rs1476679 | ZCWPW1 | Cer(d18:1_24:0) | 0.200 | -0.417 | 0.131 | 0.200 | 1.525 | -2.088 | 0.131 | 0.040 | 0.950 |
| rs1476679 | ZCWPW1 | SM(d17:1_13:0) | 0.137 | -0.452 | 0.142 | 0.216 | 0.966 | -2.088 | 0.337 | 0.040 | 0.950 |
| rs10498633 | SLC24A4 | TG(28:0_16:0_18:1) | 0.460 | -0.473 | 0.182 | 0.227 | 2.532 | -2.086 | 0.013 | 0.040 | 0.950 |
| rs10792832 | PICALM | SM(d36:5) | -0.011 | 0.392 | 0.126 | 0.188 | -0.089 | 2.086 | 0.929 | 0.040 | 0.950 |
| rs3851179 | PICALM | SM(d36:5) | -0.011 | 0.392 | 0.126 | 0.188 | -0.089 | 2.086 | 0.929 | 0.040 | 0.950 |
| rs11136000 | CLU | TG(16:1_20:1_22:4) | 0.188 | -0.420 | 0.135 | 0.201 | 1.394 | -2.084 | 0.167 | 0.041 | 0.950 |
| rs1532278 | CLU | TG(16:1_20:1_22:4) | 0.188 | -0.420 | 0.135 | 0.201 | 1.394 | -2.084 | 0.167 | 0.041 | 0.950 |
| rs28834970 | PTK2B | PC(32:0) | 0.342 | -0.454 | 0.154 | 0.218 | 2.211 | -2.084 | 0.030 | 0.041 | 0.950 |
| rs744373 | BIN1 | TG(18:1_18:1_22:0) | 0.181 | -0.397 | 0.131 | 0.191 | 1.383 | -2.082 | 0.171 | 0.041 | 0.950 |
| rs744373 | BIN1 | TG(18:1_18:2_24:1) | 0.192 | -0.425 | 0.140 | 0.204 | 1.369 | -2.081 | 0.175 | 0.041 | 0.950 |
| rs6656401 | CR1 | Cer(d18:0_22:0) | 0.185 | -0.428 | 0.145 | 0.206 | 1.279 | -2.079 | 0.205 | 0.041 | 0.950 |
| rs10498633 | SLC24A4 | Cer(d18:0_18:0) | -0.077 | 0.438 | 0.169 | 0.211 | -0.456 | 2.077 | 0.649 | 0.041 | 0.950 |
| rs11136000 | CLU | SM(d38:1) | -0.180 | 0.398 | 0.128 | 0.192 | -1.404 | 2.075 | 0.164 | 0.041 | 0.950 |
| rs1532278 | CLU | SM(d38:1) | -0.180 | 0.398 | 0.128 | 0.192 | -1.404 | 2.075 | 0.164 | 0.041 | 0.950 |
| rs561655 | PICALM | Cer(d38:1) | -0.232 | 0.409 | 0.134 | 0.197 | -1.727 | 2.074 | 0.088 | 0.042 | 0.950 |
| rs983392 | MS4A6A | DG(18:0_18:0) | 0.182 | -0.411 | 0.134 | 0.198 | 1.361 | -2.074 | 0.178 | 0.042 | 0.950 |
| rs17125944 | FERMT2 | Cer(d16:0_24:1) | -0.306 | 0.488 | 0.196 | 0.235 | -1.564 | 2.074 | 0.122 | 0.042 | 0.950 |
| rs2718058 | NME8 | SM(t18:0_24:2) | -0.313 | 0.441 | 0.134 | 0.213 | -2.337 | 2.073 | 0.022 | 0.042 | 0.950 |
| rs983392 | MS4A6A | SM(d16:1_20:0) | -0.064 | 0.388 | 0.126 | 0.187 | -0.506 | 2.072 | 0.614 | 0.042 | 0.950 |
| rs1476679 | ZCWPW1 | SM(d18:1_21:0) | 0.074 | -0.418 | 0.133 | 0.202 | 0.559 | -2.072 | 0.578 | 0.042 | 0.950 |
| rs7274581 | CASS4 | DG(36:1) | 0.190 | -0.392 | 0.127 | 0.189 | 1.493 | -2.072 | 0.140 | 0.042 | 0.950 |
| rs11218343 | SORL1 | TG(33:4e) | -0.456 | 0.448 | 0.174 | 0.217 | -2.627 | 2.071 | 0.010 | 0.042 | 0.950 |
| rs10498633 | SLC24A4 | TG(20:0_18:2_22:6) | 0.399 | -0.460 | 0.178 | 0.222 | 2.241 | -2.070 | 0.028 | 0.042 | 0.950 |
| rs983392 | MS4A6A | PC(42:7) | -0.271 | 0.434 | 0.141 | 0.209 | -1.914 | 2.070 | 0.060 | 0.042 | 0.950 |
| rs10498633 | SLC24A4 | Cer(d18:0_22:0) | -0.143 | 0.444 | 0.172 | 0.214 | -0.832 | 2.070 | 0.408 | 0.042 | 0.950 |
| rs10838725 | CELF1 | PC(18:1_20:3) | 0.223 | -0.445 | 0.145 | 0.215 | 1.539 | -2.069 | 0.128 | 0.042 | 0.950 |
| rs610932 | MS4A6A | TG(18:1_18:1_22:6) | -0.438 | 0.440 | 0.150 | 0.213 | -2.926 | 2.069 | 0.005 | 0.042 | 0.950 |
| rs610932 | MS4A6A | PC(34:1) | -0.002 | 0.385 | 0.131 | 0.186 | -0.014 | 2.067 | 0.989 | 0.042 | 0.950 |
| rs28834970 | PTK2B | ChE(20:2) | 0.034 | -0.433 | 0.148 | 0.209 | 0.230 | -2.067 | 0.818 | 0.042 | 0.950 |
| rs983392 | MS4A6A | TG(16:0_14:0_14:0) | -0.170 | 0.445 | 0.145 | 0.215 | -1.168 | 2.067 | 0.246 | 0.042 | 0.950 |
| rs11136000 | CLU | TG(18:1_17:1_18:3) | 0.137 | -0.431 | 0.139 | 0.208 | 0.982 | -2.066 | 0.329 | 0.042 | 0.950 |
| rs1532278 | CLU | TG(18:1_17:1_18:3) | 0.137 | -0.431 | 0.139 | 0.208 | 0.982 | -2.066 | 0.329 | 0.042 | 0.950 |
| rs3752246 | ABCA7 | DG(34:4e) | 0.299 | -0.410 | 0.138 | 0.199 | 2.170 | -2.064 | 0.033 | 0.043 | 0.950 |
| rs4147929 | ABCA7 | DG(34:4e) | 0.299 | -0.410 | 0.138 | 0.199 | 2.170 | -2.064 | 0.033 | 0.043 | 0.950 |
| rs9349407 | CD2AP | PE(18:0p_22:4) | 0.346 | -0.435 | 0.150 | 0.211 | 2.298 | -2.064 | 0.024 | 0.043 | 0.950 |
| rs10948363 | CD2AP | PE(18:0p_22:4) | 0.346 | -0.435 | 0.150 | 0.211 | 2.298 | -2.064 | 0.024 | 0.043 | 0.950 |
| rs10792832 | PICALM | ChE(22:5) | -0.121 | 0.425 | 0.138 | 0.206 | -0.872 | 2.063 | 0.386 | 0.043 | 0.950 |
| rs3851179 | PICALM | ChE(22:5) | -0.121 | 0.425 | 0.138 | 0.206 | -0.872 | 2.063 | 0.386 | 0.043 | 0.950 |
| rs6656401 | CR1 | Cer(d18:0_24:1) | 0.189 | -0.430 | 0.147 | 0.209 | 1.286 | -2.062 | 0.202 | 0.043 | 0.950 |
| rs9331896 | CLU | SM(d38:1) | -0.179 | 0.398 | 0.127 | 0.193 | -1.404 | 2.062 | 0.165 | 0.043 | 0.950 |
| rs11767557 | EPHA1 | PC(36:2) | -0.072 | 0.409 | 0.122 | 0.199 | -0.593 | 2.062 | 0.555 | 0.043 | 0.950 |
| rs7561528 | BIN1 | TG(16:1_20:1_22:4) | 0.044 | -0.406 | 0.141 | 0.197 | 0.313 | -2.058 | 0.755 | 0.043 | 0.950 |
| rs12034383 | CR1 | PC(15:0_20:2) | -0.282 | 0.421 | 0.144 | 0.205 | -1.955 | 2.058 | 0.054 | 0.043 | 0.950 |
| rs983392 | MS4A6A | TG(22:5_18:2_18:2) | 0.111 | -0.442 | 0.145 | 0.215 | 0.764 | -2.058 | 0.447 | 0.043 | 0.950 |
| rs11767557 | EPHA1 | TG(18:1_20:2_22:5) | -0.305 | 0.437 | 0.131 | 0.212 | -2.330 | 2.056 | 0.023 | 0.043 | 0.950 |
| rs3865444 | CD33 | TG(17:0_18:2_20:3) | 0.257 | -0.417 | 0.131 | 0.203 | 1.969 | -2.056 | 0.053 | 0.043 | 0.950 |
| rs7274581 | CASS4 | TG(15:0_16:0_16:1) | -0.285 | 0.452 | 0.148 | 0.220 | -1.923 | 2.055 | 0.058 | 0.043 | 0.950 |
| rs9349407 | CD2AP | SM(d34:0) | 0.151 | -0.415 | 0.144 | 0.202 | 1.048 | -2.055 | 0.298 | 0.043 | 0.950 |
| rs10948363 | CD2AP | SM(d34:0) | 0.151 | -0.415 | 0.144 | 0.202 | 1.048 | -2.055 | 0.298 | 0.043 | 0.950 |
| rs11136000 | CLU | SM(d41:3) | -0.157 | 0.423 | 0.138 | 0.206 | -1.137 | 2.052 | 0.259 | 0.044 | 0.950 |
| rs1532278 | CLU | SM(d41:3) | -0.157 | 0.423 | 0.138 | 0.206 | -1.137 | 2.052 | 0.259 | 0.044 | 0.950 |
| rs6656401 | CR1 | SM(d18:1_21:0) | 0.094 | -0.410 | 0.140 | 0.200 | 0.671 | -2.051 | 0.504 | 0.044 | 0.950 |
| rs610932 | MS4A6A | TG(17:0_18:1_20:3) | 0.083 | -0.348 | 0.120 | 0.170 | 0.691 | -2.051 | 0.492 | 0.044 | 0.950 |
| rs1476679 | ZCWPW1 | Cer(m18:1_24:1) | 0.135 | -0.406 | 0.130 | 0.198 | 1.037 | -2.049 | 0.303 | 0.044 | 0.950 |
| rs3752246 | ABCA7 | SM(d42:3) | -0.411 | 0.417 | 0.141 | 0.204 | -2.909 | 2.048 | 0.005 | 0.044 | 0.950 |
| rs4147929 | ABCA7 | SM(d42:3) | -0.411 | 0.417 | 0.141 | 0.204 | -2.909 | 2.048 | 0.005 | 0.044 | 0.950 |
| rs610932 | MS4A6A | PC(32:1) | -0.113 | 0.410 | 0.141 | 0.200 | -0.803 | 2.047 | 0.425 | 0.044 | 0.950 |
| rs9331896 | CLU | SM(d37:1) | -0.226 | 0.432 | 0.139 | 0.211 | -1.625 | 2.047 | 0.108 | 0.044 | 0.950 |
| rs9331896 | CLU | TG(18:2_20:4_22:6) | 0.312 | -0.432 | 0.139 | 0.211 | 2.242 | -2.047 | 0.028 | 0.044 | 0.950 |
| rs11136000 | CLU | Cer(d18:1_24:0) | -0.189 | 0.409 | 0.134 | 0.200 | -1.413 | 2.047 | 0.162 | 0.044 | 0.950 |
| rs1532278 | CLU | Cer(d18:1_24:0) | -0.189 | 0.409 | 0.134 | 0.200 | -1.413 | 2.047 | 0.162 | 0.044 | 0.950 |
| rs17125944 | FERMT2 | Cer(d18:0_23:0) | -0.281 | 0.445 | 0.181 | 0.218 | -1.548 | 2.045 | 0.126 | 0.044 | 0.950 |
| rs610932 | MS4A6A | TG(22:5_18:2_18:2) | 0.217 | -0.440 | 0.151 | 0.215 | 1.434 | -2.045 | 0.156 | 0.044 | 0.950 |
| rs10792832 | PICALM | ChE(22:6) | -0.021 | 0.384 | 0.126 | 0.188 | -0.170 | 2.045 | 0.865 | 0.044 | 0.950 |
| rs3851179 | PICALM | ChE(22:6) | -0.021 | 0.384 | 0.126 | 0.188 | -0.170 | 2.045 | 0.865 | 0.044 | 0.950 |
| rs9331896 | CLU | SM(d32:0) | -0.198 | 0.423 | 0.137 | 0.207 | -1.449 | 2.043 | 0.151 | 0.045 | 0.950 |
| rs610932 | MS4A6A | DG(22:4e) | 0.040 | -0.415 | 0.143 | 0.203 | 0.282 | -2.043 | 0.779 | 0.045 | 0.950 |
| rs744373 | BIN1 | TG(20:1_14:1_22:4) | 0.286 | -0.441 | 0.149 | 0.216 | 1.924 | -2.042 | 0.058 | 0.045 | 0.950 |
| rs983392 | MS4A6A | TG(18:1_18:2_22:0) | 0.112 | -0.406 | 0.134 | 0.199 | 0.834 | -2.041 | 0.407 | 0.045 | 0.950 |
| rs11136000 | CLU | SM(d34:2) | -0.099 | 0.420 | 0.138 | 0.206 | -0.721 | 2.041 | 0.473 | 0.045 | 0.950 |
| rs1532278 | CLU | SM(d34:2) | -0.099 | 0.420 | 0.138 | 0.206 | -0.721 | 2.041 | 0.473 | 0.045 | 0.950 |
| rs1476679 | ZCWPW1 | Cer(m18:0_22:0) | 0.170 | -0.407 | 0.131 | 0.199 | 1.299 | -2.041 | 0.198 | 0.045 | 0.950 |
| rs17125944 | FERMT2 | TG(18:1_17:1_18:2) | -0.356 | 0.409 | 0.167 | 0.200 | -2.134 | 2.040 | 0.036 | 0.045 | 0.950 |
| rs1476679 | ZCWPW1 | TG(18:4_14:0_16:1) | 0.268 | -0.442 | 0.142 | 0.217 | 1.883 | -2.040 | 0.064 | 0.045 | 0.950 |
| rs10792832 | PICALM | Cer(d18:0_22:0) | -0.131 | 0.421 | 0.139 | 0.207 | -0.941 | 2.039 | 0.350 | 0.045 | 0.950 |
| rs3851179 | PICALM | Cer(d18:0_22:0) | -0.131 | 0.421 | 0.139 | 0.207 | -0.941 | 2.039 | 0.350 | 0.045 | 0.950 |
| rs610932 | MS4A6A | SM(d40:4) | 0.047 | -0.382 | 0.132 | 0.188 | 0.357 | -2.038 | 0.722 | 0.045 | 0.950 |
| rs3752246 | ABCA7 | DG(18:1_18:1) | 0.200 | -0.397 | 0.135 | 0.195 | 1.484 | -2.038 | 0.142 | 0.045 | 0.950 |
| rs4147929 | ABCA7 | DG(18:1_18:1) | 0.200 | -0.397 | 0.135 | 0.195 | 1.484 | -2.038 | 0.142 | 0.045 | 0.950 |
| rs3752246 | ABCA7 | TG(16:1_12:0_18:1) | 0.203 | -0.437 | 0.149 | 0.214 | 1.368 | -2.037 | 0.175 | 0.045 | 0.950 |
| rs4147929 | ABCA7 | TG(16:1_12:0_18:1) | 0.203 | -0.437 | 0.149 | 0.214 | 1.368 | -2.037 | 0.175 | 0.045 | 0.950 |
| rs9331896 | CLU | Cer(d18:1_23:0) | -0.199 | 0.391 | 0.127 | 0.192 | -1.572 | 2.036 | 0.120 | 0.045 | 0.950 |
| rs561655 | PICALM | SM(d35:4) | -0.010 | 0.411 | 0.137 | 0.202 | -0.072 | 2.036 | 0.943 | 0.045 | 0.950 |
| rs6733839 | BIN1 | TG(55:5) | 0.344 | -0.430 | 0.138 | 0.211 | 2.484 | -2.036 | 0.015 | 0.045 | 0.950 |
| rs7274581 | CASS4 | PC(16:0_20:4) | 0.175 | -0.439 | 0.145 | 0.216 | 1.208 | -2.035 | 0.231 | 0.045 | 0.950 |
| rs10498633 | SLC24A4 | TG(18:0_18:1_24:0) | 0.424 | -0.471 | 0.186 | 0.232 | 2.282 | -2.035 | 0.025 | 0.045 | 0.950 |
| rs11136000 | CLU | TG(26:1_18:1_18:2) | 0.078 | -0.423 | 0.139 | 0.208 | 0.565 | -2.035 | 0.574 | 0.045 | 0.950 |
| rs1532278 | CLU | TG(26:1_18:1_18:2) | 0.078 | -0.423 | 0.139 | 0.208 | 0.565 | -2.035 | 0.574 | 0.045 | 0.950 |
| rs744373 | BIN1 | TG(55:5) | 0.360 | -0.425 | 0.144 | 0.209 | 2.504 | -2.033 | 0.014 | 0.046 | 0.950 |
| rs6733839 | BIN1 | DG(34:4e) | 0.016 | -0.408 | 0.131 | 0.201 | 0.123 | -2.032 | 0.903 | 0.046 | 0.950 |
| rs744373 | BIN1 | SM(t42:1) | -0.230 | 0.424 | 0.143 | 0.209 | -1.604 | 2.032 | 0.113 | 0.046 | 0.950 |
| rs35349669 | INPP5D | Cer(d19:1_24:1) | 0.029 | -0.410 | 0.144 | 0.202 | 0.199 | -2.032 | 0.843 | 0.046 | 0.950 |
| rs561655 | PICALM | TG(14:0e_18:0_20:1) | -0.289 | 0.433 | 0.145 | 0.213 | -1.989 | 2.030 | 0.050 | 0.046 | 0.950 |
| rs17125944 | FERMT2 | TG(18:1_18:2_24:1) | -0.434 | 0.451 | 0.185 | 0.222 | -2.345 | 2.026 | 0.022 | 0.046 | 0.950 |
| rs3818361 | CR1 | TG(18:4_16:0_16:1) | 0.186 | -0.428 | 0.155 | 0.211 | 1.201 | -2.025 | 0.234 | 0.046 | 0.950 |
| rs6701713 | CR1 | TG(18:4_16:0_16:1) | 0.186 | -0.428 | 0.155 | 0.211 | 1.201 | -2.025 | 0.234 | 0.046 | 0.950 |
| rs3752246 | ABCA7 | SM(d32:0) | -0.373 | 0.405 | 0.139 | 0.200 | -2.688 | 2.025 | 0.009 | 0.047 | 0.950 |
| rs4147929 | ABCA7 | SM(d32:0) | -0.373 | 0.405 | 0.139 | 0.200 | -2.688 | 2.025 | 0.009 | 0.047 | 0.950 |
| rs744373 | BIN1 | TG(22:5_18:2_18:2) | 0.126 | -0.438 | 0.149 | 0.216 | 0.849 | -2.024 | 0.399 | 0.047 | 0.950 |
| rs11136000 | CLU | TG(18:0_18:0_18:0) | 0.019 | -0.408 | 0.135 | 0.202 | 0.144 | -2.024 | 0.886 | 0.047 | 0.950 |
| rs1532278 | CLU | TG(18:0_18:0_18:0) | 0.019 | -0.408 | 0.135 | 0.202 | 0.144 | -2.024 | 0.886 | 0.047 | 0.950 |
| rs983392 | MS4A6A | SM(d42:3) | -0.159 | 0.425 | 0.142 | 0.210 | -1.120 | 2.023 | 0.266 | 0.047 | 0.950 |
| rs17125944 | FERMT2 | TG(26:1_18:1_18:1) | -0.444 | 0.465 | 0.191 | 0.230 | -2.318 | 2.023 | 0.023 | 0.047 | 0.950 |
| rs9349407 | CD2AP | SM(d41:1) | 0.156 | -0.394 | 0.139 | 0.195 | 1.122 | -2.023 | 0.266 | 0.047 | 0.950 |
| rs10948363 | CD2AP | SM(d41:1) | 0.156 | -0.394 | 0.139 | 0.195 | 1.122 | -2.023 | 0.266 | 0.047 | 0.950 |
| rs28834970 | PTK2B | PC(44:5) | 0.261 | -0.431 | 0.151 | 0.213 | 1.732 | -2.023 | 0.087 | 0.047 | 0.950 |
| rs9331896 | CLU | SM(d17:1_18:3) | -0.184 | 0.404 | 0.132 | 0.200 | -1.391 | 2.022 | 0.168 | 0.047 | 0.950 |
| rs3752246 | ABCA7 | DG(20:5_18:2) | 0.219 | -0.393 | 0.135 | 0.194 | 1.622 | -2.022 | 0.109 | 0.047 | 0.950 |
| rs4147929 | ABCA7 | DG(20:5_18:2) | 0.219 | -0.393 | 0.135 | 0.194 | 1.622 | -2.022 | 0.109 | 0.047 | 0.950 |
| rs17125944 | FERMT2 | TG(18:0_16:0_19:0) | -0.247 | 0.453 | 0.187 | 0.224 | -1.326 | 2.021 | 0.189 | 0.047 | 0.950 |
| rs17125944 | FERMT2 | TG(16:0_20:4_22:6) | 0.144 | -0.459 | 0.189 | 0.227 | 0.760 | -2.021 | 0.450 | 0.047 | 0.950 |
| rs11136000 | CLU | PE(16:0p_18:1) | -0.173 | 0.443 | 0.147 | 0.219 | -1.181 | 2.020 | 0.241 | 0.047 | 0.950 |
| rs1532278 | CLU | PE(16:0p_18:1) | -0.173 | 0.443 | 0.147 | 0.219 | -1.181 | 2.020 | 0.241 | 0.047 | 0.950 |
| rs1131497 | SORL1 | TG(28:0_16:0_18:1) | -0.099 | 0.445 | 0.143 | 0.221 | -0.692 | 2.018 | 0.491 | 0.047 | 0.950 |
| rs10498633 | SLC24A4 | Cer(d16:0_24:1) | -0.227 | 0.452 | 0.179 | 0.224 | -1.264 | 2.016 | 0.210 | 0.047 | 0.950 |
| rs3818361 | CR1 | Cer(d18:0_24:0) | 0.136 | -0.417 | 0.152 | 0.207 | 0.899 | -2.016 | 0.371 | 0.047 | 0.950 |
| rs6701713 | CR1 | Cer(d18:0_24:0) | 0.136 | -0.417 | 0.152 | 0.207 | 0.899 | -2.016 | 0.371 | 0.047 | 0.950 |
| rs744373 | BIN1 | TG(16:0_12:0_17:1) | -0.084 | 0.410 | 0.140 | 0.203 | -0.601 | 2.015 | 0.550 | 0.048 | 0.950 |
| rs11136000 | CLU | SM(d32:0) | -0.200 | 0.415 | 0.138 | 0.206 | -1.448 | 2.014 | 0.152 | 0.048 | 0.950 |
| rs1532278 | CLU | SM(d32:0) | -0.200 | 0.415 | 0.138 | 0.206 | -1.448 | 2.014 | 0.152 | 0.048 | 0.950 |
| rs10838725 | CELF1 | PI(18:0_20:4) | 0.308 | -0.407 | 0.136 | 0.202 | 2.255 | -2.013 | 0.027 | 0.048 | 0.950 |
| rs11218343 | SORL1 | PE(18:0p_18:1) | -0.342 | 0.462 | 0.184 | 0.229 | -1.856 | 2.012 | 0.067 | 0.048 | 0.950 |
| rs983392 | MS4A6A | TG(16:0_16:0_17:0) | -0.099 | 0.435 | 0.146 | 0.216 | -0.677 | 2.012 | 0.501 | 0.048 | 0.950 |
| rs28834970 | PTK2B | SM(t42:1) | 0.308 | -0.426 | 0.150 | 0.212 | 2.056 | -2.011 | 0.043 | 0.048 | 0.950 |
| rs12034383 | CR1 | PC(36:6) | -0.245 | 0.418 | 0.147 | 0.208 | -1.670 | 2.011 | 0.099 | 0.048 | 0.950 |
| rs744373 | BIN1 | TG(15:0_16:0_20:5) | 0.024 | -0.396 | 0.135 | 0.197 | 0.181 | -2.011 | 0.857 | 0.048 | 0.950 |
| rs6656401 | CR1 | PE(18:0_18:1) | 0.279 | -0.417 | 0.146 | 0.208 | 1.913 | -2.010 | 0.060 | 0.048 | 0.950 |
| rs744373 | BIN1 | TG(16:0_18:1_19:0) | 0.092 | -0.366 | 0.125 | 0.182 | 0.737 | -2.010 | 0.464 | 0.048 | 0.950 |
| rs10792832 | PICALM | TG(14:0e_18:0_20:1) | -0.206 | 0.431 | 0.144 | 0.214 | -1.432 | 2.010 | 0.156 | 0.048 | 0.950 |
| rs3851179 | PICALM | TG(14:0e_18:0_20:1) | -0.206 | 0.431 | 0.144 | 0.214 | -1.432 | 2.010 | 0.156 | 0.048 | 0.950 |
| rs7274581 | CASS4 | TG(15:0_16:0_16:0) | -0.292 | 0.446 | 0.149 | 0.222 | -1.952 | 2.009 | 0.055 | 0.048 | 0.950 |
| rs983392 | MS4A6A | PC(20:2_18:2) | -0.080 | 0.381 | 0.128 | 0.190 | -0.628 | 2.008 | 0.532 | 0.048 | 0.950 |
| rs9331896 | CLU | PC(33:2) | -0.202 | 0.430 | 0.141 | 0.214 | -1.426 | 2.008 | 0.158 | 0.048 | 0.950 |
| rs17125944 | FERMT2 | DG(20:0_18:2) | -0.348 | 0.397 | 0.164 | 0.198 | -2.118 | 2.008 | 0.038 | 0.048 | 0.950 |
| rs983392 | MS4A6A | TG(15:0_16:1_20:5) | -0.054 | -0.384 | 0.129 | 0.192 | -0.421 | -2.007 | 0.675 | 0.048 | 0.950 |
| rs6733839 | BIN1 | TG(18:3_18:2_22:5) | 0.081 | -0.396 | 0.129 | 0.198 | 0.628 | -2.006 | 0.532 | 0.048 | 0.950 |
| rs10838725 | CELF1 | TG(15:0_16:0_20:5) | -0.128 | 0.401 | 0.135 | 0.200 | -0.951 | 2.006 | 0.345 | 0.049 | 0.950 |
| rs9331896 | CLU | TG(18:1_18:2_23:0) | 0.019 | -0.399 | 0.131 | 0.199 | 0.148 | -2.004 | 0.883 | 0.049 | 0.950 |
| rs7561528 | BIN1 | TG(12:0_12:0_14:0) | -0.303 | 0.441 | 0.157 | 0.220 | -1.926 | 2.004 | 0.058 | 0.049 | 0.950 |
| rs10498633 | SLC24A4 | TG(16:0_17:1_20:5) | -0.243 | 0.435 | 0.174 | 0.217 | -1.396 | 2.004 | 0.167 | 0.049 | 0.950 |
| rs9349407 | CD2AP | TG(18:4_16:0_16:1) | -0.224 | 0.426 | 0.152 | 0.213 | -1.475 | 2.003 | 0.145 | 0.049 | 0.950 |
| rs10948363 | CD2AP | TG(18:4_16:0_16:1) | -0.224 | 0.426 | 0.152 | 0.213 | -1.475 | 2.003 | 0.145 | 0.049 | 0.950 |
| rs1476679 | ZCWPW1 | Cer(m18:0_24:1) | 0.183 | -0.407 | 0.134 | 0.203 | 1.372 | -2.002 | 0.174 | 0.049 | 0.950 |
| rs10792832 | PICALM | Cer(d18:0_23:0) | -0.018 | 0.390 | 0.131 | 0.195 | -0.138 | 2.002 | 0.890 | 0.049 | 0.950 |
| rs3851179 | PICALM | Cer(d18:0_23:0) | -0.018 | 0.390 | 0.131 | 0.195 | -0.138 | 2.002 | 0.890 | 0.049 | 0.950 |
| rs744373 | BIN1 | TG(16:0_8:0_14:0) | -0.242 | 0.437 | 0.150 | 0.218 | -1.612 | 2.000 | 0.111 | 0.049 | 0.950 |
| rs11136000 | CLU | PE(18:0_18:1) | -0.004 | -0.411 | 0.138 | 0.206 | -0.033 | -1.999 | 0.974 | 0.049 | 0.950 |
| rs1532278 | CLU | PE(18:0_18:1) | -0.004 | -0.411 | 0.138 | 0.206 | -0.033 | -1.999 | 0.974 | 0.049 | 0.950 |
| rs983392 | MS4A6A | TG(15:0_18:1_22:6) | -0.268 | 0.406 | 0.137 | 0.203 | -1.953 | 1.998 | 0.055 | 0.049 | 0.950 |
| rs3752246 | ABCA7 | DG(18:2_18:2) | 0.249 | -0.399 | 0.139 | 0.200 | 1.801 | -1.997 | 0.076 | 0.049 | 0.950 |
| rs4147929 | ABCA7 | DG(18:2_18:2) | 0.249 | -0.399 | 0.139 | 0.200 | 1.801 | -1.997 | 0.076 | 0.049 | 0.950 |
| rs10792832 | PICALM | TG(16:0_16:0_20:4) | -0.202 | 0.391 | 0.132 | 0.196 | -1.540 | 1.997 | 0.128 | 0.049 | 0.950 |
| rs3851179 | PICALM | TG(16:0_16:0_20:4) | -0.202 | 0.391 | 0.132 | 0.196 | -1.540 | 1.997 | 0.128 | 0.049 | 0.950 |
| rs28834970 | PTK2B | SM(d28:1) | 0.346 | -0.421 | 0.149 | 0.211 | 2.314 | -1.996 | 0.023 | 0.050 | 0.950 |
| rs610932 | MS4A6A | PE(16:0p_22:6) | 0.000 | 0.409 | 0.144 | 0.205 | 0.003 | 1.994 | 0.998 | 0.050 | 0.950 |

Beta.SNP/SE.SNP/ t.SNP/ Pval.SNP: Beta coefficient/standard error/t statistics/p value of association of SNPs with individual lipids.

Beta.SNP_CC/SE.SNP_CC/t.SNP_CC/Pval.SNP_CC: Beta coefficient/standard error/t statistics/p value of differential association of SNPs with lipids between AD and controls

Cer: ceramides; SM: sphingomyelins; ChE: Cholesteryl esters; DG: diglycerides; TG: triglycerides; PC: phosphatidylcholines; LPC: lyso- phosphatidylcholines; PE: phosphatidylethanolamines; PI: phosphatidylinositols
